# Supplementary material for: Macrophages regulate vascular smooth muscle cell function during atherosclerosis progression through IL-1β/STAT3 signaling
Source: Commun Biol. 2022 Dec 1;5:1316. doi: 10.1038/s42003-022-04255-2 (PMC9715630; doi:10.1038/s42003-022-04255-2)

Supplementary information

Macrophages regulate vascular smooth muscle cell function during atherosclerosis progression through IL-1β/STAT3 signaling

Yuzhou Xue [1 2], Minghao Luo [1], Xiankang Hu [1], Xiang Li [1], Jian Shen [1], Yanwen Zhu [34], Longxiang Huang [1], Yu Hu [1], Yongzheng Guo [1], Lin Liu [5], Lingbang Wang [6], Suxin Luo [1]

1Department of Cardiology, the First Affiliated Hospital of Chongqing Medical University, Chongqing, China.

2 Department of Cardiology and Institute of Vascular Medicine, Peking University Third Hospital, Beijing, China.

3Medical Department, Yidu Cloud (Beijing) Technology Co., Ltd., Beijing, China.

4 Chongqing Engineering Research Center of Pharmaceutical Sciences, Chongqing Medical and Pharmaceutical College, Chongqing, China.

5 Department of Dermatology, the First Affiliated Hospital of Chongqing Medical University, Chongqing, China.

6 Department of Orthopedic Surgery, the First Affiliated Hospital of Chongqing Medical University, Chongqing, China.

Yuzhou Xue and Minghao Luo contributed equally in this paper.

CONTENT

1. Supplementary Figure 1-11

2. original blots

3. original blots with molecular marker ladders

1. Supplementary Figure 1-11

Supplementary Figure 1

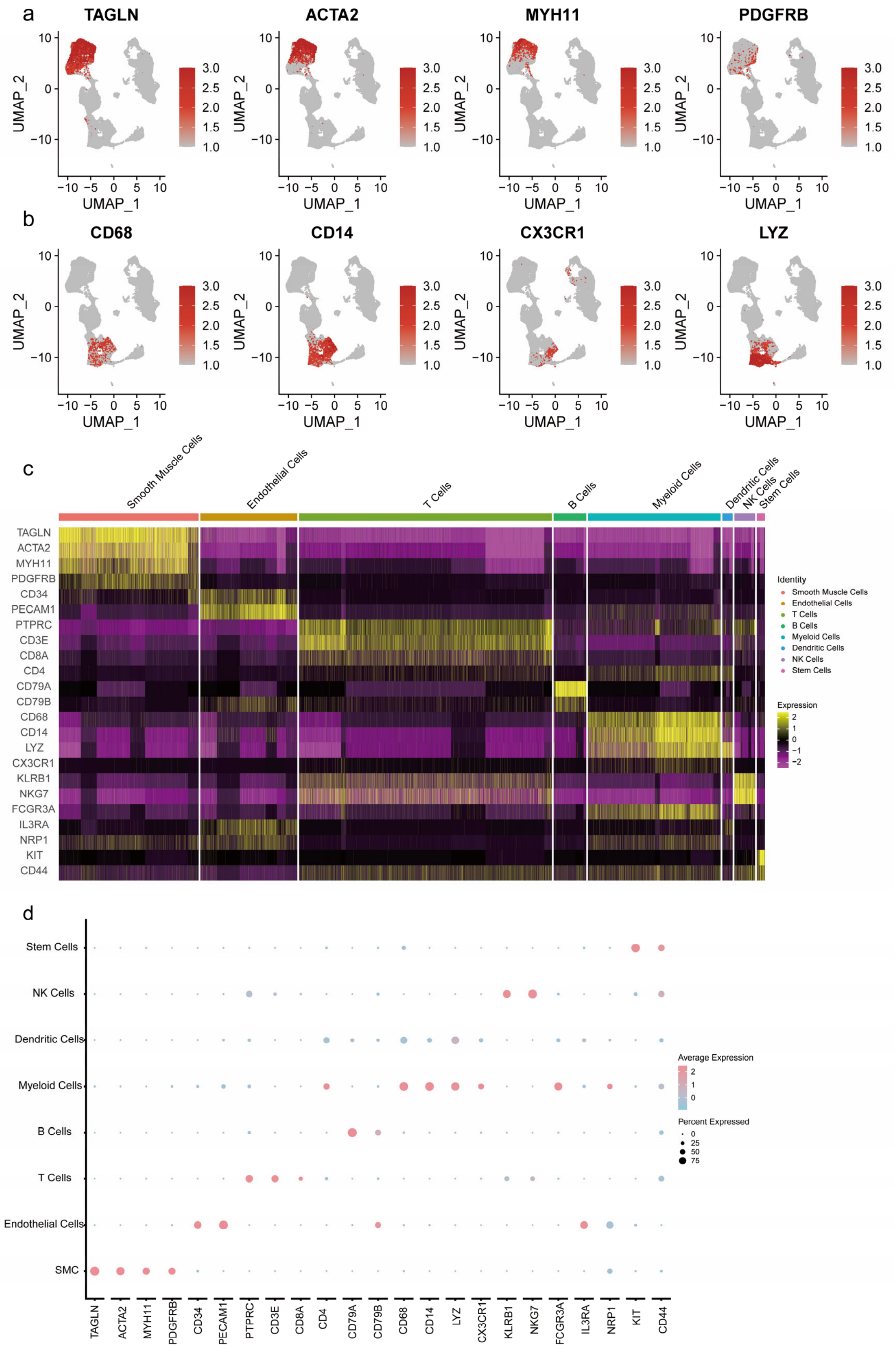

Supplementary Figure 1. scRNA-seq analysis revealed 8 distinct cell types. Feature plots of cell marker genes of (**a**) vascular smooth muscle cells (VSMCs) and (**b**) myeloid cells among

different cell clusters. **c** Heatmap of top marker gens per cell type. d Dot plots showing the expression and percent expressed of marker genes in every cell type.

Supplementary Figure 2

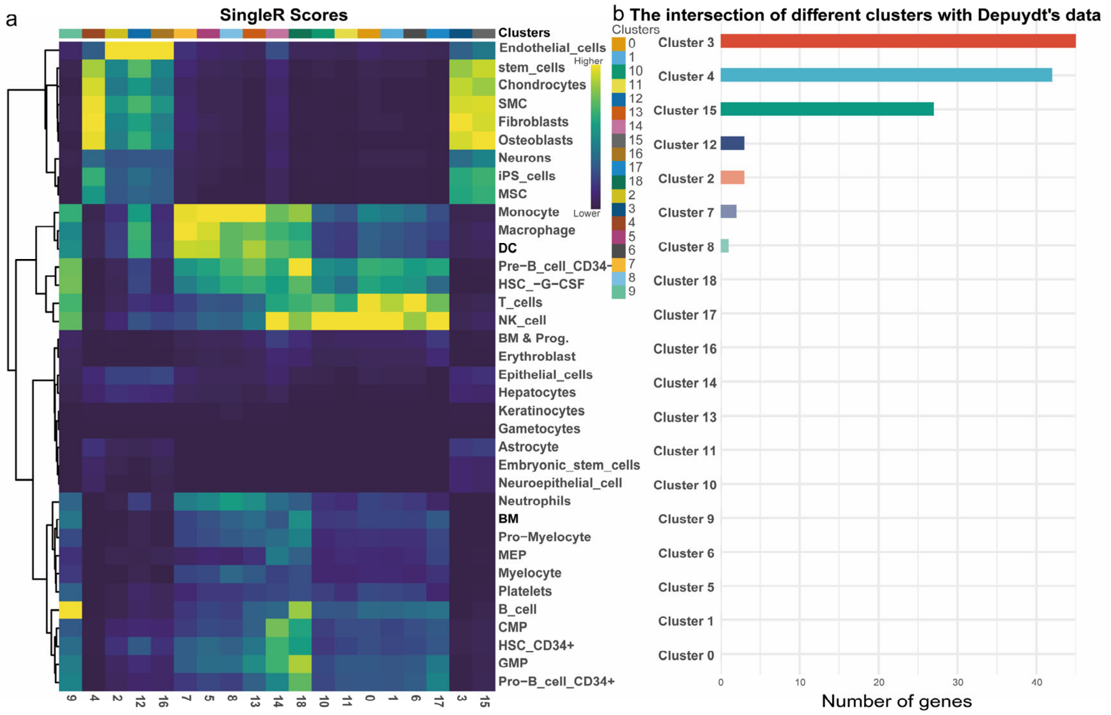

Supplementary Figure 2. Validation the identity of vascular smooth muscle cell (VSMC) clusters. **a**. Different population identities by similarity to known cell type in Human Primary Cell Atlas (HPCA) reference RNA-seq data sets through SingleR analysis. **b**. intersection analysis of top markers among different clusters with top marker genes in SMC subset identified in Depuydt's data.

Supplementary Figure 3

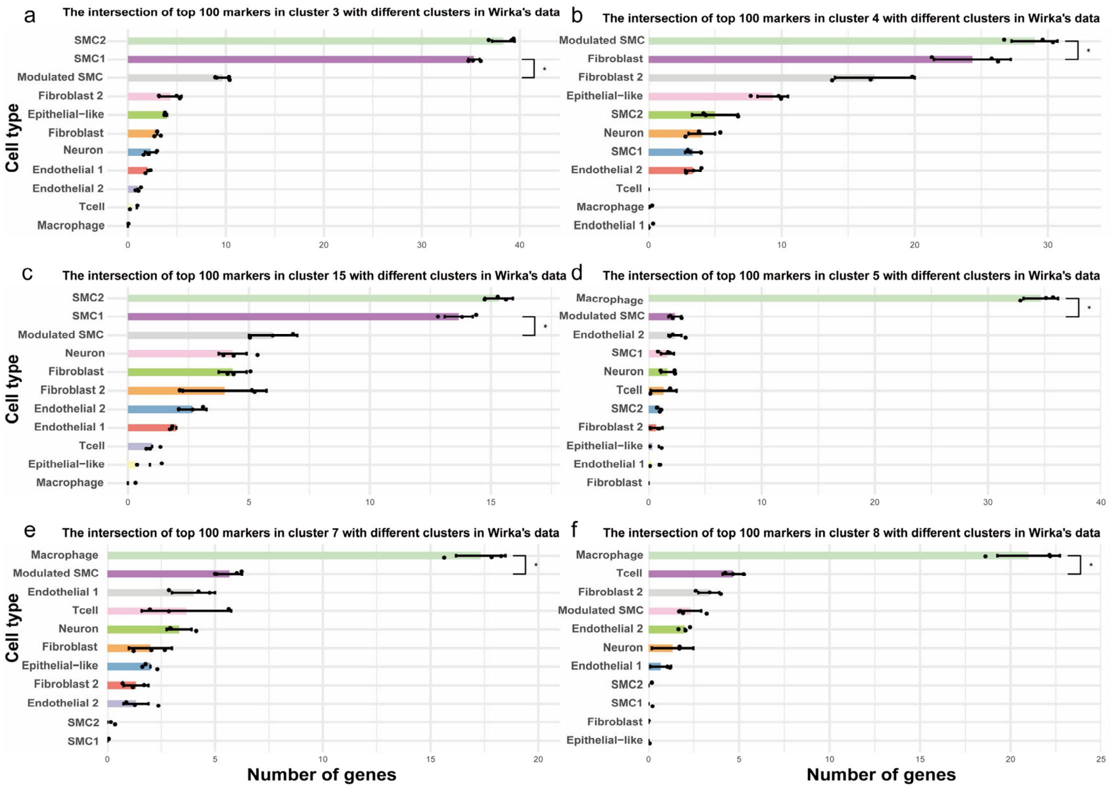

Supplementary Figure 3. Validation the identity of vascular smooth muscle cell (VSMC) and myeloid clusters. The intersection analysis of top 100 marker genes in (**a**) cluster 3, (**b**) cluster 4, (**c**) cluster 15, (**d**) cluster 5, (**e**) cluster 7, and (**f**) cluster 8 with top 100 marker genes in different cell type identified in Wirka's data. Wilcoxon signed-rank test: *$P < 0.05$.

Supplementary Figure 4

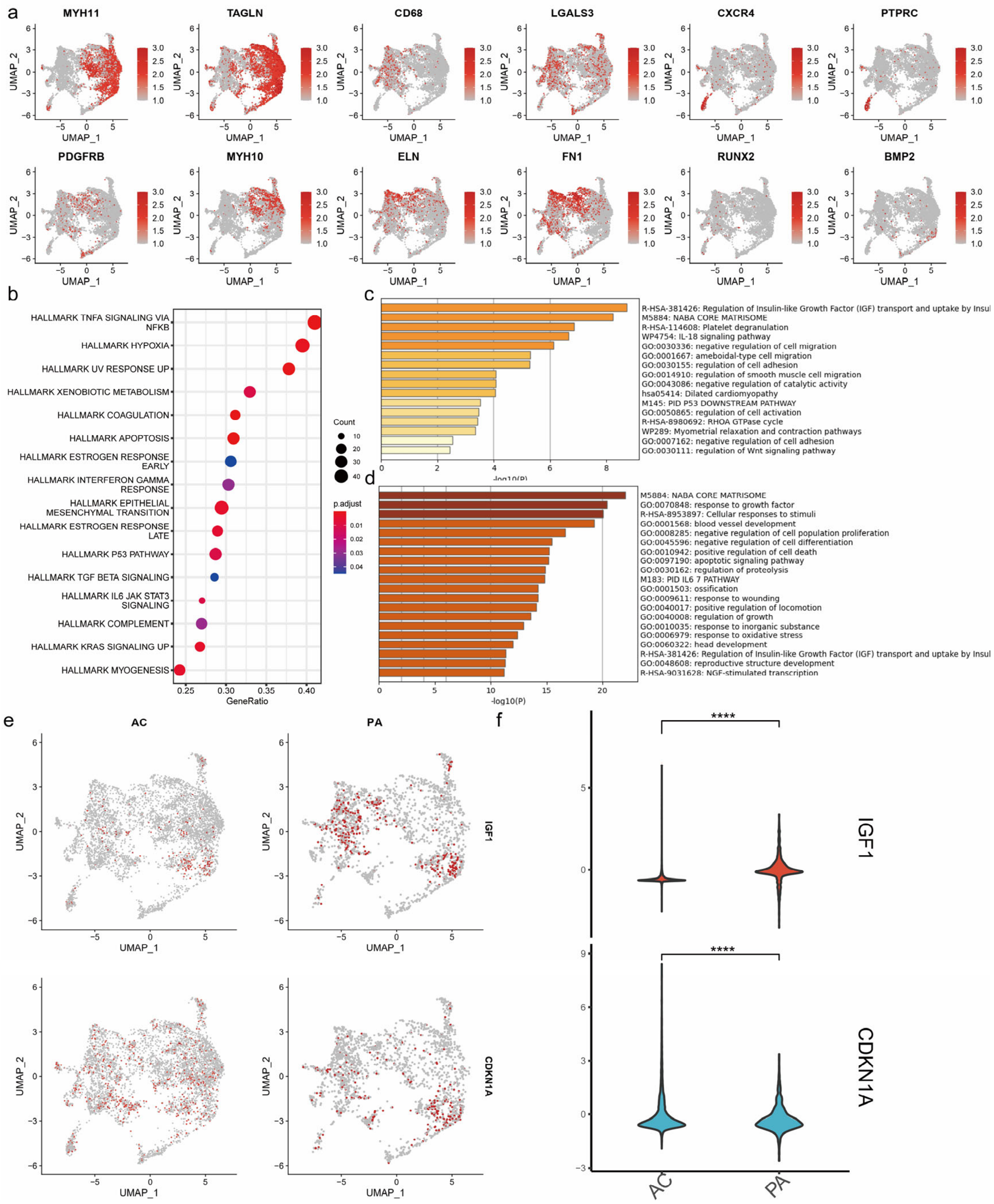

Supplementary Figure 4. Sub-clustering of vascular smooth muscle cells (VSMCs). **a**. Feature plots of cell marker genes among different VSMC subsets. **b**. The hallmark gene sets of gene set enrichment analysis (GSEA) of VSMCs between proximal adjacent (PA) and atherosclerotic core (AC) group. Enrichment analysis of upregulated (**c**) and downregulated (**d**) genes of VSMCs in the AC group. **e**. Feature plots of *IGF-1* and *CDKN1A* among PA and AC group. **f**. Violin plots of *IGF-1*, *CDKN1A*, and *CDKN2B* in PA and AC group. Two-sample t test: ***$P < 0.001$, ****$P<0.0001$.

Supplementary Figure 5

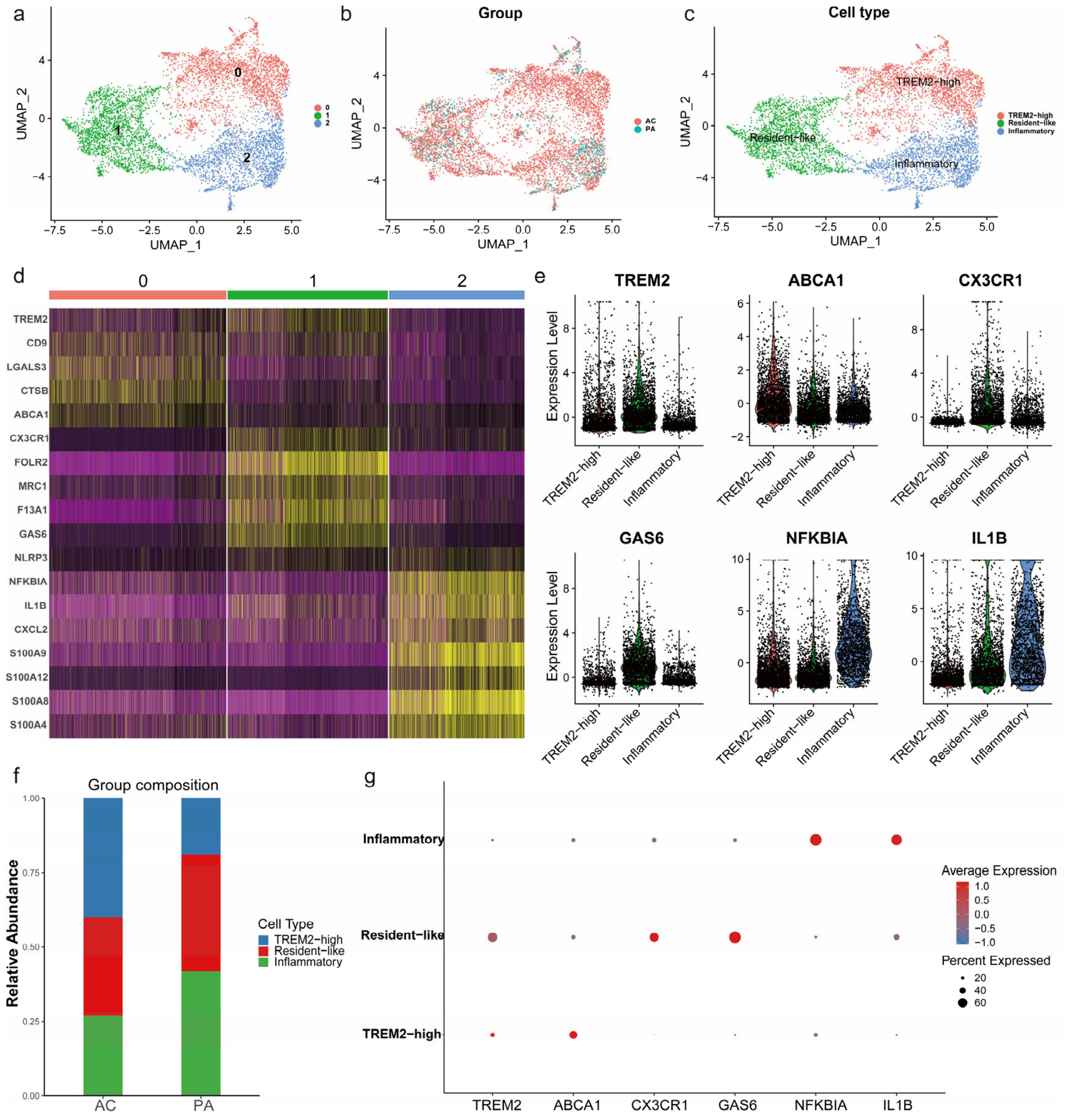

Supplementary Figure 5. Diversity of the myeloid cells in proximal adjacent (PA) and calcified atherosclerotic core (AC) portions of plaques. UMAP clustering plots revealing (**a**) 3 color-coded cell clusters, (**b**) different groups, and (**c**) 3 cell population of a total of 6694 individual cells based on gene expression. **d**. Heatmap of marker genes of different macrophage subsets per clusters. **e**. Violin plots of signature genes confirmed population identities. **f**. Bar chart of the relative frequency of myeloid cell subtypes in PA and AC groups. **g**. Dot plot shows the expression and percent expressed of cluster-identifying genes in myeloid cells.

Supplementary Figure 6

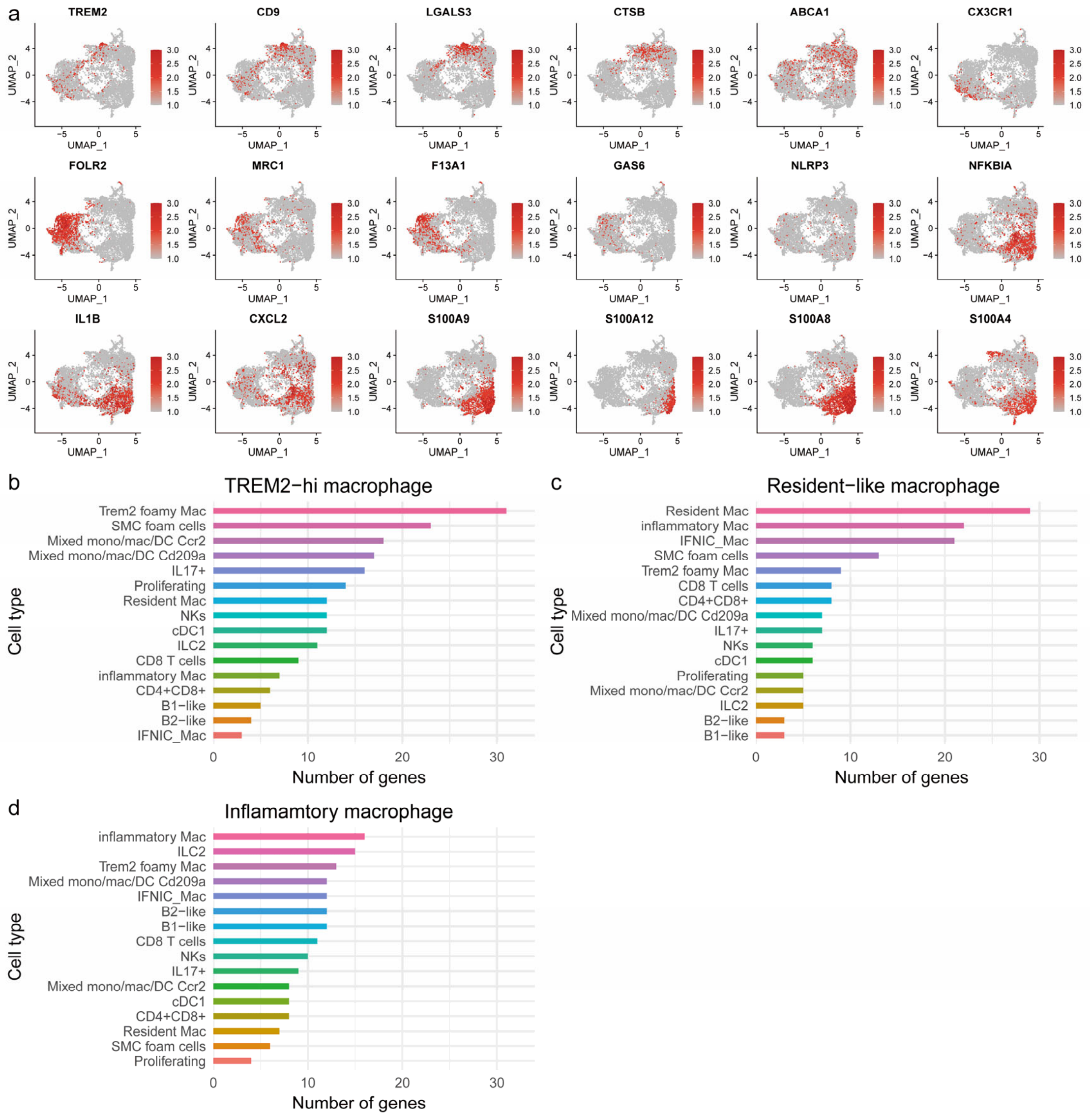

Supplementary Figure 6. Sub-clustering and validation of myeloid Subsets. **a**. Feature plots of cell marker genes of different myeloid subtypes among clusters. The intersection analysis of top 100 marker genes in (**b**) TREM2-hi macrophages, (**c**) Resident-like macrophages, and (**d**) Inflammatory macrophages with top 100 marker genes in different cell type identified in Zernecke's data

Supplementary Figure 7

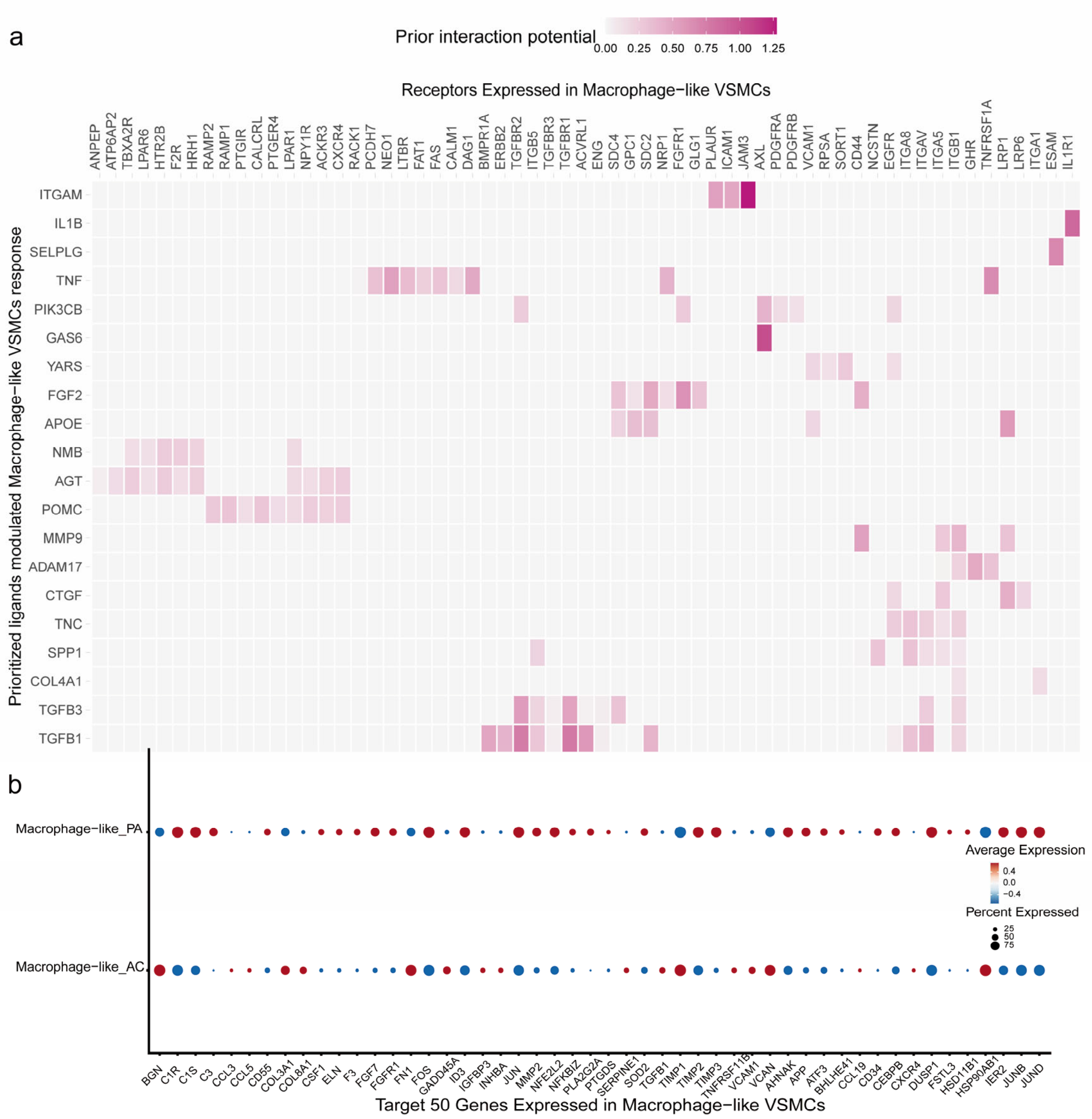

Supplementary Figure 7. NicheNet analysis of macrophages and macrophage-like vascular smooth muscle cell (VSMCs). **a**. Ligand-receptor heatmap of potential receptors expressed by macrophage-like VSMCs interacting with macrophage ligands. **b**. Dot plot confirming the identified target genes in macrophage-like VSMCs.

Supplementary Figure 8

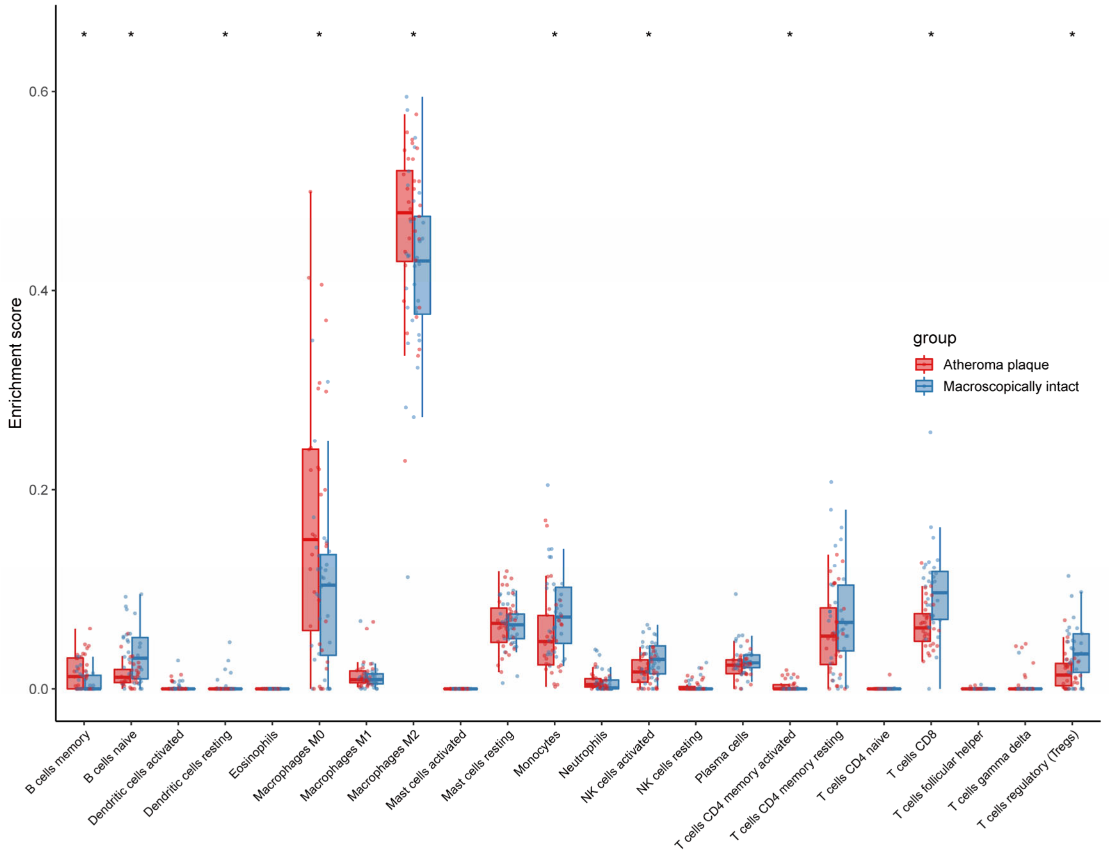

Supplementary Figure 8. The estimated proportions of immune cells of the atheroma plaque and macroscopically intact tissue group in GSE43292 using CIBERSORT analysis. Wilcoxon signed-rank test (paired): *$P < 0.05$.

Supplementary Figure 9

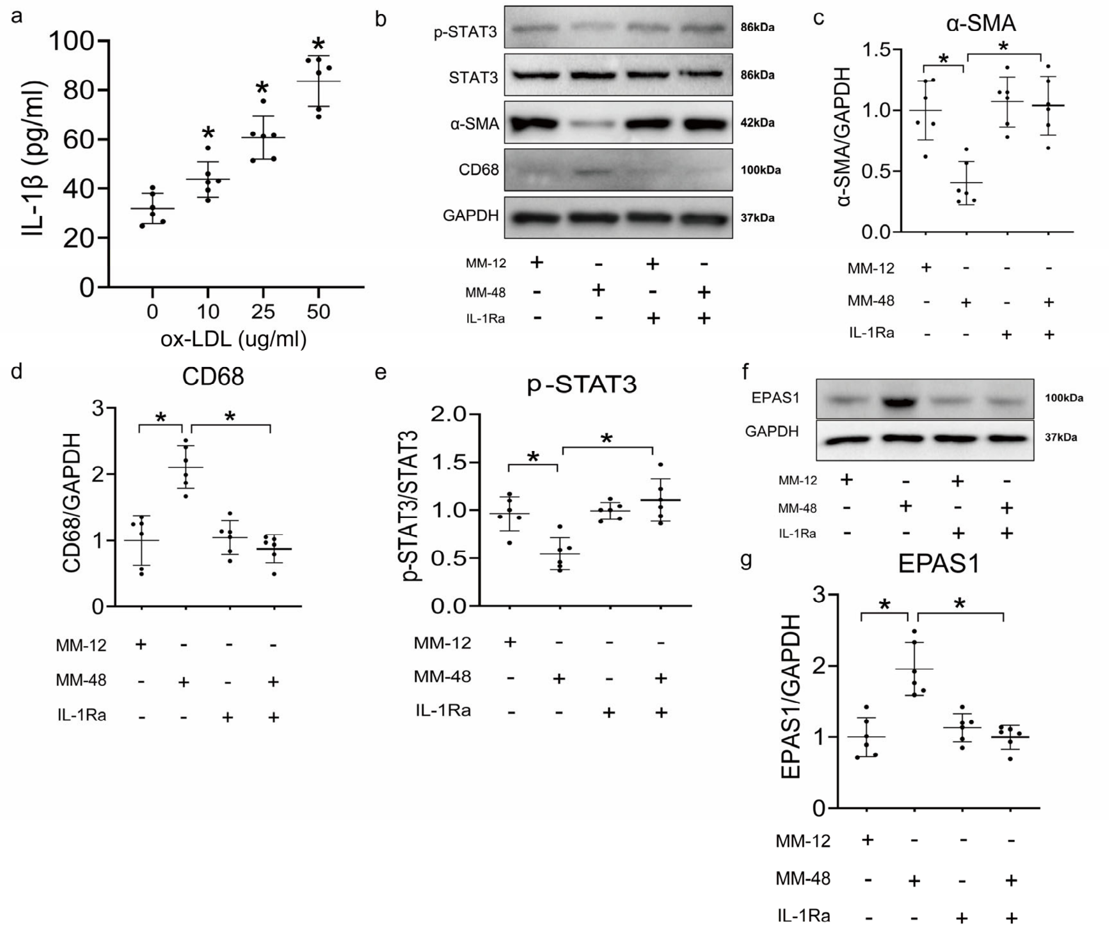

Supplementary Figure 9. IL-1β/STAT3 axis involved in the crosstalk between primary macrophages and macrophage-like vascular smooth muscle cell (VSMCs). **a**. Enzyme-linked immunosorbent assay (ELISA) validates the increased secretion of IL-1β in primary macrophage with ox-LDL treatment. **b**. Western blot assay indicates the expressions of VSMC marker (α-SMA), macrophage marker (CD68), and p-STAT3. The expression of (**c**) α-SMA, (**d**) CD68, and (**e**) p-STAT3 dysregulated by macrophage medium treatment can be reversed by interleukin-1 receptor antagonist (IL-1Ra). **f**. Western blot assay indicates the expressions of EPAS1. The expression of (**g**) EPAS1 dysregulated by macrophage medium treatment can be reversed by interleukin-1 receptor antagonist (IL-1Ra). N = 4-8 for each group in cell experiments as datapoints showing. Data are presented as mean ± SE. Mann-Whitney test: *P<0.05.

Supplementary Figure 10

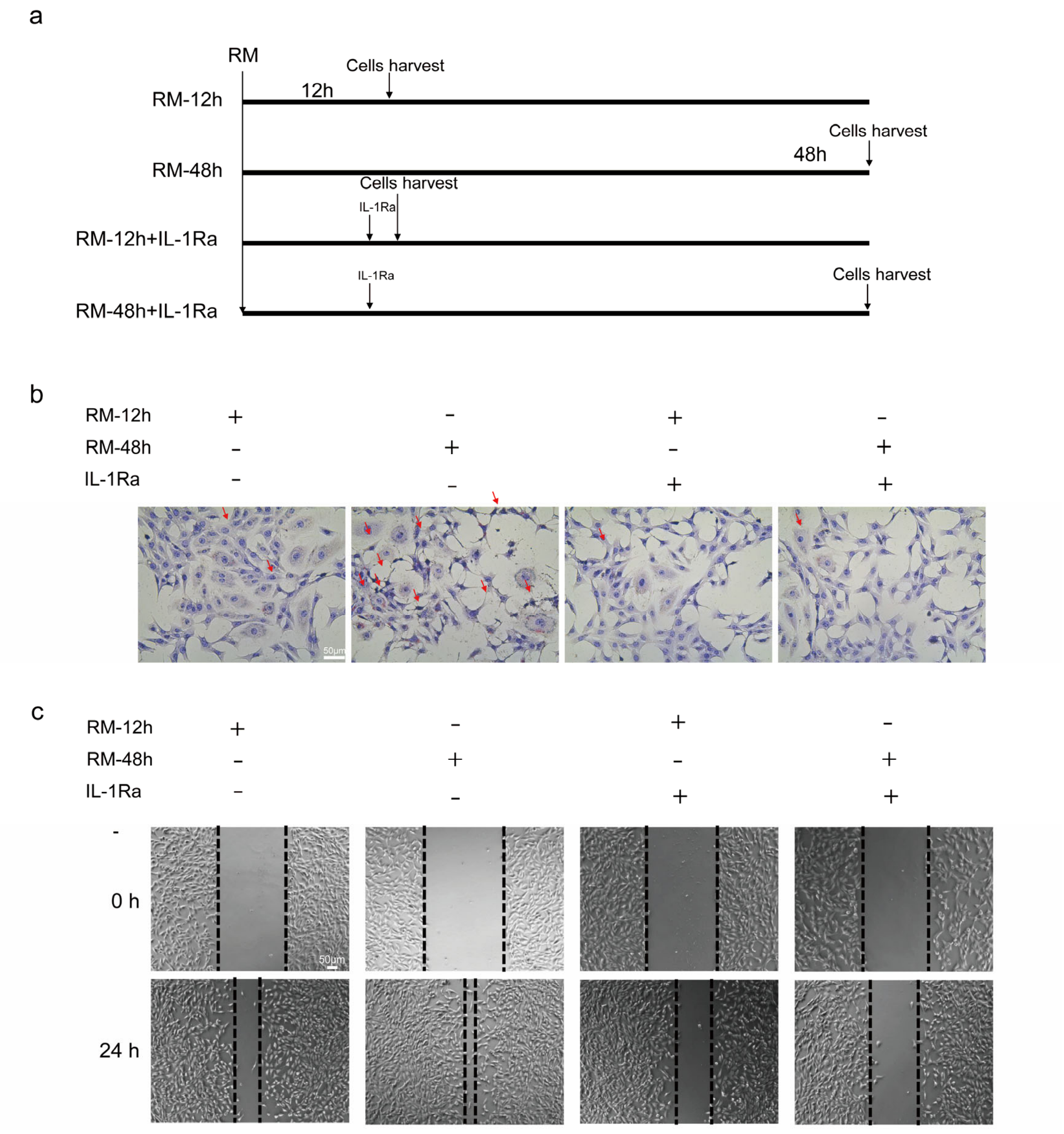

Supplementary Figure 10. Functional experiments indicate IL-1β contributes lipid accumulation and cell migration of macrophage-like vascular smooth muscle cell (VSMC). **a**. Schematic of RAW264.7 medium (RM) and interleukin-1 receptor antagonist (IL-1Ra) treatment in functional experiments. **b**. Representative images of Oil red O staining in macrophage-like VSMCs. **c**. Representative images of migration rates of macrophage-like VSMCs through wound healing assay.

Supplementary Figure 11

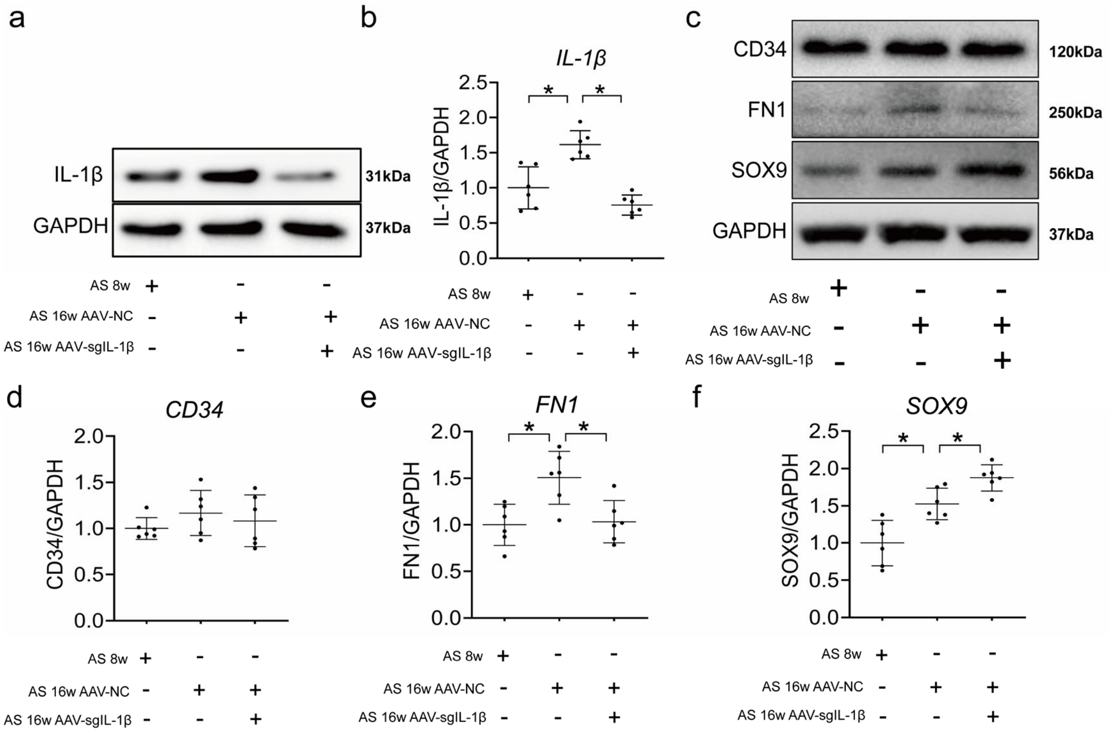

Supplementary Figure 11. *In vivo* experiments identify the correlations of IL-1β with other SMC subsets markers/transcription factors in aorta of mice. **a.** Western blot assays illustrate the effect of adeno-associated virus-sgIL-1β (AAV-sgIL-1β) on the expression of IL-1β *in vivo*. Quantifications of (**b**) IL-1β in aorta of mice from each group. **c.** Western blot assays illustrate the effect of adeno-associated virus-sgIL-1β (AAV-sgIL-1β) on the expression of other SMC subsets transcription factors *in vivo*. Quantifications of (**d**) CD34, (**e**) FN1, (**f**) SOX9 in aorta of mice from each group. Eighteen $ApoE^{-/-}$ mice were randomly grouped into three groups, including high-fat diet (HFD) treated 8 weeks (n=6), 16 weeks (n=6), and AAV-sgIL-1β (n=6) mice. Data are presented as mean ± SE. Mann-Whitney test: *P<0.05.

2. original blots

Fig.7 a

1. oxLDL 0 μg/ml
2. oxLDL 10 μg/m
3. oxLDL 25 μg/m
4. oxLDL 50 μg/m
5. oxLDL 75 μg/m
6. oxLDL 100 μg/m
7. oxLDL 125 μg/ml
8. oxLDL 0 μg/ml
9. oxLDL 10 μg/m
10. oxLDL 25 μg/m
11. oxLDL 50 μg/m
12. oxLDL 75 μg/m
13. oxLDL 100 μg/m
14. oxLDL 125 μg/ml
15. oxLDL 0 μg/ml
16. oxLDL 10 μg/m
17. oxLDL 25 μg/m
18. oxLDL 50 μg/m
19. oxLDL 75 μg/m
20. oxLDL 100 μg/m
21. oxLDL 125 μg/ml
22. oxLDL 0 μg/ml
23. oxLDL 10 μg/m
24. oxLDL 25 μg/m
25. oxLDL 50 μg/m
26. oxLDL 75 μg/m
27. oxLDL 100 μg/m
28. oxLDL 125 μg/ml
29. oxLDL 0 μg/ml
30. oxLDL 10 μg/m
31. oxLDL 25 μg/m
32. oxLDL 50 μg/m
33. oxLDL 75 μg/m
34. oxLDL 100 μg/m
35. oxLDL 125 μg/ml
36. oxLDL 0 μg/ml
37. oxLDL 10 μg/m
38. oxLDL 25 μg/m
39. oxLDL 50 μg/m
40. oxLDL 75 μg/m
41. oxLDL 100 μg/m
42. oxLDL 125 μg/ml

IL-1β

GAPDH

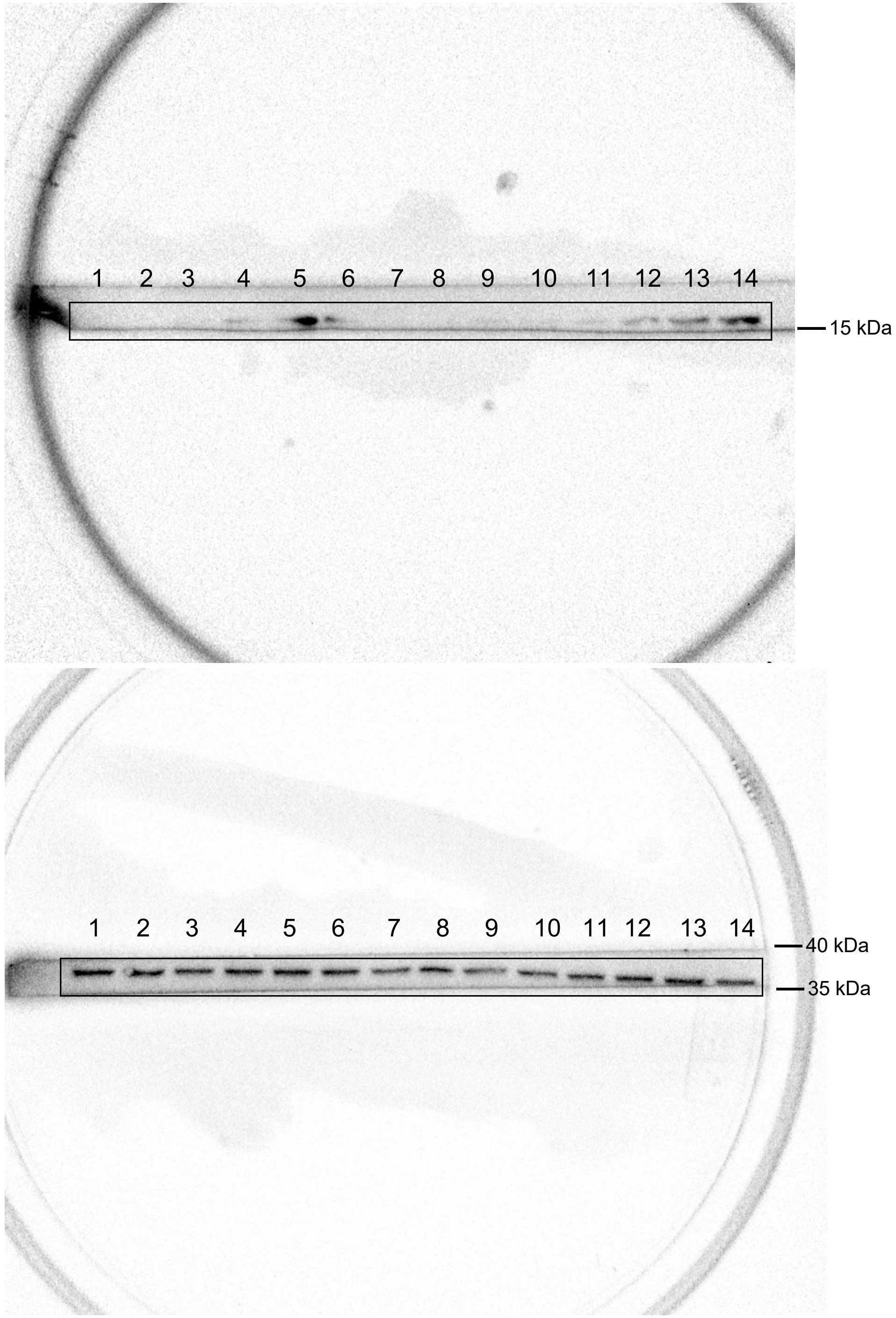

Fig.7 a

1. oxLDL 0 µg/ml
2. oxLDL 10 µg/m
3. oxLDL 25 µg/m
4. oxLDL 50 µg/m
5. oxLDL 75 µg/m
6. oxLDL 100 µg/m
7. oxLDL 125 µg/ml
8. oxLDL 0 µg/ml
9. oxLDL 10 µg/m
10. oxLDL 25 µg/m
11. oxLDL 50 µg/m
12. oxLDL 75 µg/m
13. oxLDL 100 µg/m
14. oxLDL 125 µg/ml
15. oxLDL 0 µg/ml
16. oxLDL 10 µg/m
17. oxLDL 25 µg/m
18. oxLDL 50 µg/m
19. oxLDL 75 µg/m
20. oxLDL 100 µg/m
21. oxLDL 125 µg/ml
22. oxLDL 0 µg/ml
23. oxLDL 10 µg/m
24. oxLDL 25 µg/m
25. oxLDL 50 µg/m
26. oxLDL 75 µg/m
27. oxLDL 100 µg/m
28. oxLDL 125 µg/ml
29. oxLDL 0 µg/ml
30. oxLDL 10 µg/m
31. oxLDL 25 µg/m
32. oxLDL 50 µg/m
33. oxLDL 75 µg/m
34. oxLDL 100 µg/m
35. oxLDL 125 µg/ml
36. oxLDL 0 µg/ml
37. oxLDL 10 µg/m
38. oxLDL 25 µg/m
39. oxLDL 50 µg/m
40. oxLDL 75 µg/m
41. oxLDL 100 µg/m
42. oxLDL 125 µg/ml

IL-1β

GAPDH

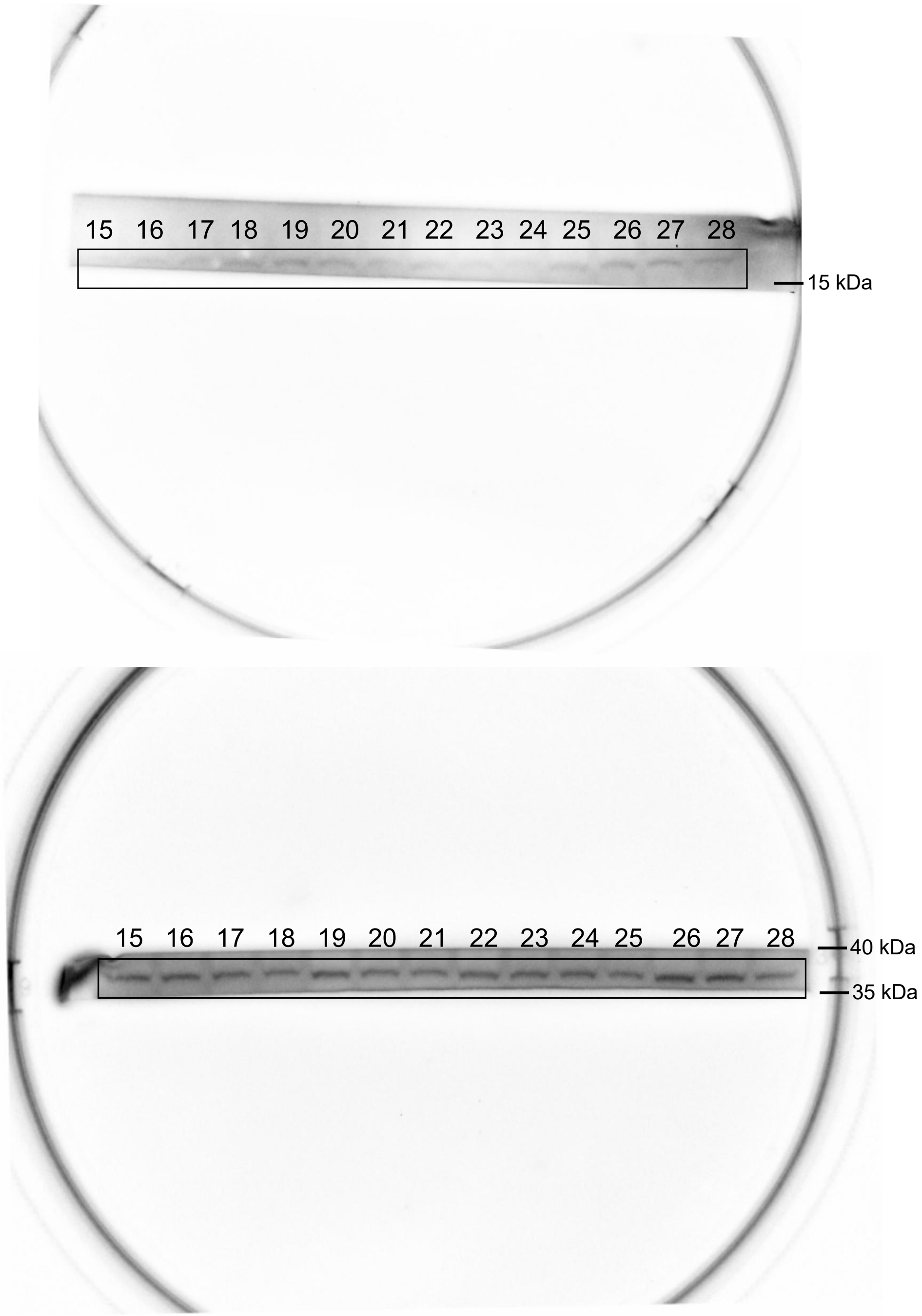

Fig.7 a

1. oxLDL 0 μg/ml
2. oxLDL 10 μg/m
3. oxLDL 25 μg/m
4. oxLDL 50 μg/m
5. oxLDL 75 μg/m
6. oxLDL 100 μg/m
7. oxLDL 125 μg/ml
8. oxLDL 0 μg/ml
9. oxLDL 10 μg/m
10. oxLDL 25 μg/m
11. oxLDL 50 μg/m
12. oxLDL 75 μg/m
13. oxLDL 100 μg/m
14. oxLDL 125 μg/ml
15. oxLDL 0 μg/ml
16. oxLDL 10 μg/m
17. oxLDL 25 μg/m
18. oxLDL 50 μg/m
19. oxLDL 75 μg/m
20. oxLDL 100 μg/m
21. oxLDL 125 μg/ml
22. oxLDL 0 μg/ml
23. oxLDL 10 μg/m
24. oxLDL 25 μg/m
25. oxLDL 50 μg/m
26. oxLDL 75 μg/m
27. oxLDL 100 μg/m
28. oxLDL 125 μg/ml
29. oxLDL 0 μg/ml
30. oxLDL 10 μg/m
31. oxLDL 25 μg/m
32. oxLDL 50 μg/m
33. oxLDL 75 μg/m
34. oxLDL 100 μg/m
35. oxLDL 125 μg/ml
36. oxLDL 0 μg/ml
37. oxLDL 10 μg/m
38. oxLDL 25 μg/m
39. oxLDL 50 μg/m
40. oxLDL 75 μg/m
41. oxLDL 100 μg/m
42. oxLDL 125 μg/ml

IL-1β

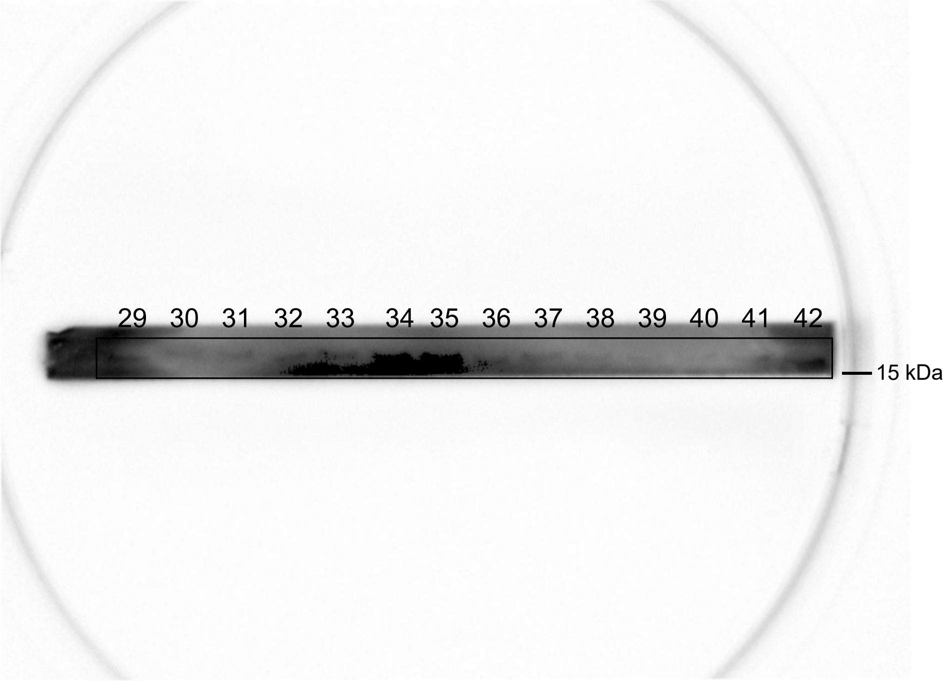

GAPDH

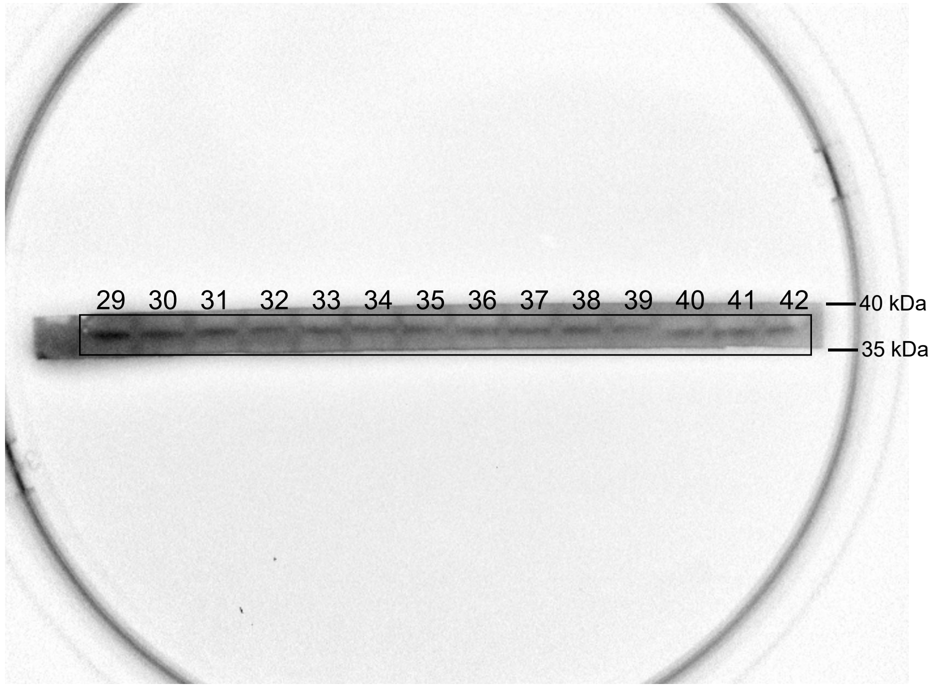

Fig.7 c

1. RM 0h
2. RM 12h
3. RM 24h
4. RM 48h
5. RM 12h
6. RM 24h
7. RM 48h
8. RM 0h
9. RM 12h
10. RM 24h
11. RM 48h
12. RM 12h
13. RM 24h
14. RM 48h
15. RM 0h
16. RM 12h
17. RM 24h
18. RM 48h
19. RM 12h
20. RM 24h
21. RM 48h
22. RM 0h
23. RM 12h
24. RM 24h
25. RM 48h
26. RM 12h
27. RM 24h
28. RM 48h

α-SMA

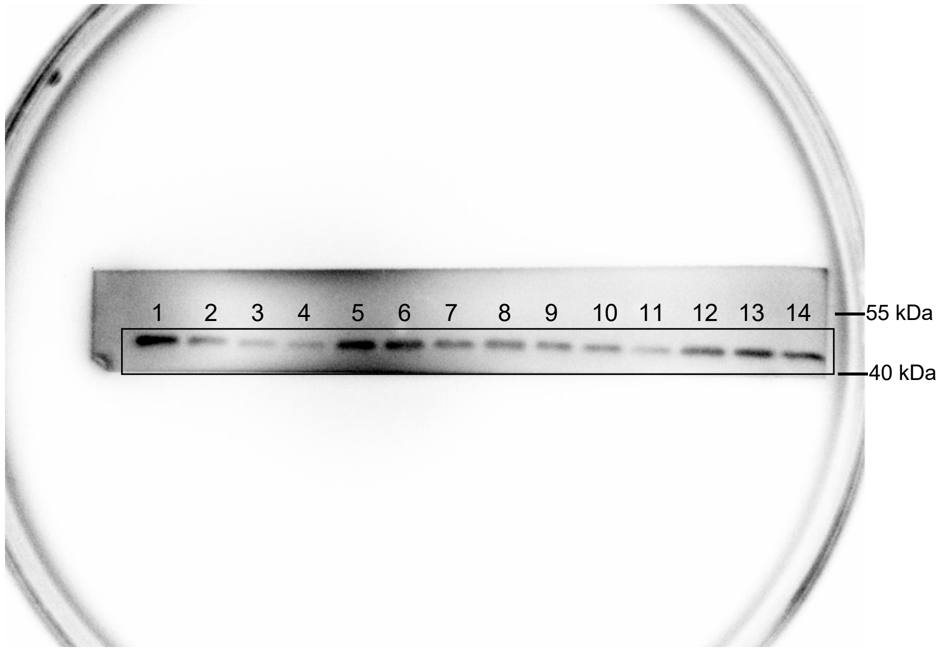

CD68

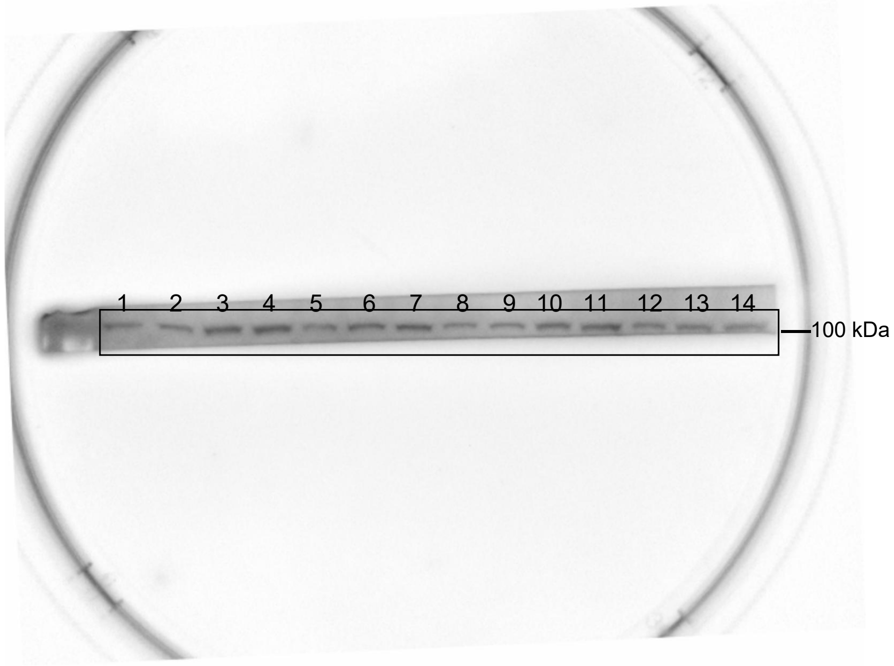

GAPDH

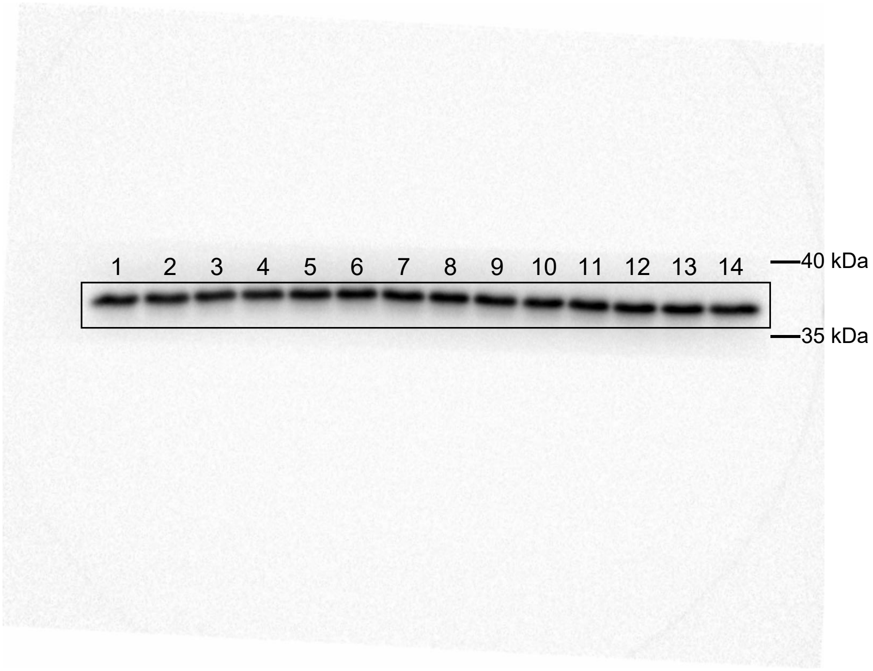

Fig.7 c

1. RM 0h
2. RM 12h
3. RM 24h
4. RM 48h
5. RM 12h
6. RM 24h
7. RM 48h
8. RM 0h
9. RM 12h
10. RM 24h
11. RM 48h
12. RM 12h
13. RM 24h
14. RM 48h
15. RM 0h
16. RM 12h
17. RM 24h
18. RM 48h
19. RM 12h
20. RM 24h
21. RM 48h
22. RM 0h
23. RM 12h
24. RM 24h
25. RM 48h
26. RM 12h
27. RM 24h
28. RM 48h

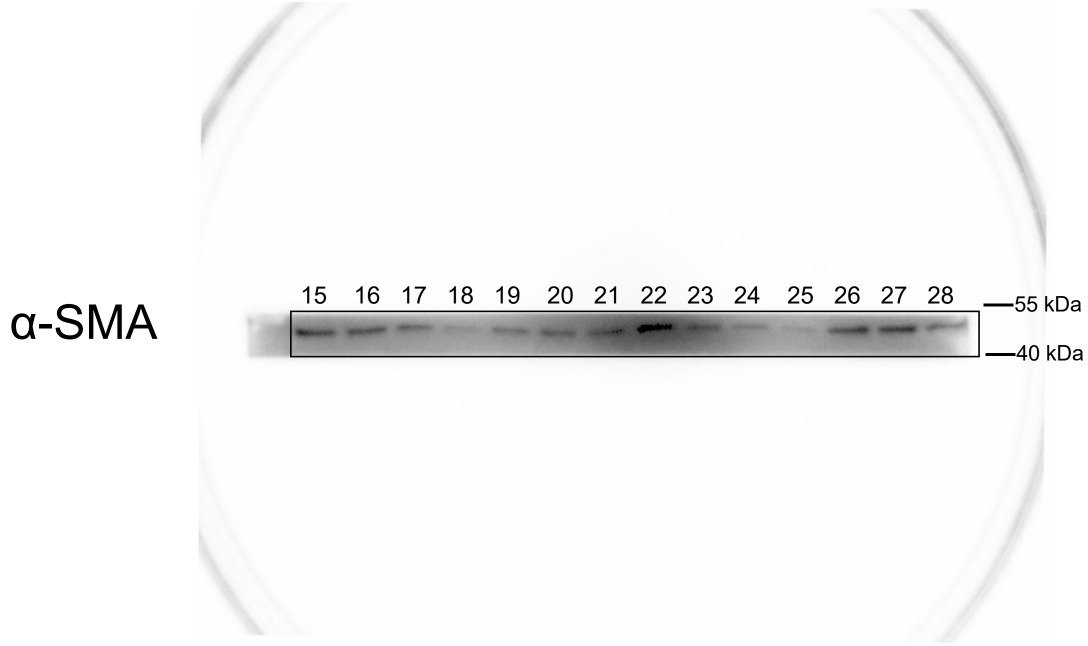

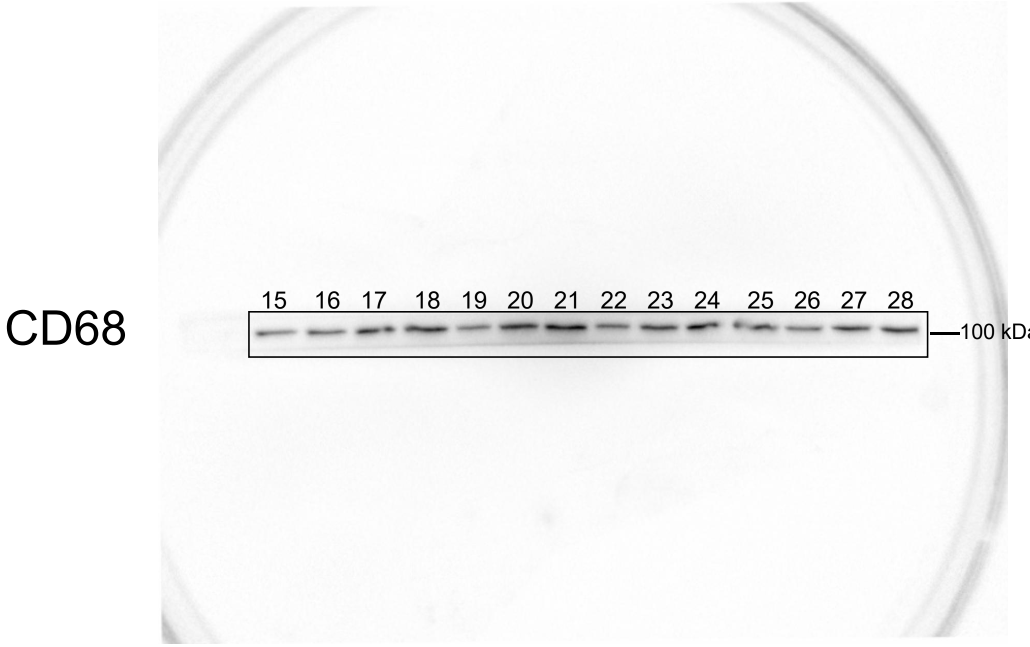

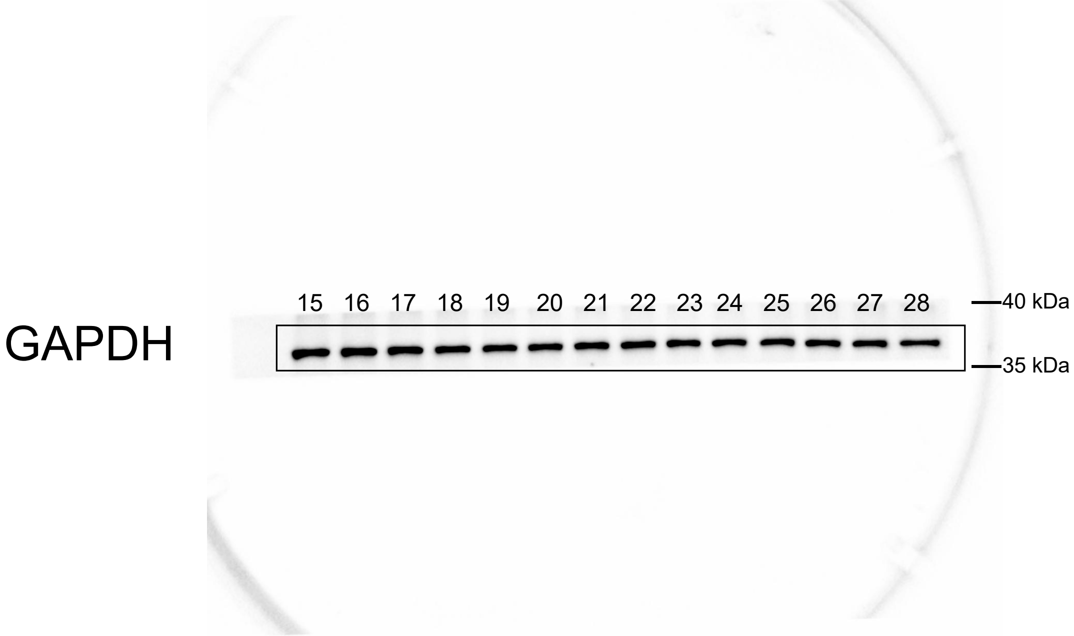

Fig.7 d

1. RM 0h
2. RM 12h
3. RM 24h
4. RM 48h
5. RM 12h
6. RM 24h
7. RM 48h
8. RM 0h
9. RM 12h
10. RM 24h
11. RM 48h
12. RM 12h
13. RM 24h
14. RM 48h
15. RM 0h
16. RM 12h
17. RM 24h
18. RM 48h
19. RM 12h
20. RM 24h
21. RM 48h
22. RM 0h
23. RM 12h
24. RM 24h
25. RM 48h
26. RM 12h
27. RM 24h
28. RM 48h

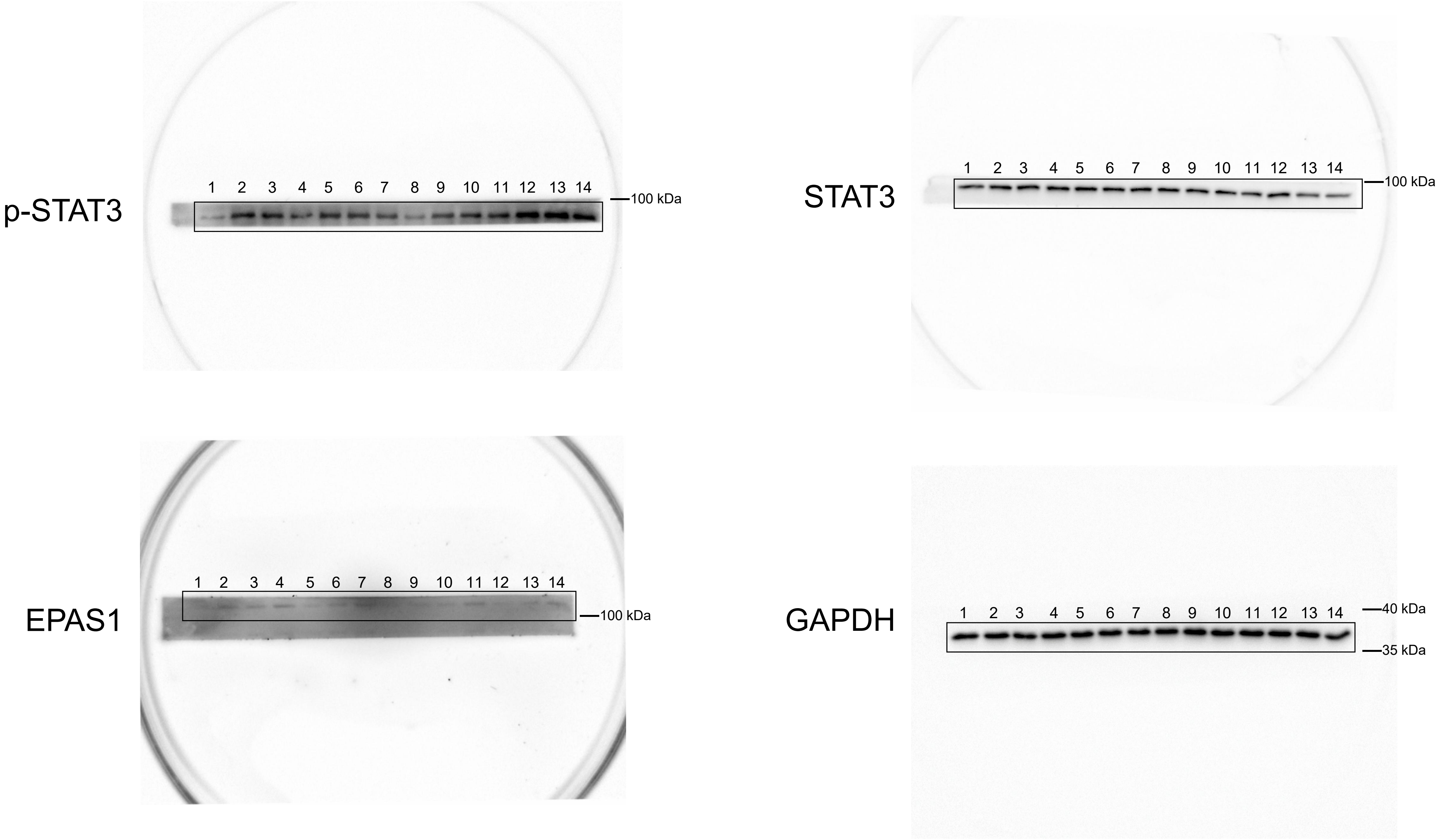

Fig.7 d

1. RM 0h
2. RM 12h
3. RM 24h
4. RM 48h
5. RM 12h
6. RM 24h
7. RM 48h
8. RM 0h
9. RM 12h
10. RM 24h
11. RM 48h
12. RM 12h
13. RM 24h
14. RM 48h
15. RM 0h
16. RM 12h
17. RM 24h
18. RM 48h
19. RM 12h
20. RM 24h
21. RM 48h
22. RM 0h
23. RM 12h
24. RM 24h
25. RM 48h
26. RM 12h
27. RM 24h
28. RM 48h

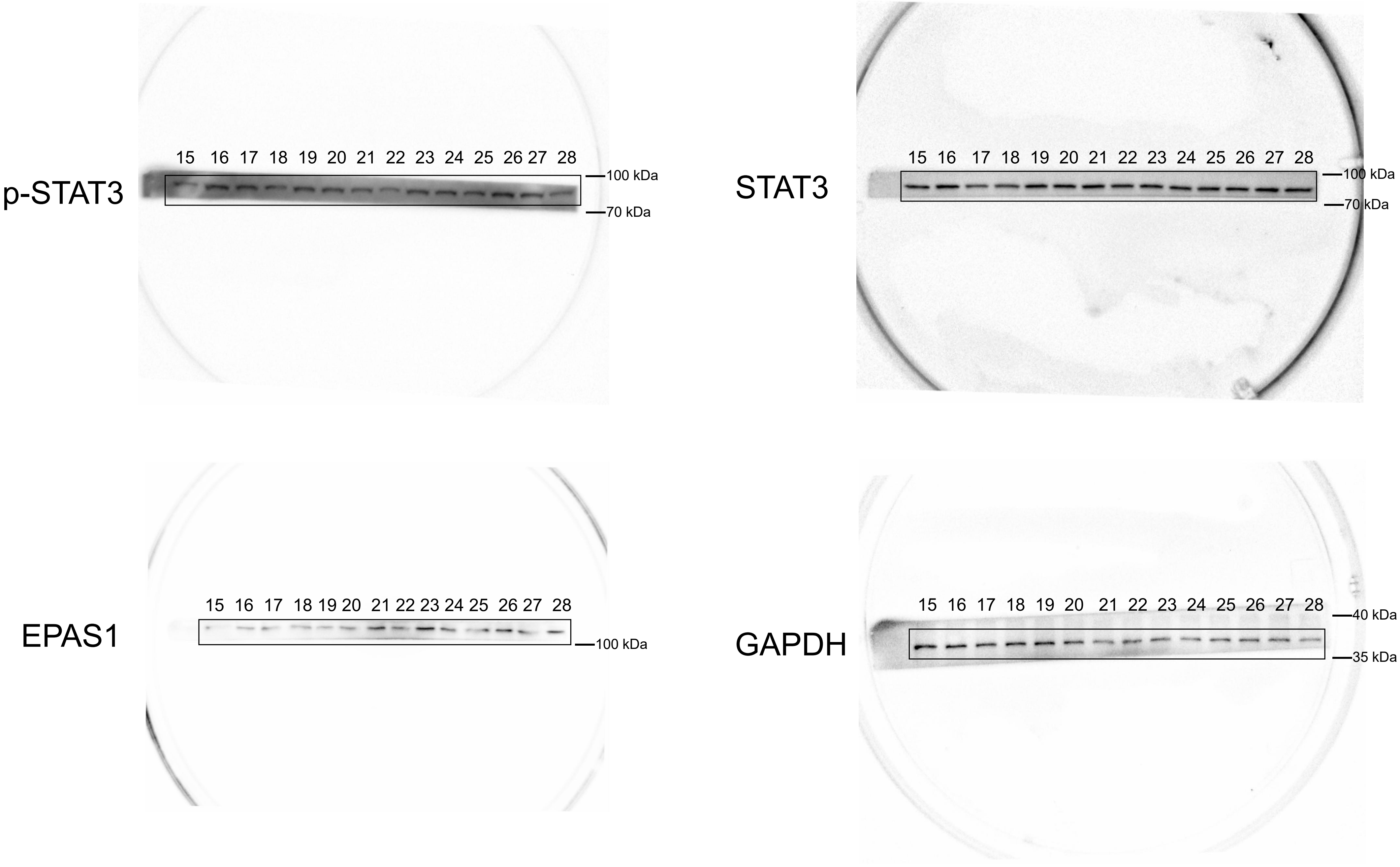

Fig.7 e

1. RM 12h
2. RM 48h
3. RM 12h+IL-1Ra
4. RM 48h+IL-1Ra
5. RM 12h
6. RM 28h
7. RM 12h+IL-1Ra
8. RM 48h+IL-1Ra
9. RM 12h
10. RM 48h
11. RM 12h+IL-1Ra
12. RM 48h+IL-1Ra
13. RM 12h
14. RM 48h
15. RM 12h+IL-1Ra
16. RM 48h+IL-1Ra
17. RM 12h
18. RM 48h
19. RM 12h+IL-1Ra
20. RM 48h+IL-1Ra
21. RM 12h
22. RM 48h
23. RM 12h+IL-1Ra
24. RM 48h+IL-1Ra

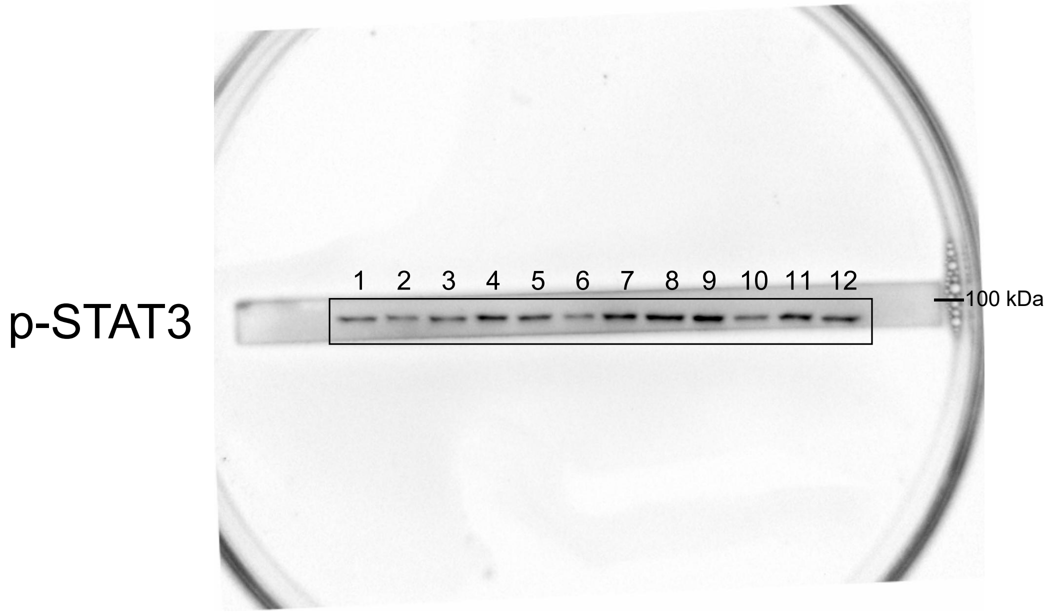

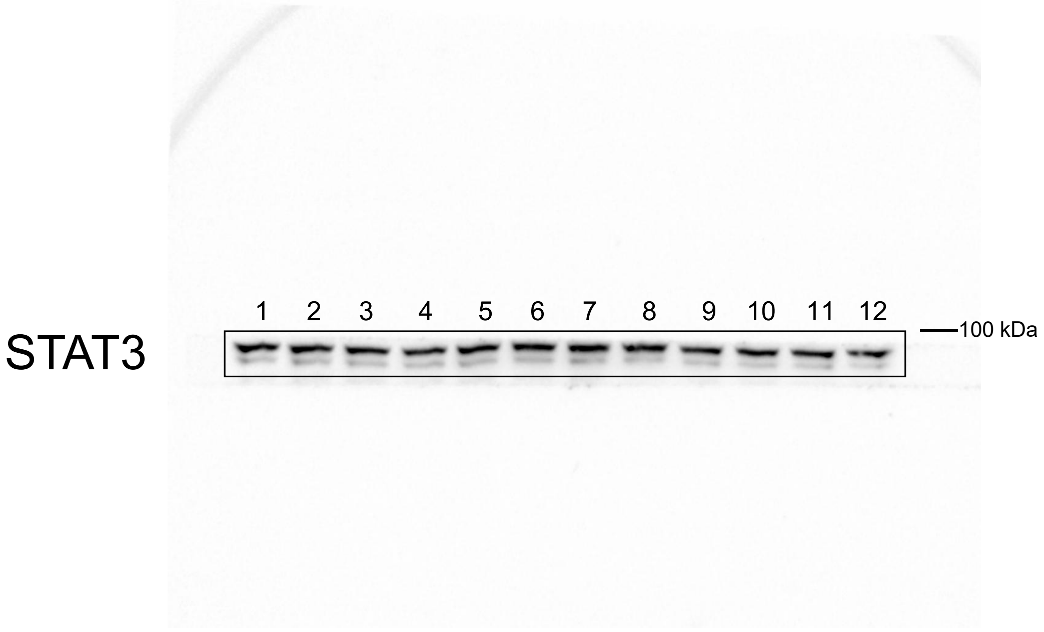

Fig.7 e

1. RM 12h
2. RM 48h
3. RM 12h+IL-1Ra
4. RM 48h+IL-1Ra
5. RM 12h
6. RM 28h
7. RM 12h+IL-1Ra
8. RM 48h+IL-1Ra
9. RM 12h
10. RM 48h
11. RM 12h+IL-1Ra
12. RM 48h+IL-1Ra
13. RM 12h
14. RM 48h
15. RM 12h+IL-1Ra
16. RM 48h+IL-1Ra
17. RM 12h
18. RM 48h
19. RM 12h+IL-1Ra
20. RM 48h+IL-1Ra
21. RM 12h
22. RM 48h
23. RM 12h+IL-1Ra
24. RM 48h+IL-1Ra

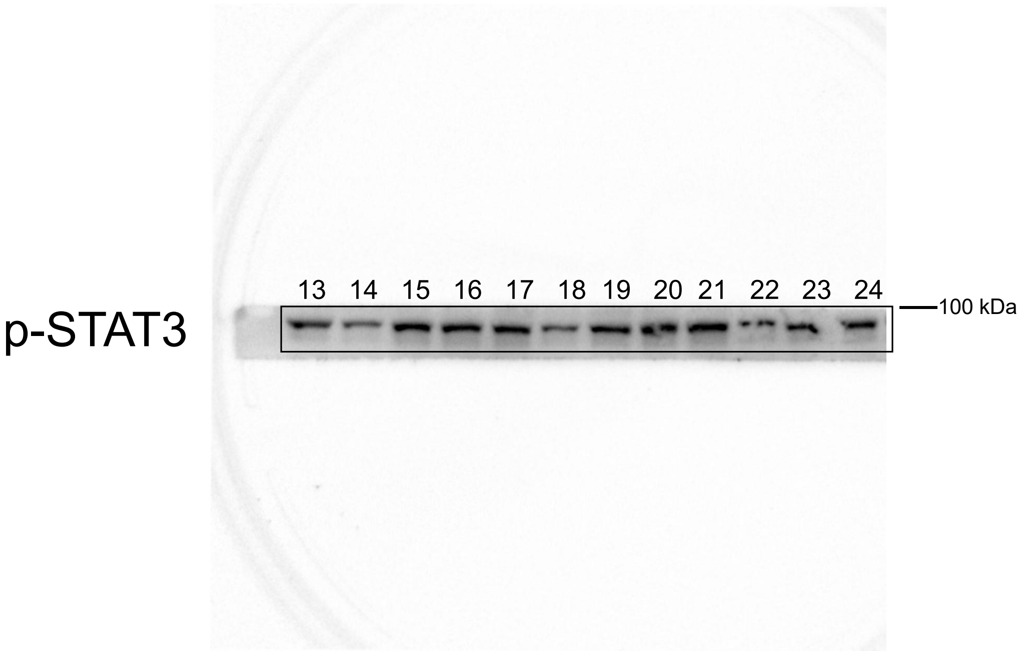

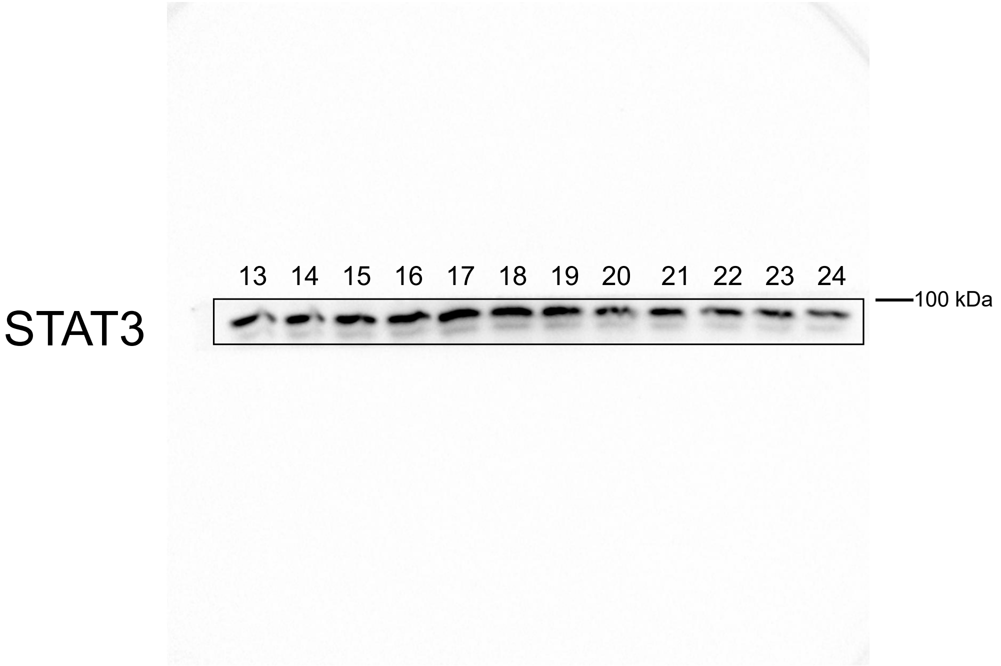

Fig.7 f

1. RM 12h
2. RM 48h
3. RM 48h+IL-1Ra
4. RM 48h+Colivelin
5. RM 48h+IL-1Ra+Stattic
6. RM 12h
7. RM 48h
8. RM 48h+IL-1Ra
9. RM 48h+Colivelin
10. RM 48h+IL-1Ra+Stattic

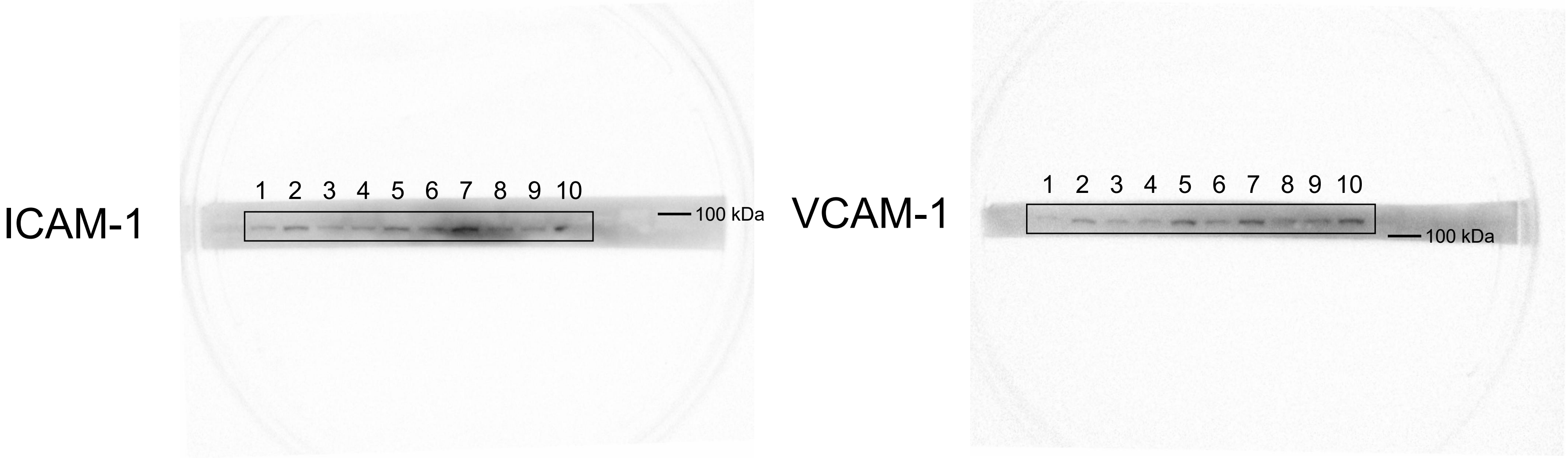

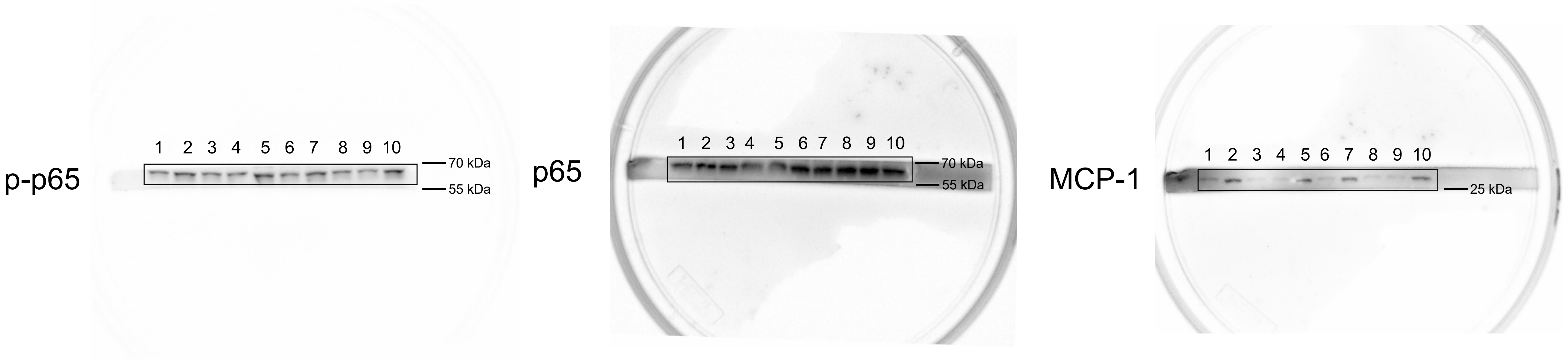

Fig.7 f

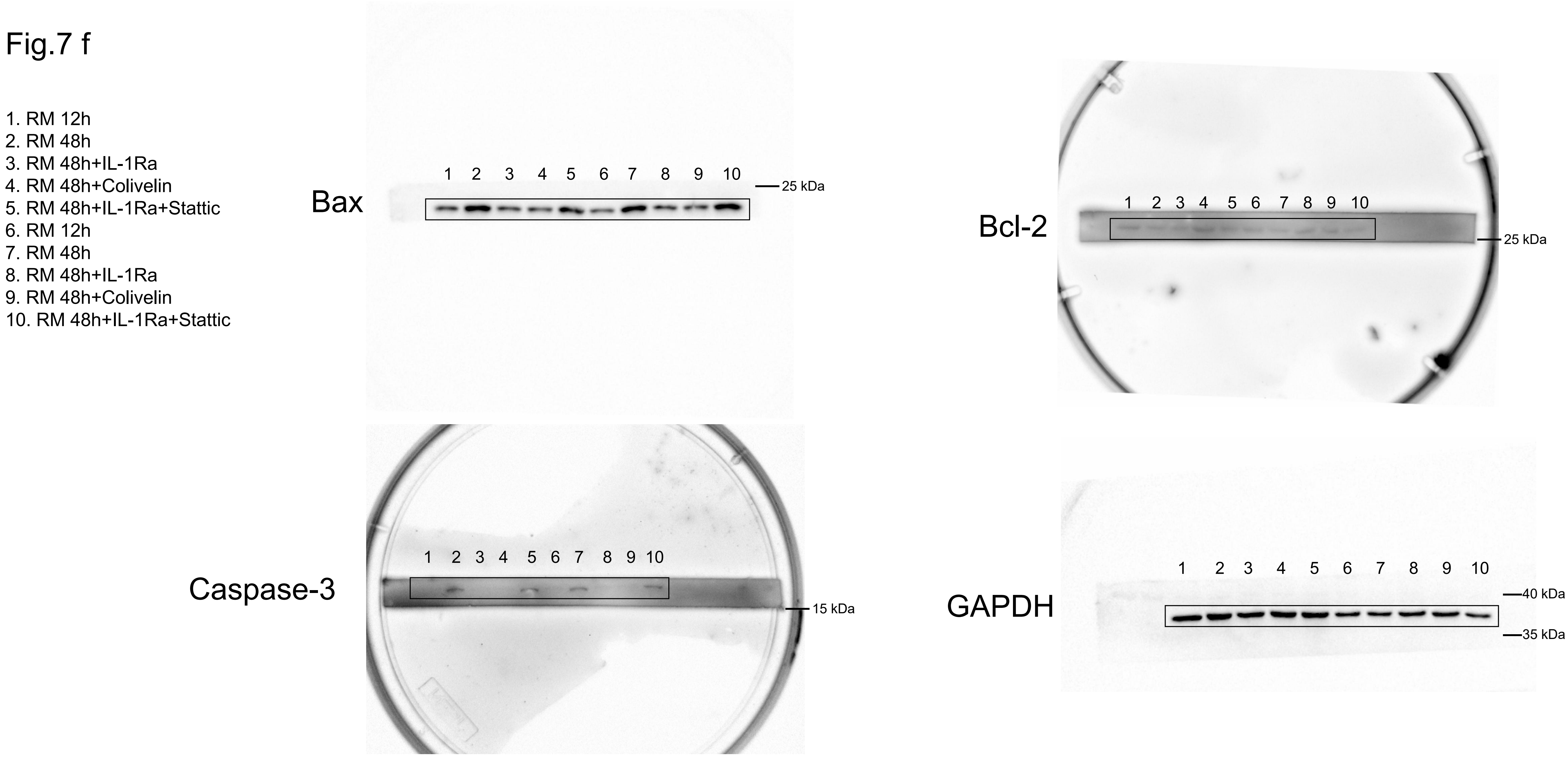
1. RM 12h
2. RM 48h
3. RM 48h+IL-1Ra
4. RM 48h+Colivelin
5. RM 48h+IL-1Ra+Stattic
6. RM 12h
7. RM 48h
8. RM 48h+IL-1Ra
9. RM 48h+Colivelin
10. RM 48h+IL-1Ra+Stattic
Bax
1 2 3 4 5 6 7 8 9 10
25 kDa
Bcl-2
1 2 3 4 5 6 7 8 9 10
25 kDa
Caspase-3
1 2 3 4 5 6 7 8 9 10
15 kDa
GAPDH
1 2 3 4 5 6 7 8 9 10
40 kDa
35 kDa

Fig.7 f

11. RM 12h
12. RM 48h
13. RM 48h+IL-1Ra
14. RM 48h+Colivelin
15. RM 48h+IL-1Ra+Stattic
16. RM 12h
17. RM 48h
18. RM 48h+IL-1Ra
19. RM 48h+Colivelin
20. RM 48h+IL-1Ra+Stattic

ICAM-1

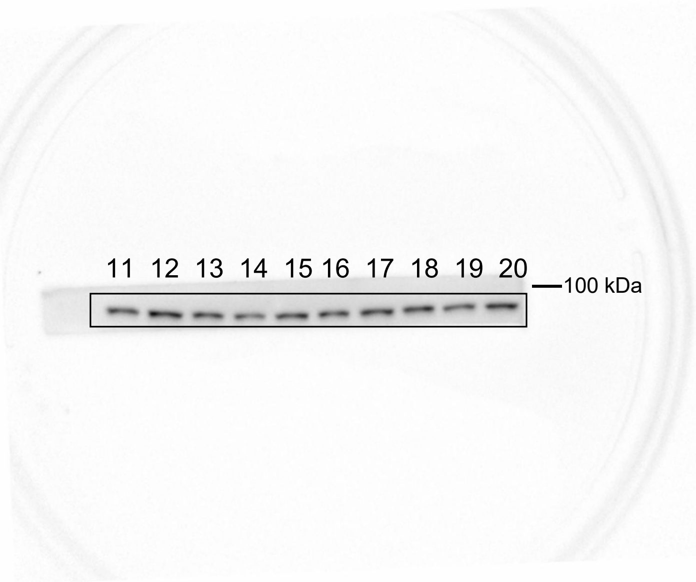

VCAM-1

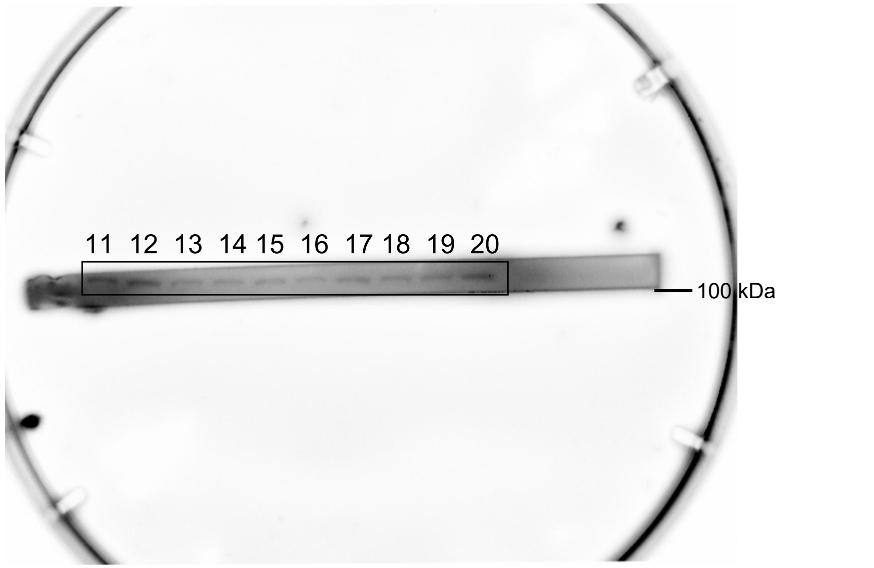

p-p65

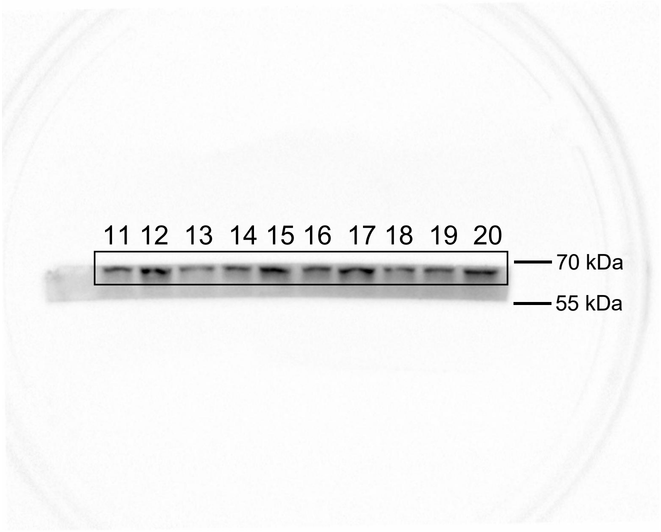

p65

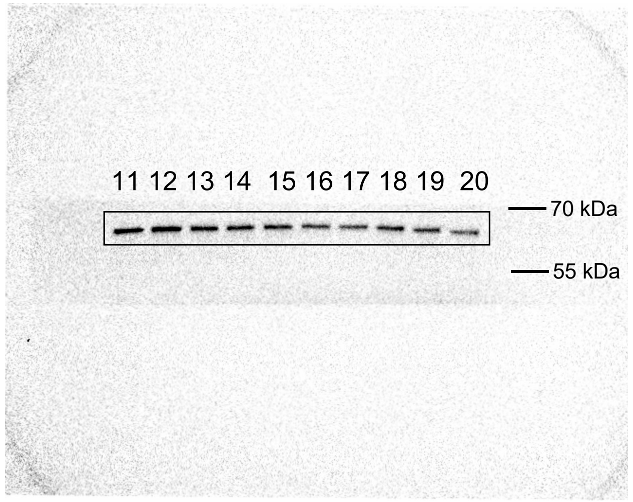

MCP-1

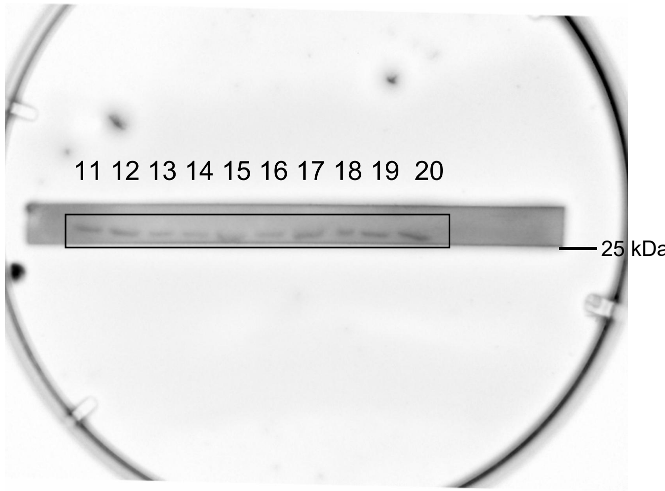

Fig.7 f

11. RM 12h
12. RM 48h
13. RM 48h+IL-1Ra
14. RM 48h+Colivelin
15. RM 48h+IL-1Ra+Stattic
16. RM 12h
17. RM 48h
18. RM 48h+IL-1Ra
19. RM 48h+Colivelin
20. RM 48h+IL-1Ra+Stattic

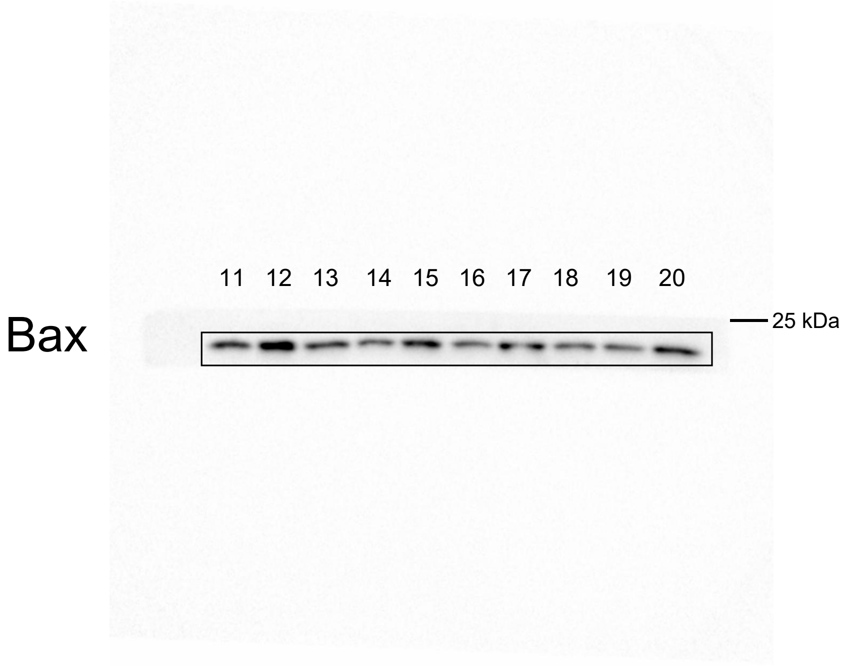

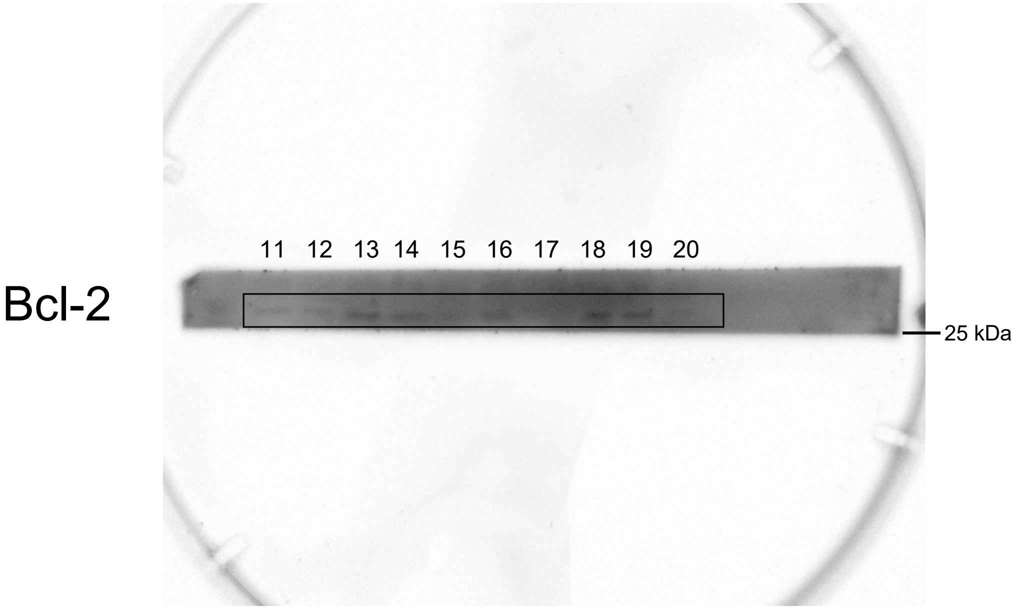

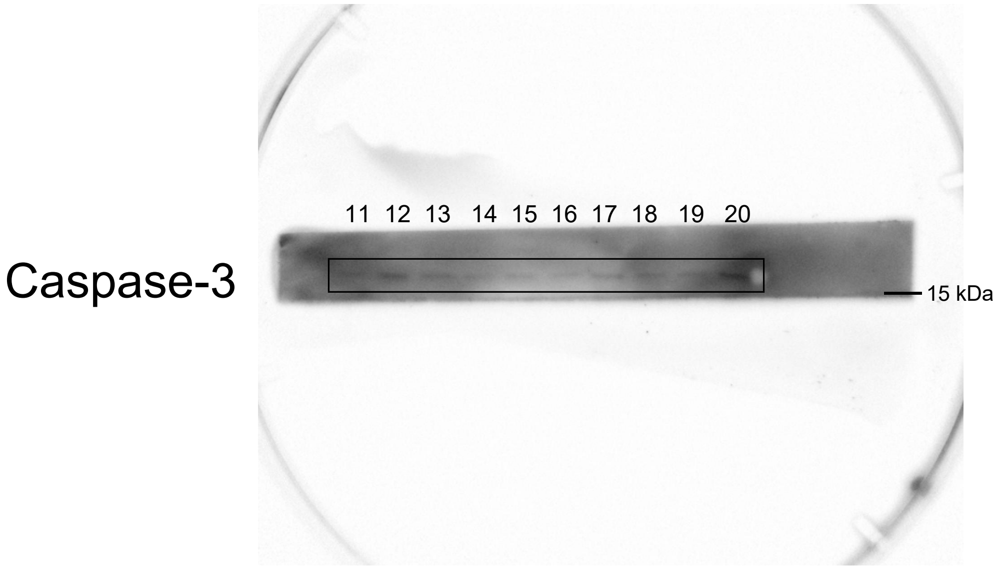

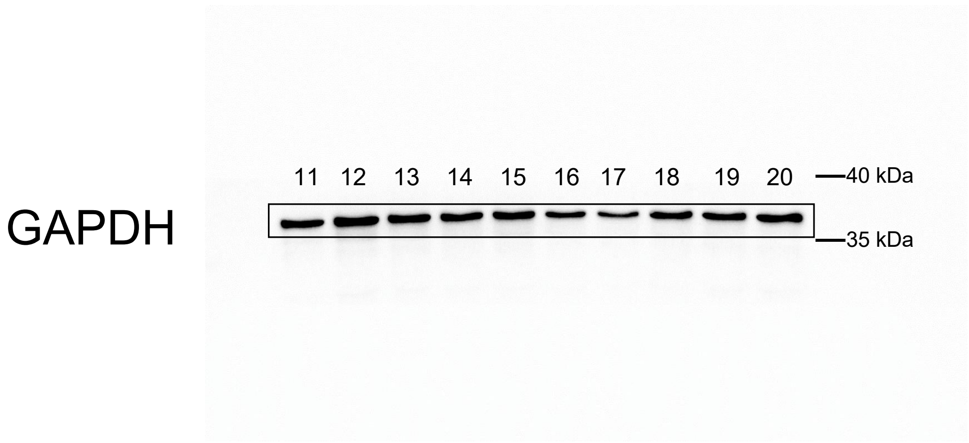

Fig.7 f

21. RM 12h
22. RM 48h
23. RM 48h+IL-1Ra
24. RM 48h+Colivelin
25. RM 48h+IL-1Ra+Stattic
26. RM 12h
27. RM 48h
28. RM 48h+IL-1Ra
29. RM 48h+Colivelin
30. RM 48h+IL-1Ra+Stattic

ICAM-1

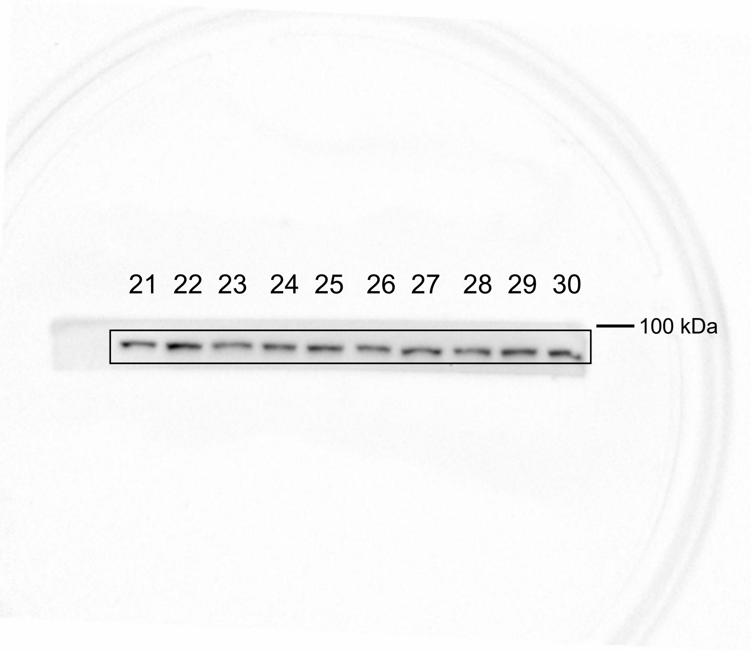

VCAM-1

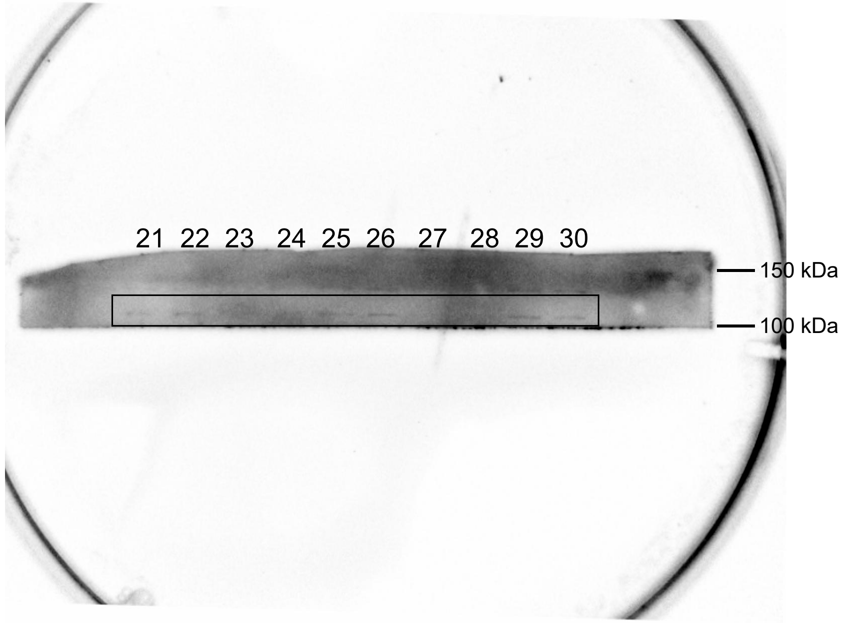

p-p65

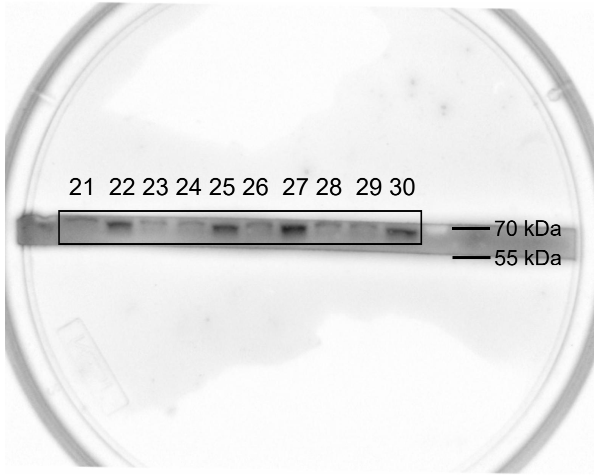

p65

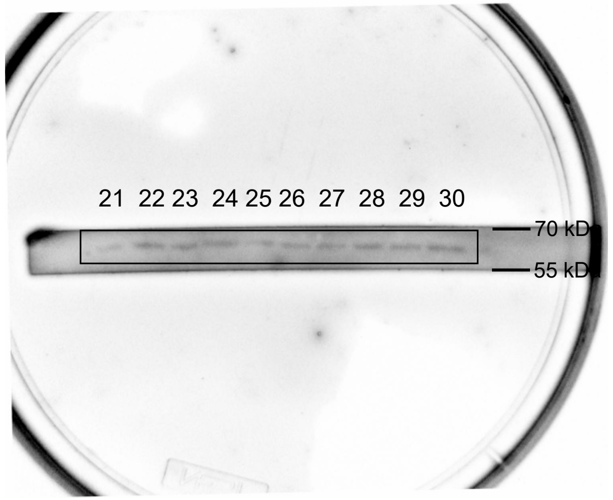

MCP-1

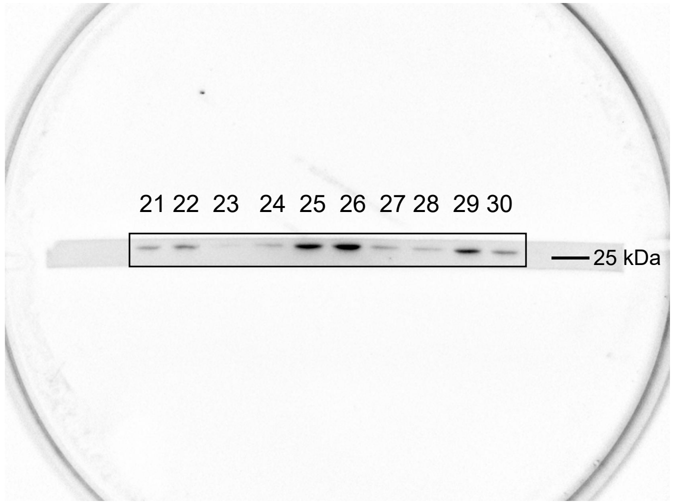

Fig.7 f

21. RM 12h
22. RM 48h
23. RM 48h+IL-1Ra
24. RM 48h+Colivelin
25. RM 48h+IL-1Ra+Stattic
26. RM 12h
27. RM 48h
28. RM 48h+IL-1Ra
29. RM 48h+Colivelin
30. RM 48h+IL-1Ra+Stattic

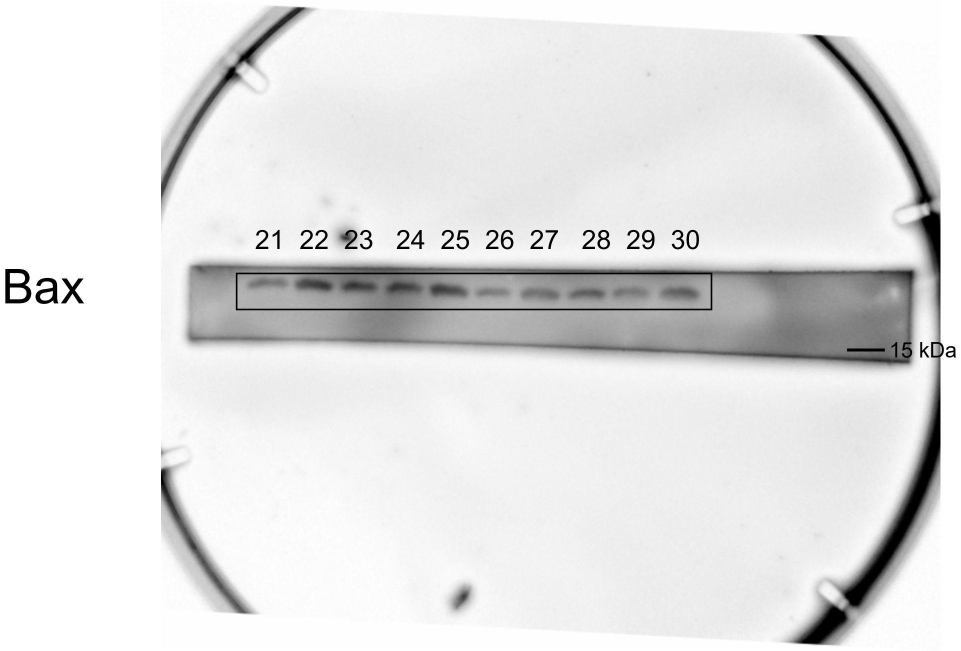

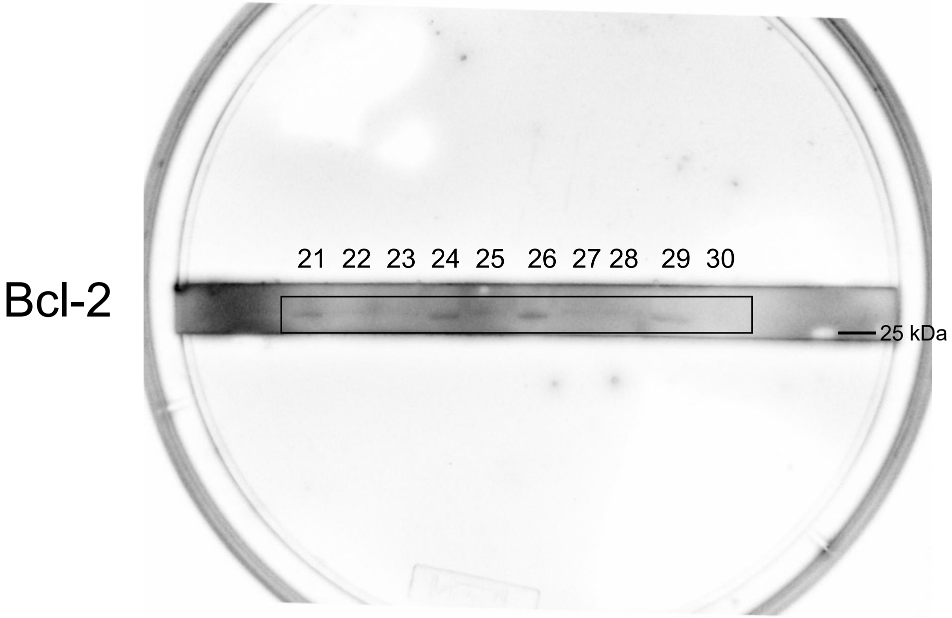

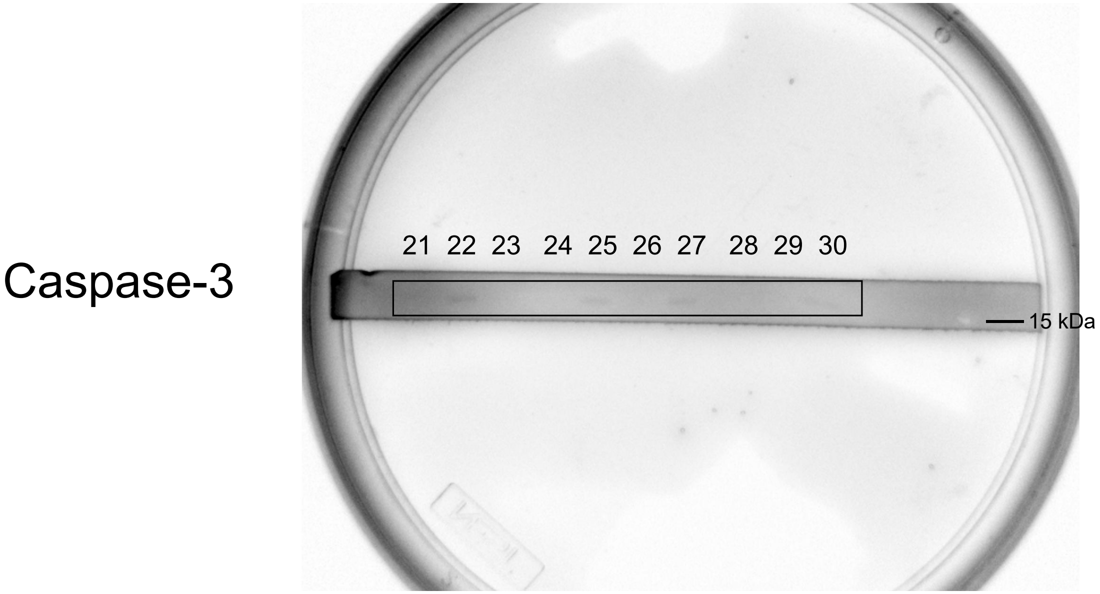

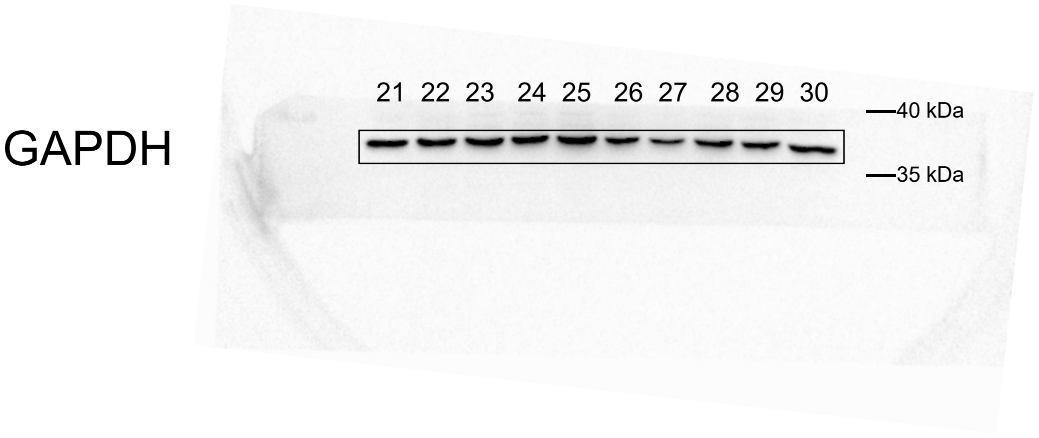

Fig.8 a

1. AS 8w
2. AS 16w AAV-NC
3. AS 16w AAV-sgIL-1β
4. AS 8w
5. AS 16w AAV-NC
6. AS 16w AAV-sgIL-1β
7. AS 8w
8. AS 16w AAV-NC
9. AS 16w AAV-sgIL-1β
10. AS 8w
11. AS 16w AAV-NC
12. AS 16w AAV-sgIL-1β
13. AS 8w
14. AS 16w AAV-NC
15. AS 16w AAV-IL-1β
16. AS 8w
17. AS 16w AAV-NC
18. AS 16w AAV-sgIL-1β

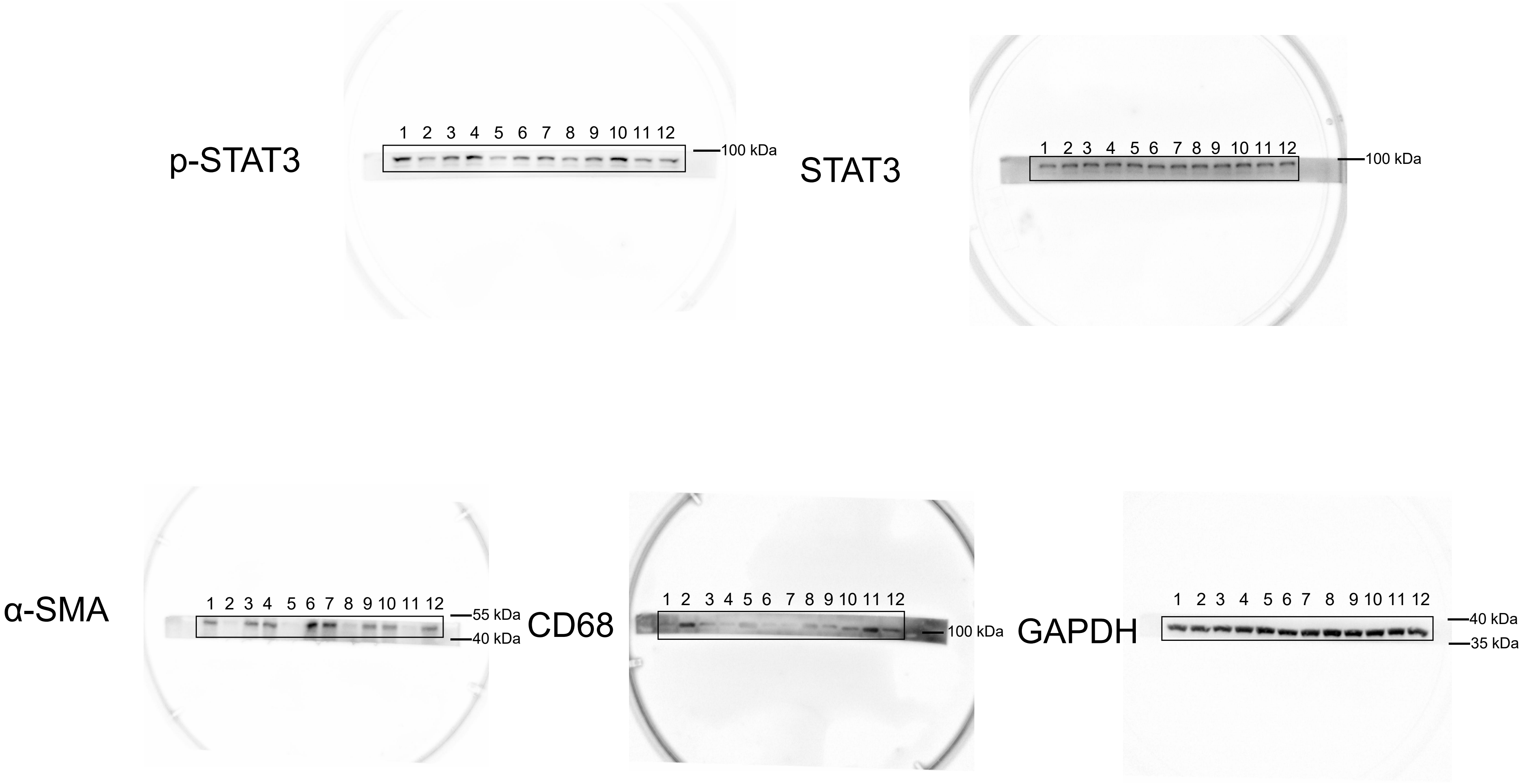

Fig.8 a

1. AS 8w
2. AS 16w AAV-NC
3. AS 16w AAV-sgIL-1β
4. AS 8w
5. AS 16w AAV-NC
6. AS 16w AAV-sgIL-1β
7. AS 8w
8. AS 16w AAV-NC
9. AS 16w AAV-sgIL-1β
10. AS 8w
11. AS 16w AAV-NC
12. AS 16w AAV-sgIL-1β
13. AS 8w
14. AS 16w AAV-NC
15. AS 16w AAV-IL-1β
16. AS 8w
17. AS 16w AAV-NC
18. AS 16w AAV-sgIL-1β

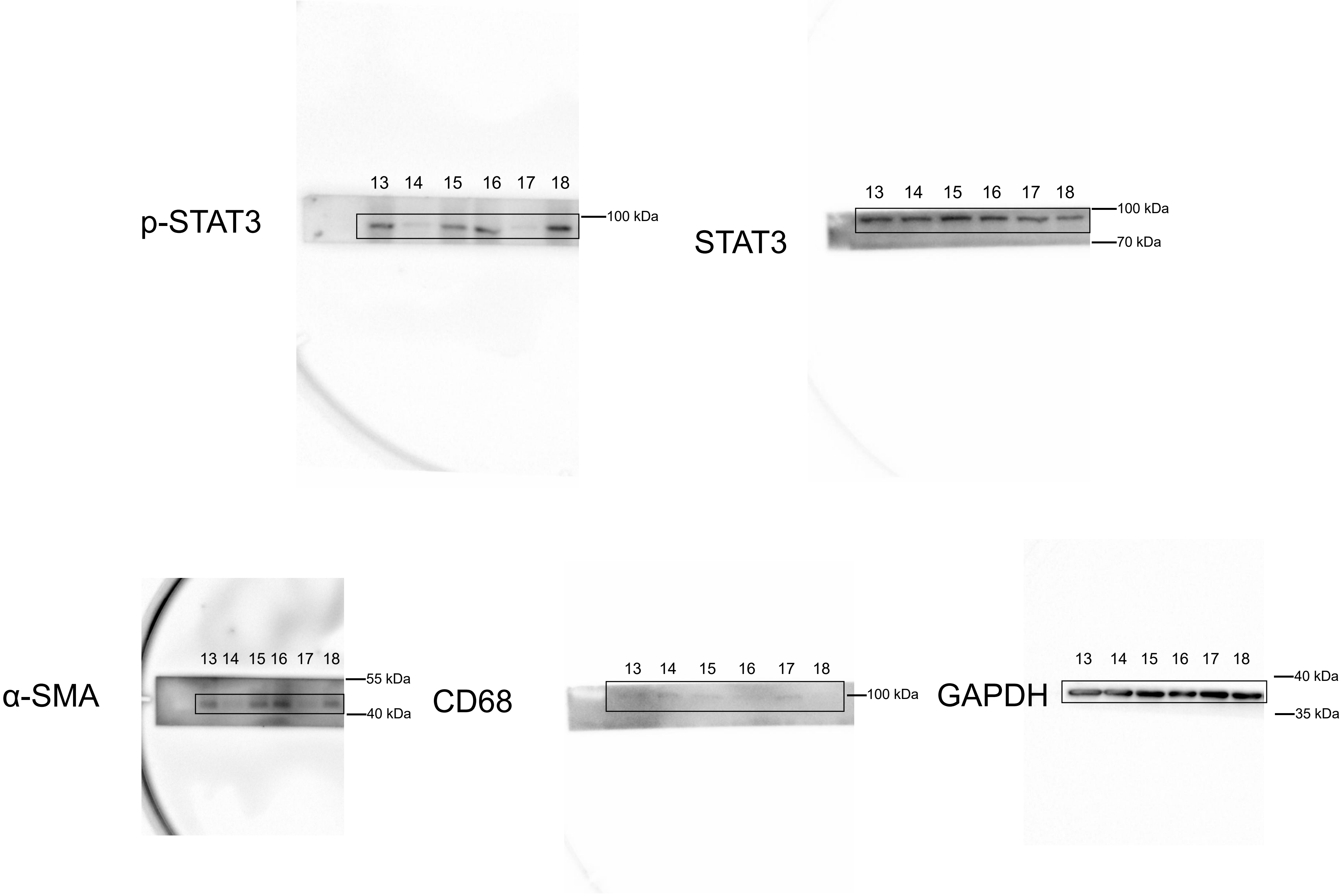

Sup Fig.9b

1. MM 12h
2. MM 48h
3. MM 12h+IL-1Ra
4. MM 48h+IL-1Ra
5. MM 12h
6. MM 48h
7. MM 12h+IL-1Ra
8. MM 48h+IL-1Ra
9. MM 12h
10. MM 48h
11. MM 12h+IL-1Ra
12. MM 48h+IL-1Ra
13. MM 12h
14. MM 48h
15. MM 12h+IL-1Ra
16. MM 48h+IL-1Ra
17. MM 12h
18. MM 48h
19. MM 12h+IL-1Ra
20. MM 48h+IL-1Ra
21. MM 12h
22. MM 48h
23. MM 12h+IL-1Ra
24. MM 48h+IL-1Ra

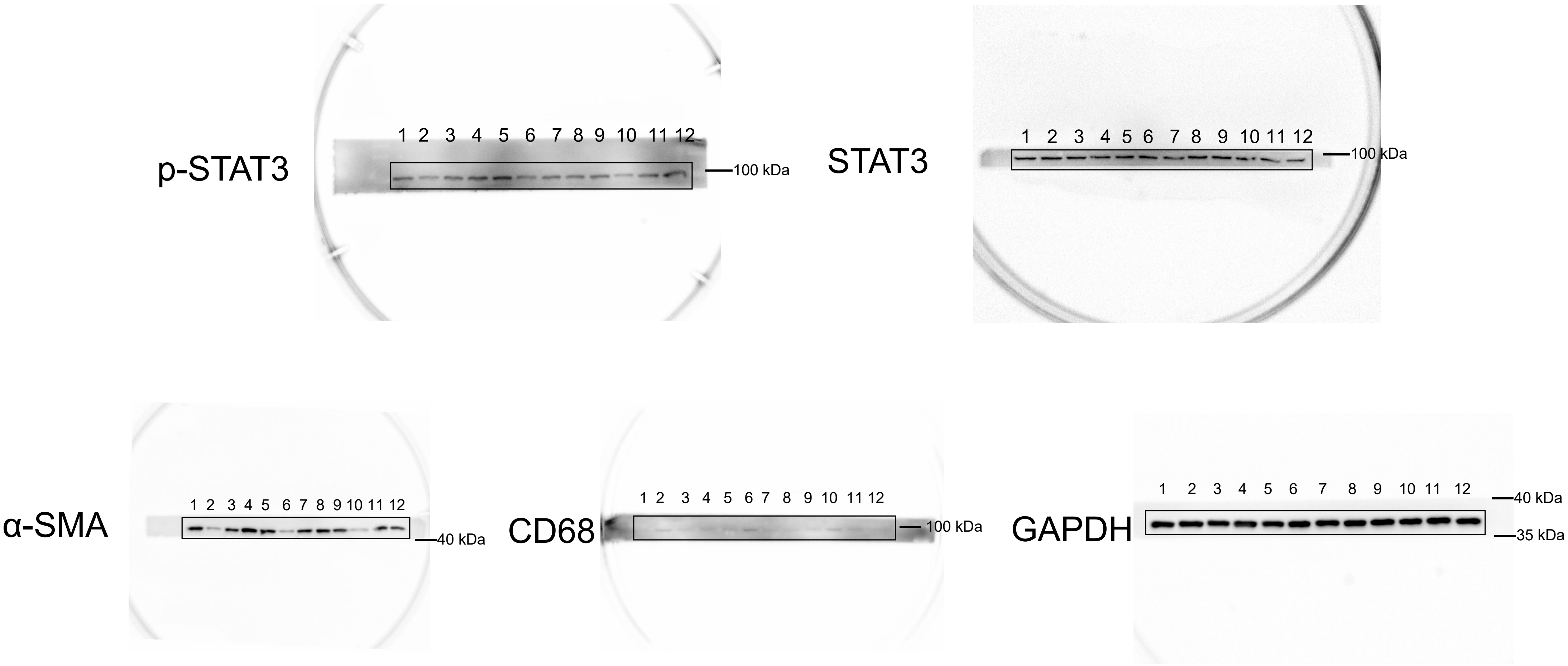

Sup Fig.9b

1. MM 12h
2. MM 48h
3. MM 12h+IL-1Ra
4. MM 48h+IL-1Ra
5. MM 12h
6. MM 48h
7. MM 12h+IL-1Ra
8. MM 48h+IL-1Ra
9. MM 12h
10. MM 48h
11. MM 12h+IL-1Ra
12. MM 48h+IL-1Ra
13. MM 12h
14. MM 48h
15. MM 12h+IL-1Ra
16. MM 48h+IL-1Ra
17. MM 12h
18. MM 48h
19. MM 12h+IL-1Ra
20. MM 48h+IL-1Ra
21. MM 12h
22. MM 48h
23. MM 12h+IL-1Ra
24. MM 48h+IL-1Ra

p-STAT3

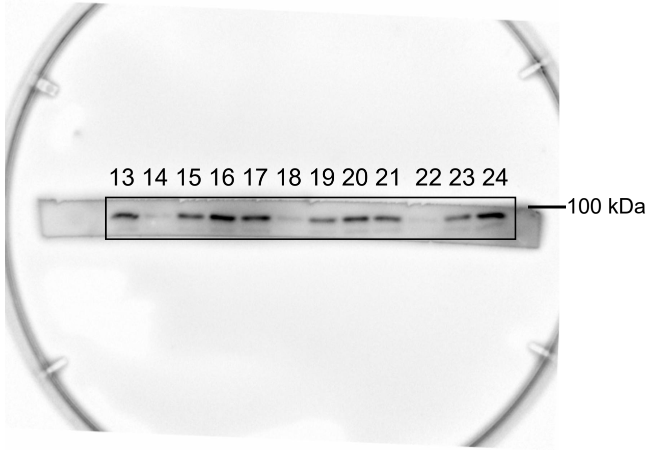

STAT3

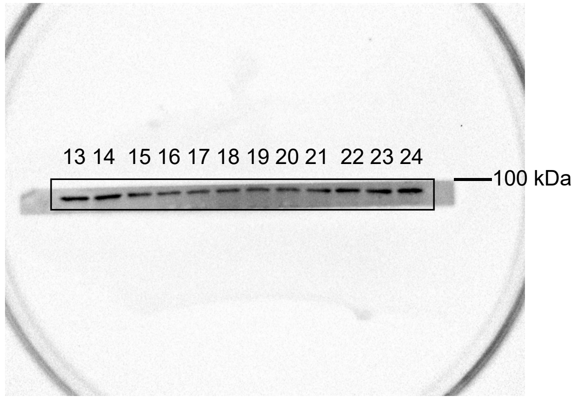

α-SMA

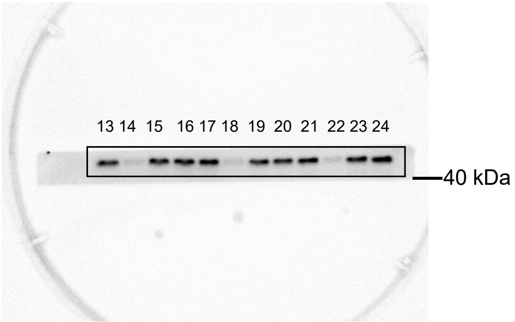

CD68

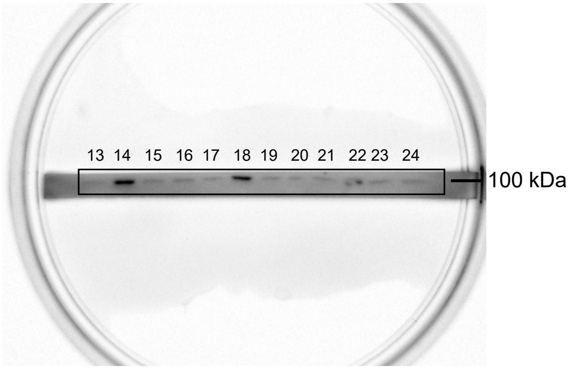

GAPDH

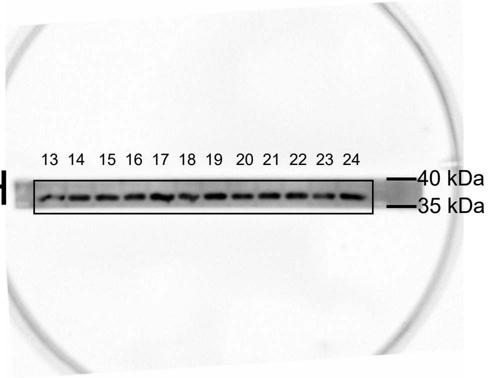

Sup Fig.9e

1. MM 12h
2. MM 48h
3. MM 12h+IL-1Ra
4. MM 48h+IL-1Ra
5. MM 12h
6. MM 48h
7. MM 12h+IL-1Ra
8. MM 48h+IL-1Ra
9. MM 12h
10. MM 48h
11. MM 12h+IL-1Ra
12. MM 48h+IL-1Ra
13. MM 12h
14. MM 48h
15. MM 12h+IL-1Ra
16. MM 48h+IL-1Ra
17. MM 12h
18. MM 48h
19. MM 12h+IL-1Ra
20. MM 48h+IL-1Ra
21. MM 12h
22. MM 48h
23. MM 12h+IL-1Ra
24. MM 48h+IL-1Ra

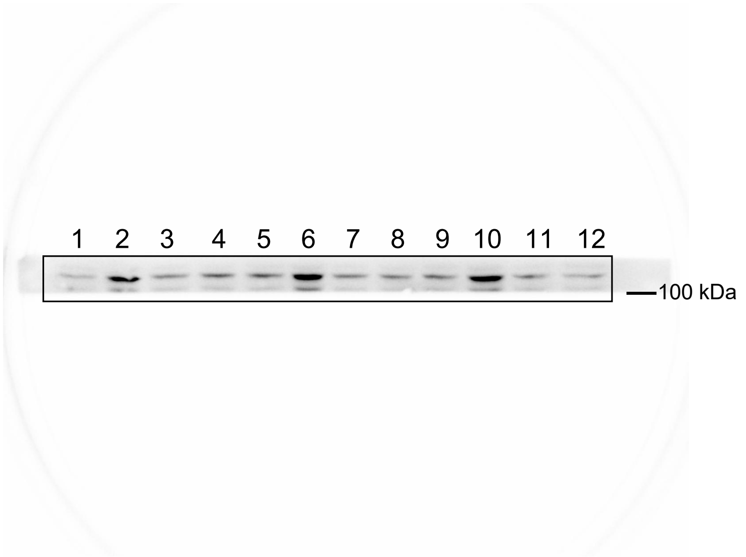

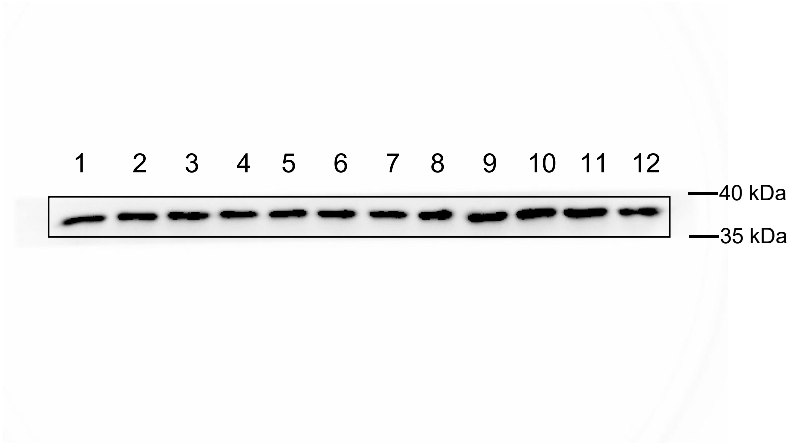

Sup Fig.9e

1. MM 12h
2. MM 48h
3. MM 12h+IL-1Ra
4. MM 48h+IL-1Ra
5. MM 12h
6. MM 48h
7. MM 12h+IL-1Ra
8. MM 48h+IL-1Ra
9. MM 12h
10. MM 48h
11. MM 12h+IL-1Ra
12. MM 48h+IL-1Ra
13. MM 12h
14. MM 48h
15. MM 12h+IL-1Ra
16. MM 48h+IL-1Ra
17. MM 12h
18. MM 48h
19. MM 12h+IL-1Ra
20. MM 48h+IL-1Ra
21. MM 12h
22. MM 48h
23. MM 12h+IL-1Ra
24. MM 48h+IL-1Ra

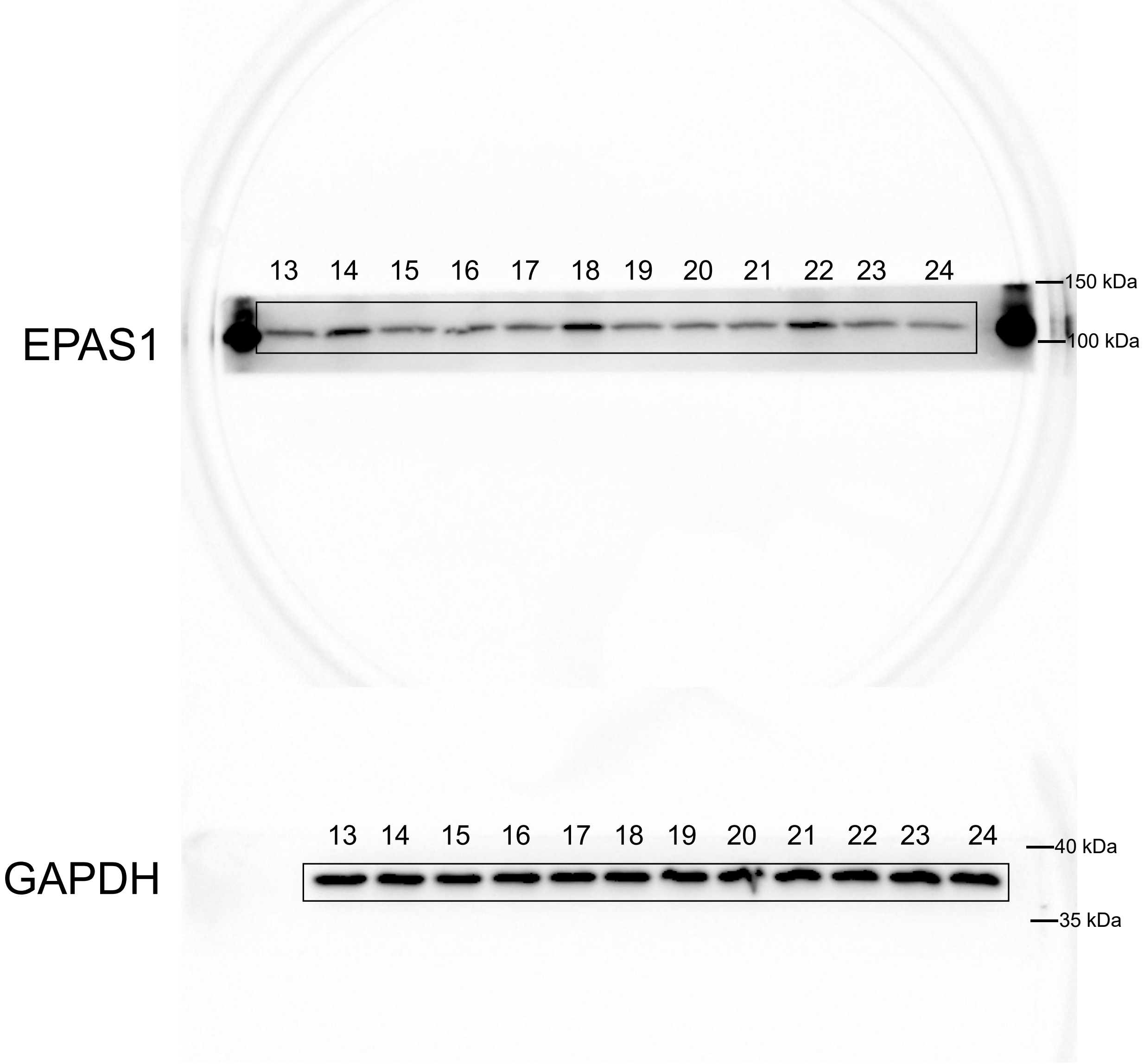

Sup Fig.11a

1. AS 8w
2. AS 16w AAV-NC
3. AS 16w AAV-sgIL-1β
4. AS 8w
5. AS 16w AAV-NC
6. AS 16w AAV-sgIL-1β
7. AS 8w
8. AS 16w AAV-NC
9. AS 16w AAV-sgIL-1β
10. AS 8w
11. AS 16w AAV-NC
12. AS 16w AAV-sgIL-1β
13. AS 8w
14. AS 16w AAV-NC
15. AS 16w AAV-sgIL-1β
16. AS 8w
17. AS 16w AAV-NC
18. AS 16w AAV-sgIL-1β

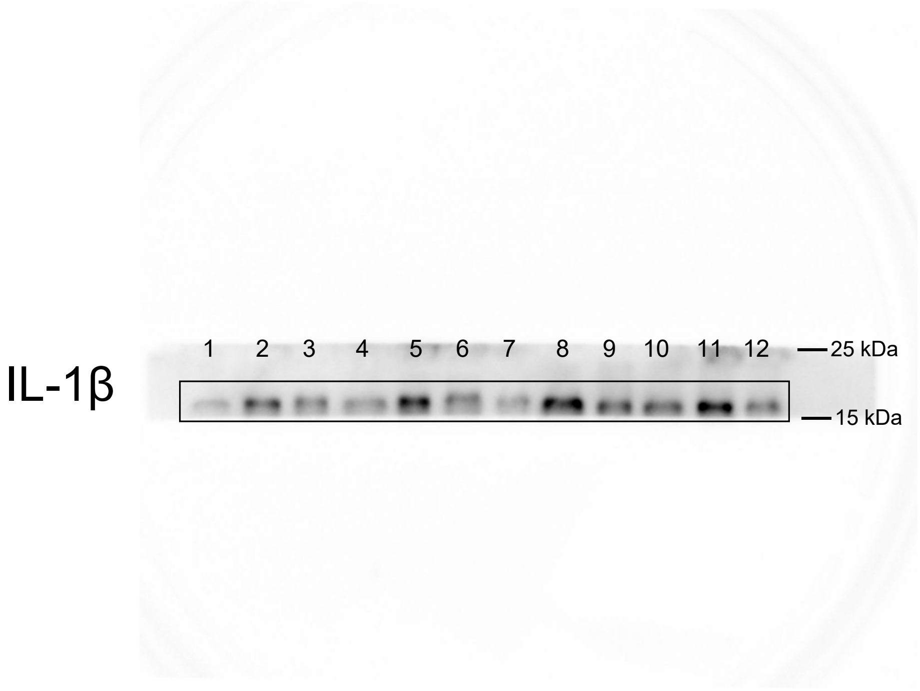

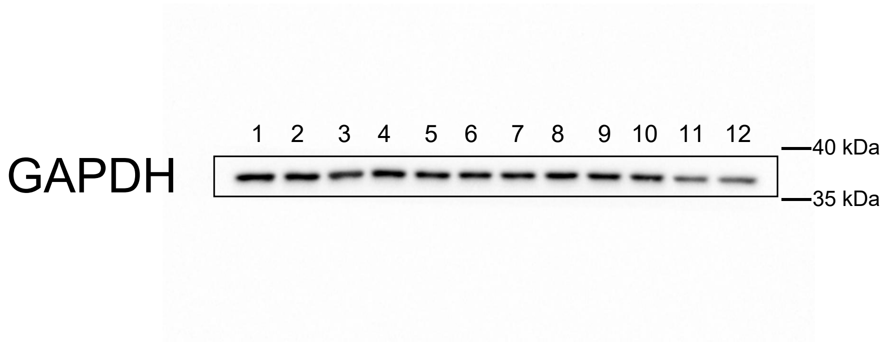

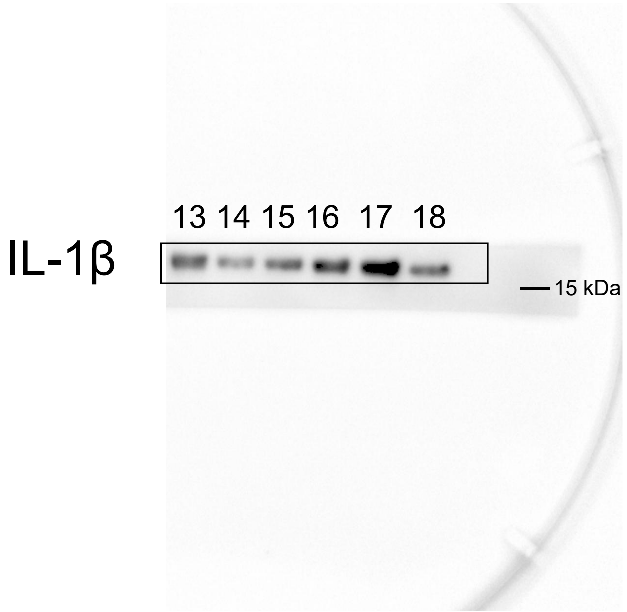

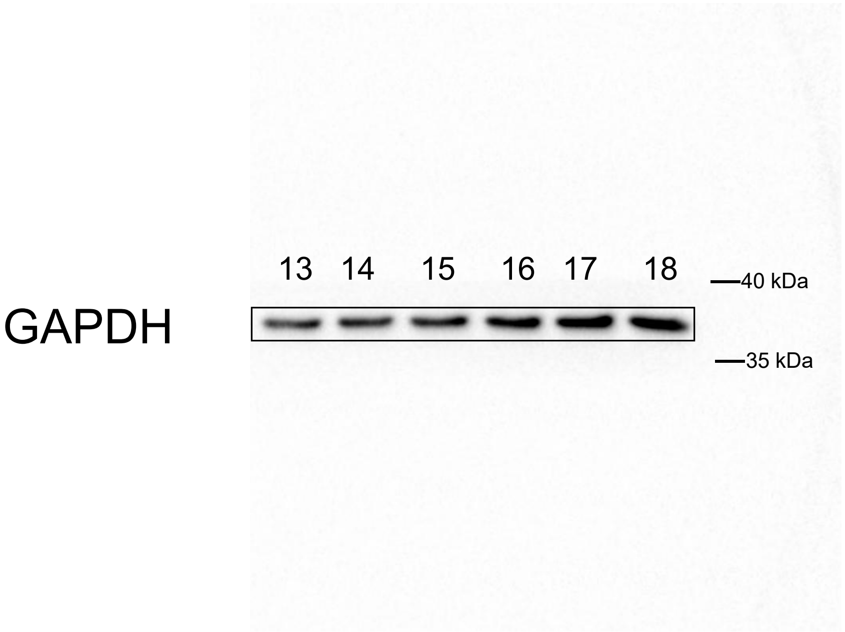

Sup Fig.11c

1. AS 8w
2. AS 16w AAV-NC
3. AS 16w AAV-sgIL-1β
4. AS 8w
5. AS 16w AAV-NC
6. AS 16w AAV-sgIL-1β
7. AS 8w
8. AS 16w AAV-NC
9. AS 16w AAV-sgIL-1β
10. AS 8w
11. AS 16w AAV-NC
12. AS 16w AAV-sgIL-1β
13. AS 8w
14. AS 16w AAV-NC
15. AS 16w AAV-sgIL-1β
16. AS 8w
17. AS 16w AAV-NC
18. AS 16w AAV-sgIL-1β

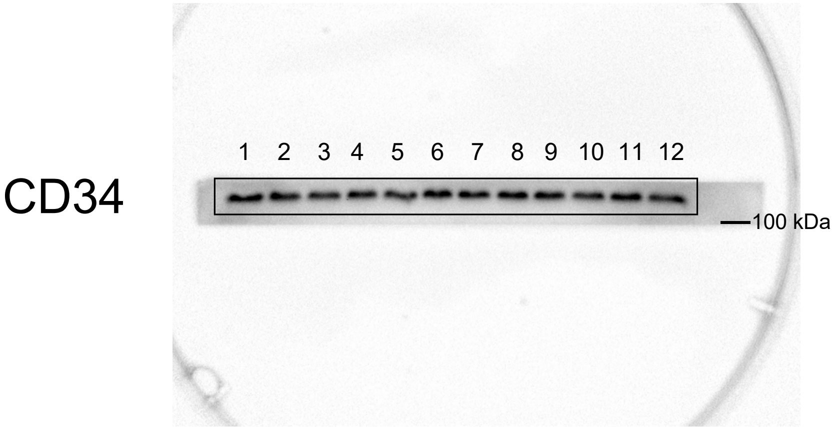

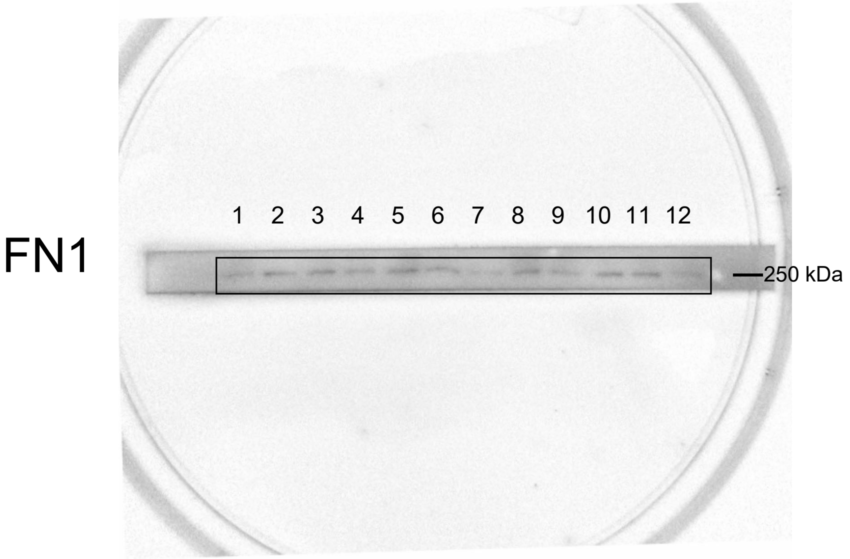

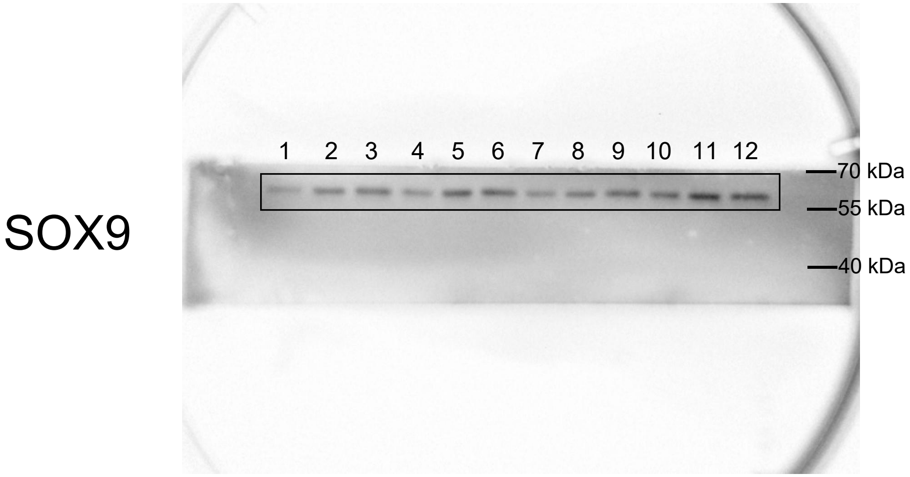

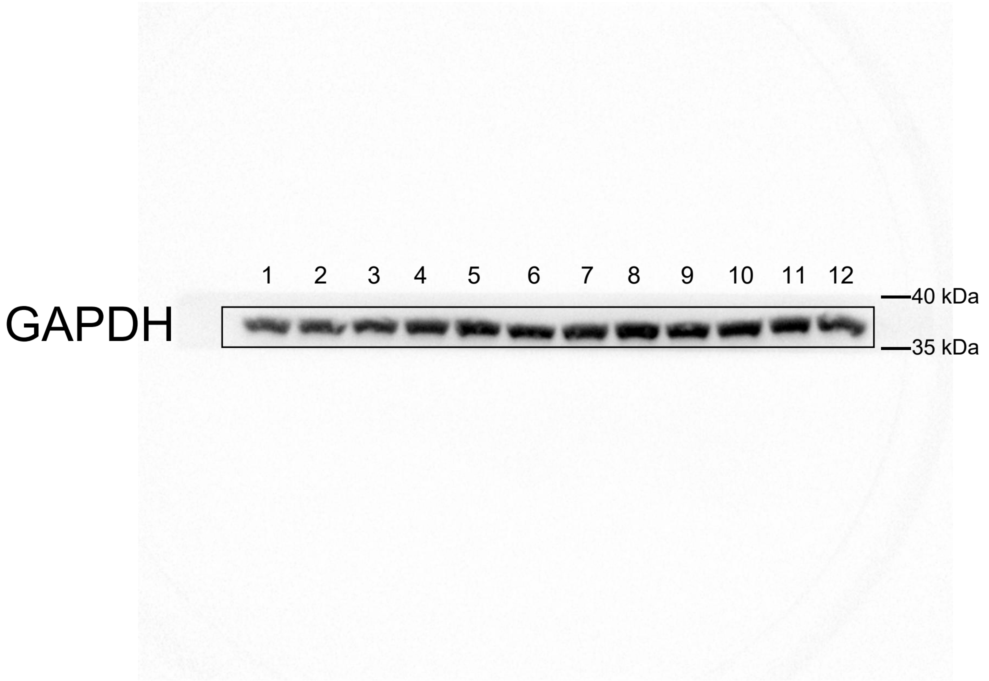

Sup Fig.11c

1. AS 8w
2. AS 16w AAV-NC
3. AS 16w AAV-sgIL-1β
4. AS 8w
5. AS 16w AAV-NC
6. AS 16w AAV-sgIL-1β
7. AS 8w
8. AS 16w AAV-NC
9. AS 16w AAV-sgIL-1β
10. AS 8w
11. AS 16w AAV-NC
12. AS 16w AAV-sgIL-1β
13. AS 8w
14. AS 16w AAV-NC
15. AS 16w AAV-sgIL-1β
16. AS 8w
17. AS 16w AAV-NC
18. AS 16w AAV-sgIL-1β

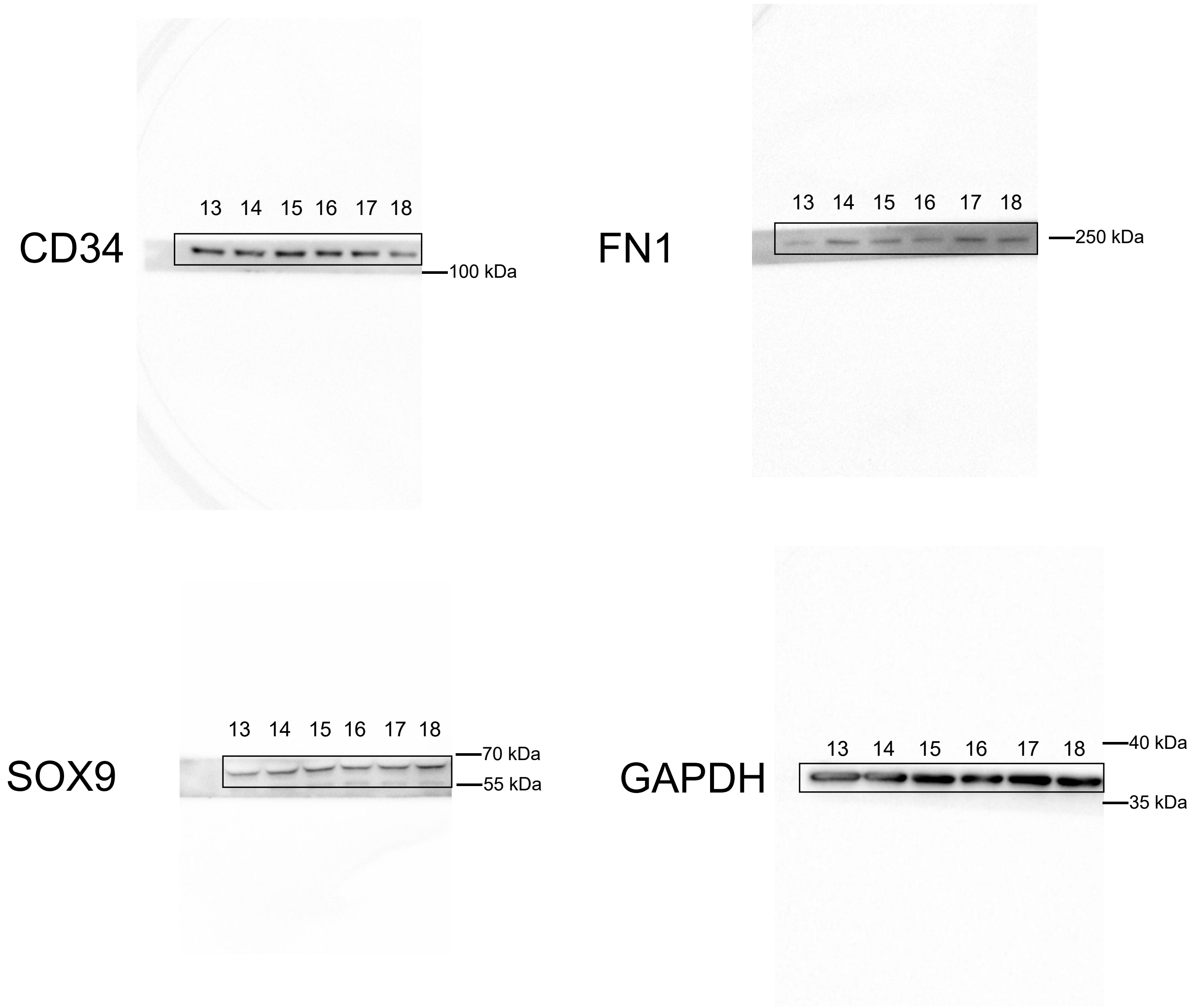

3.original blots with molecular marker ladders

Fig.7 a

1. oxLDL 0 µg/ml
2. oxLDL 10 µg/m
3. oxLDL 25 µg/m
4. oxLDL 50 µg/m
5. oxLDL 75 µg/m
6. oxLDL 100 µg/m
7. oxLDL 125 µg/ml
8. oxLDL 0 µg/ml
9. oxLDL 10 µg/m
10. oxLDL 25 µg/m
11. oxLDL 50 µg/m
12. oxLDL 75 µg/m
13. oxLDL 100 µg/m
14. oxLDL 125 µg/ml
15. oxLDL 0 µg/ml
16. oxLDL 10 µg/m
17. oxLDL 25 µg/m
18. oxLDL 50 µg/m
19. oxLDL 75 µg/m
20. oxLDL 100 µg/m
21. oxLDL 125 µg/ml
22. oxLDL 0 µg/ml
23. oxLDL 10 µg/m
24. oxLDL 25 µg/m
25. oxLDL 50 µg/m
26. oxLDL 75 µg/m
27. oxLDL 100 µg/m
28. oxLDL 125 µg/ml

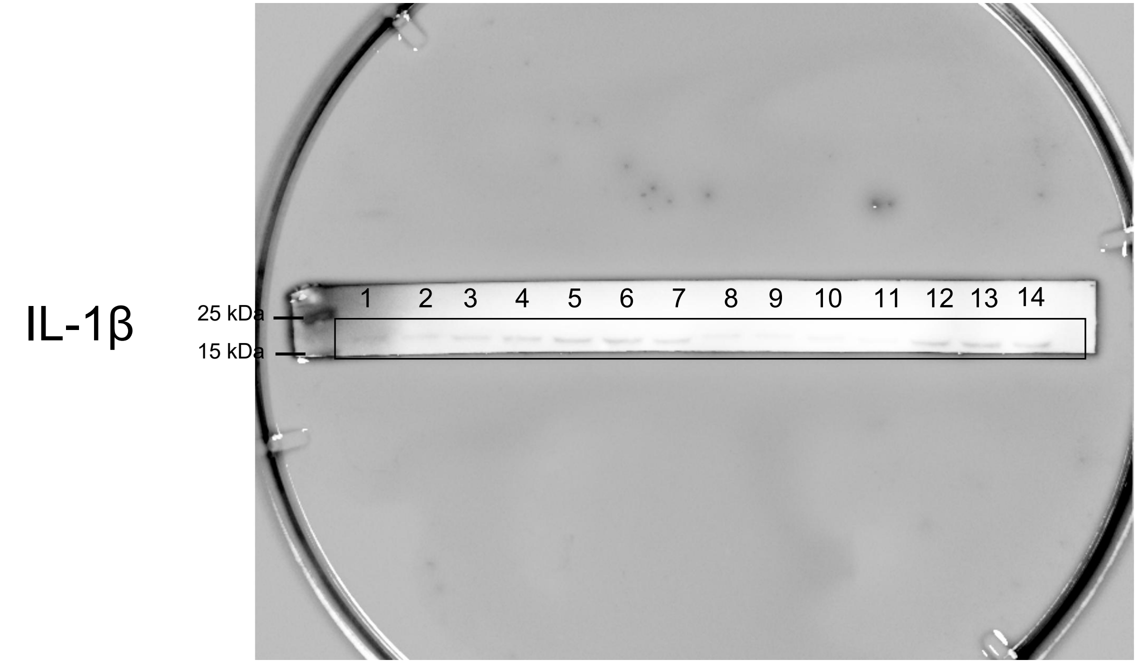

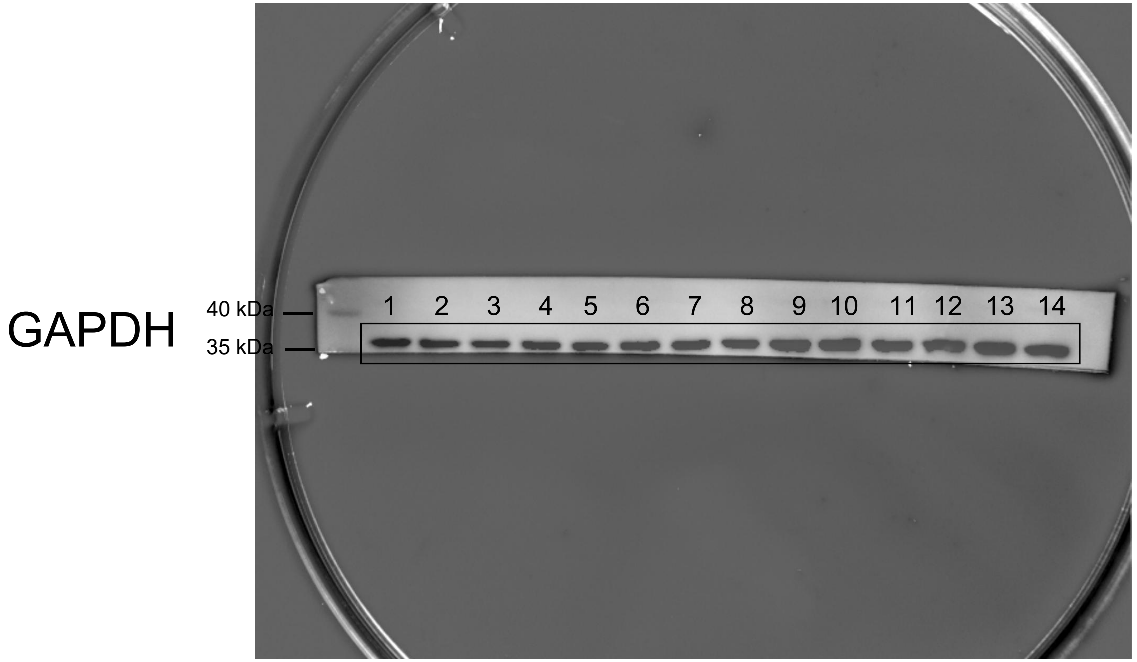

Fig.7 a

1. oxLDL 0 µg/ml
2. oxLDL 10 µg/m
3. oxLDL 25 µg/m
4. oxLDL 50 µg/m
5. oxLDL 75 µg/m
6. oxLDL 100 µg/m
7. oxLDL 125 µg/ml
8. oxLDL 0 µg/ml
9. oxLDL 10 µg/m
10. oxLDL 25 µg/m
11. oxLDL 50 µg/m
12. oxLDL 75 µg/m
13. oxLDL 100 µg/m
14. oxLDL 125 µg/ml
15. oxLDL 0 µg/ml
16. oxLDL 10 µg/m
17. oxLDL 25 µg/m
18. oxLDL 50 µg/m
19. oxLDL 75 µg/m
20. oxLDL 100 µg/m
21. oxLDL 125 µg/ml
22. oxLDL 0 µg/ml
23. oxLDL 10 µg/m
24. oxLDL 25 µg/m
25. oxLDL 50 µg/m
26. oxLDL 75 µg/m
27. oxLDL 100 µg/m
28. oxLDL 125 µg/ml

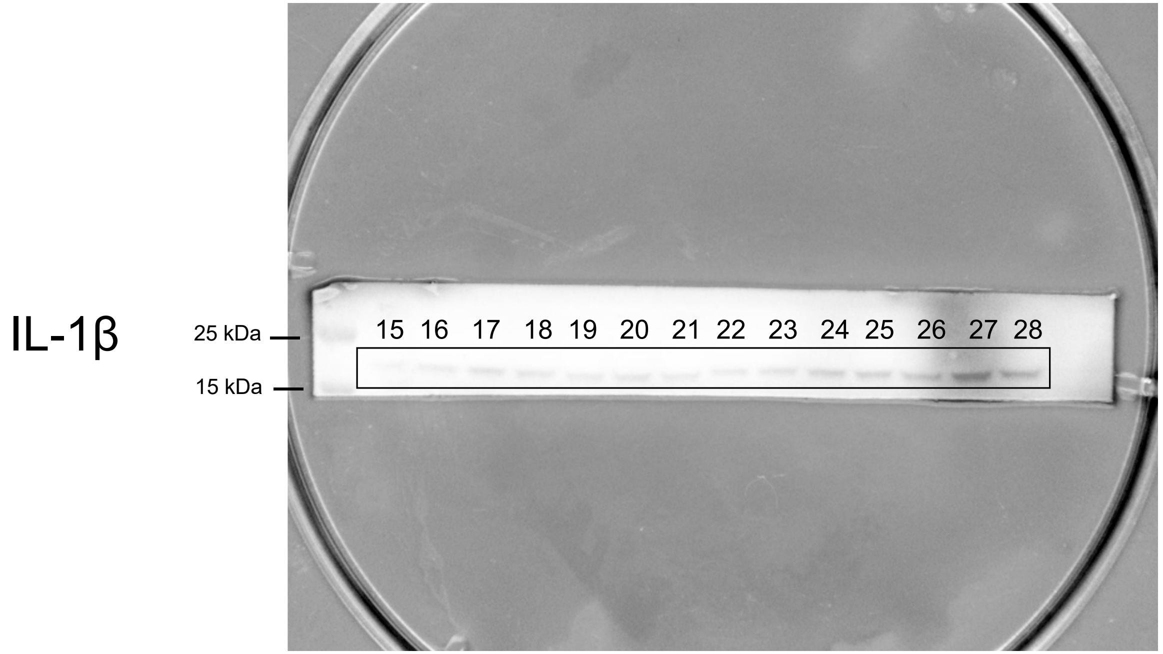

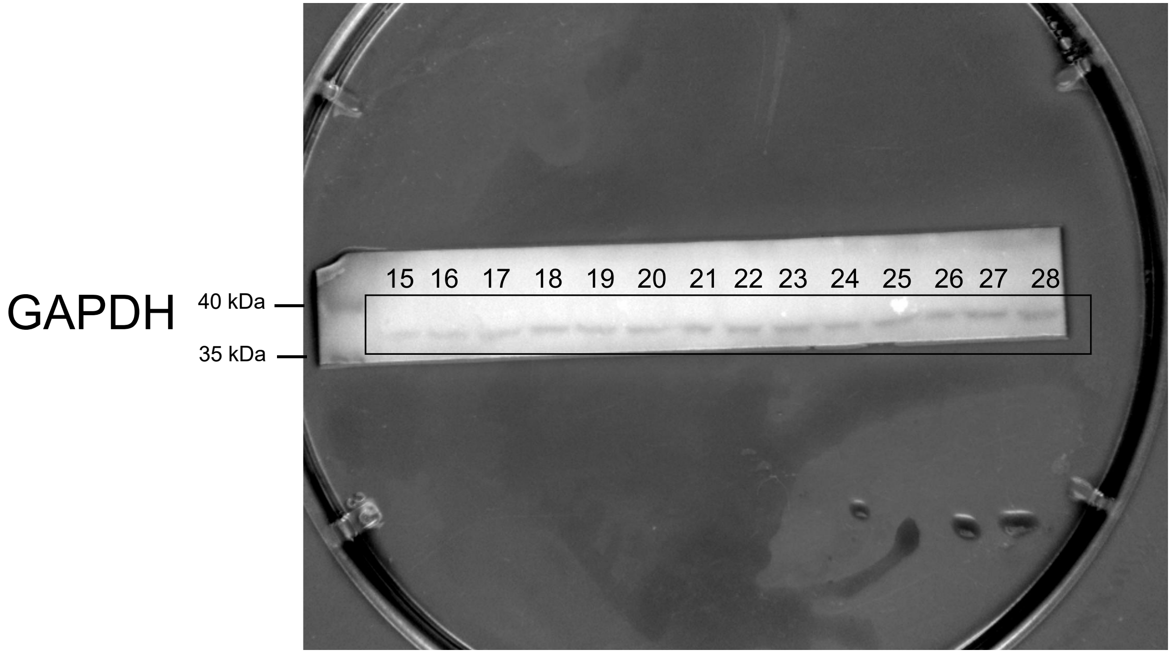

Fig.7 c

1. RM 0h
2. RM 12h
3. RM 24h
4. RM 48h
5. RM 0h
6. RM 12h
7. RM 24h
8. RM 48h
9. RM 0h
10. RM 12h
11. RM 24h
12. RM 48h

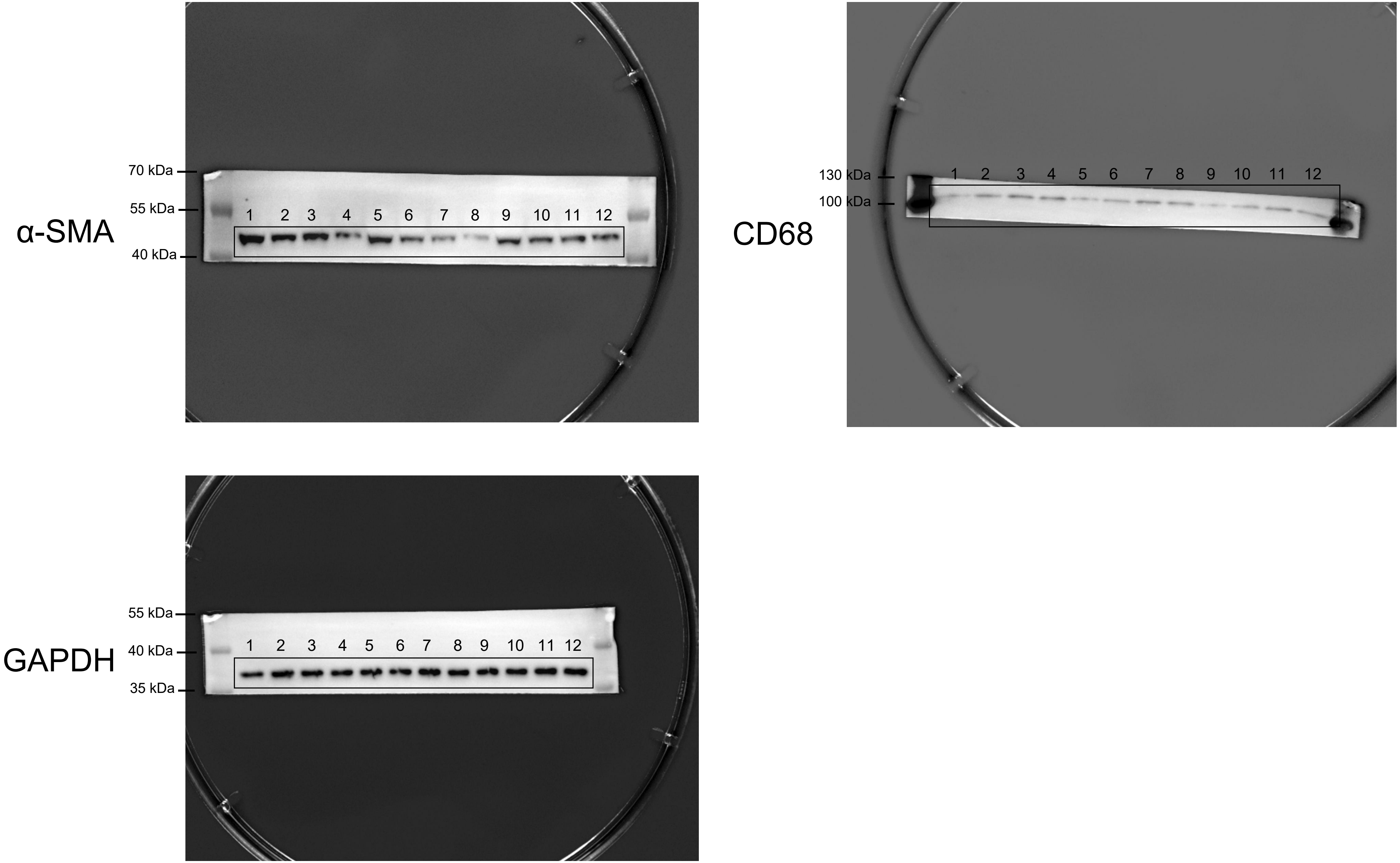

Fig.7 d

1. RM 0h
2. RM 12h
3. RM 24h
4. RM 48h
5. RM 0h
6. RM 12h
7. RM 24h
8. RM 48h
9. RM 0h
10. RM 12h
11. RM 24h
12. RM 48h

p-STAT3

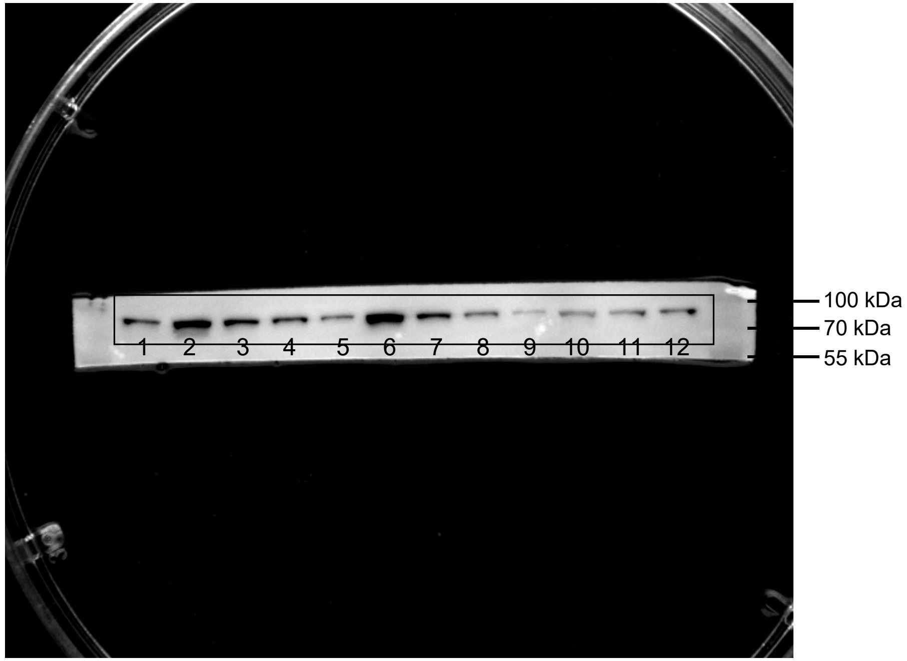

STAT3

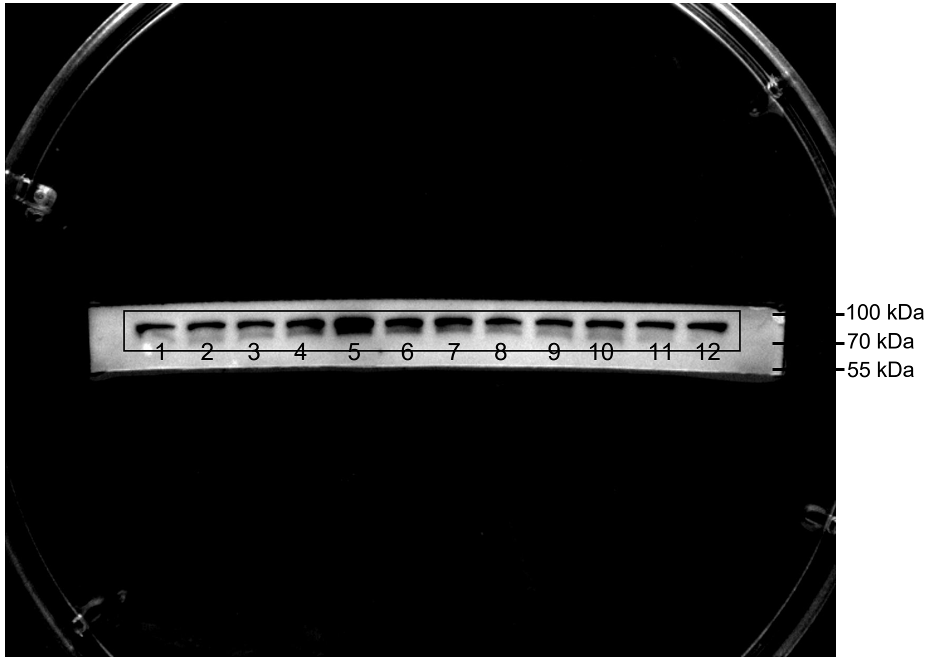

EPAS1

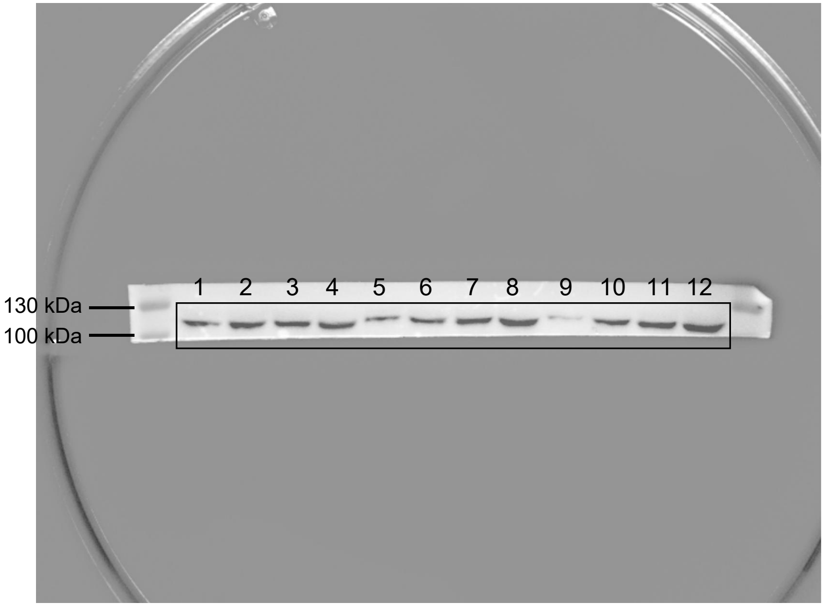

GAPDH

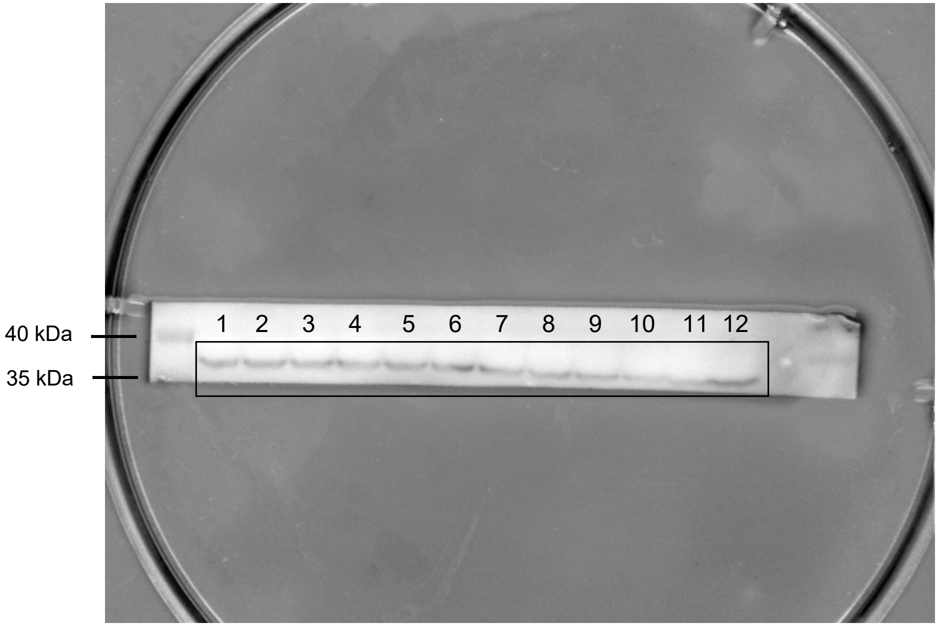

Fig.7 e

1. RM 12h
2. RM 48h
3. RM 12h+IL-1Ra
4. RM 48h+IL-1Ra
5. RM 12h
6. RM 28h
7. RM 12h+IL-1Ra
8. RM 48h+IL-1Ra
9. RM 12h
10. RM 48h
11. RM 12h+IL-1Ra
12. RM 48h+IL-1Ra

p-STAT3

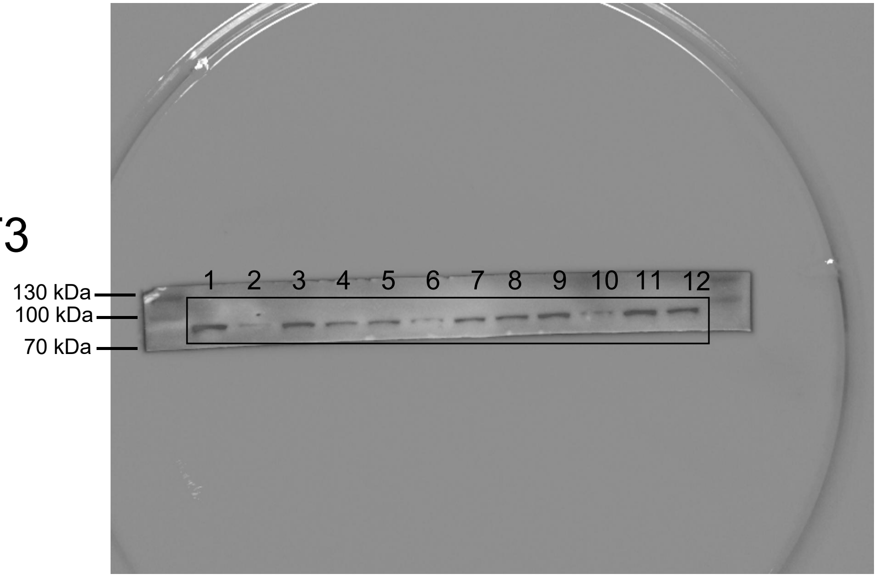

STAT3

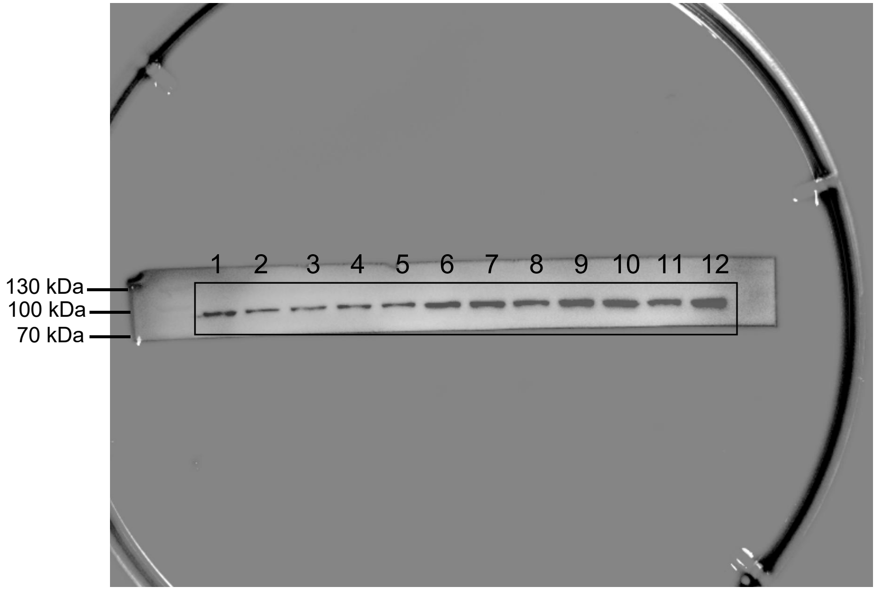

Fig.7 f

1. RM 12h
2. RM 48h
3. RM 48h+IL-1Ra
4. RM 48h+Colivelin
5. RM 48h+IL-1Ra+Stattic
6. RM 12h
7. RM 48h
8. RM 48h+IL-1Ra
9. RM 48h+Colivelin
10. RM 48h+IL-1Ra+Stattic

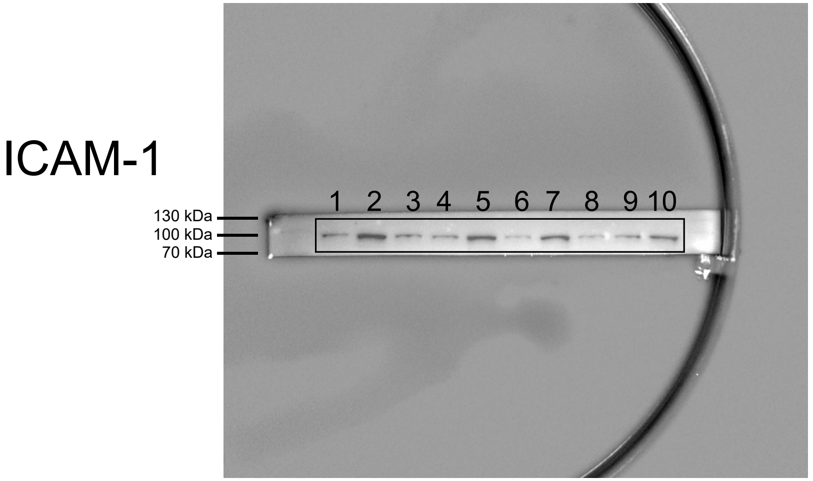

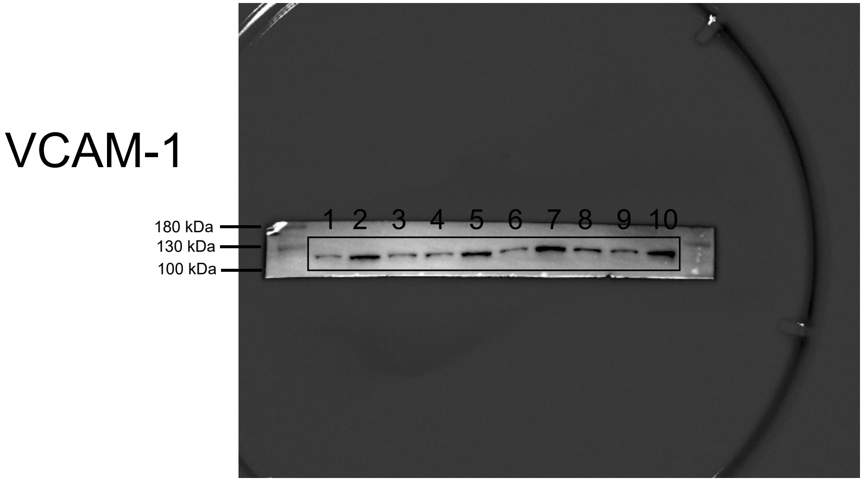

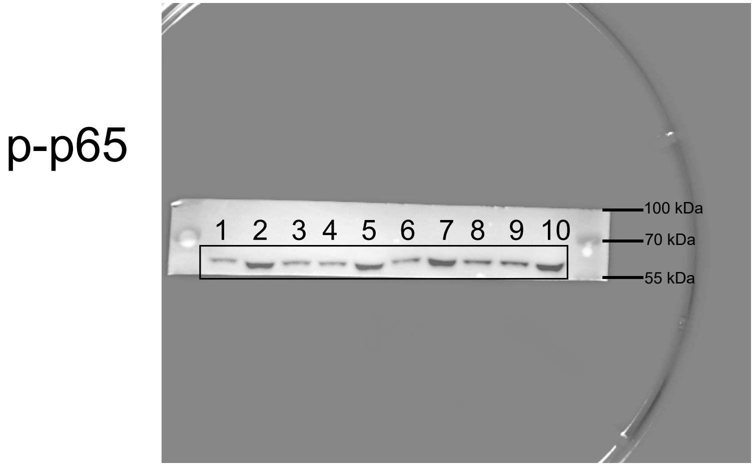

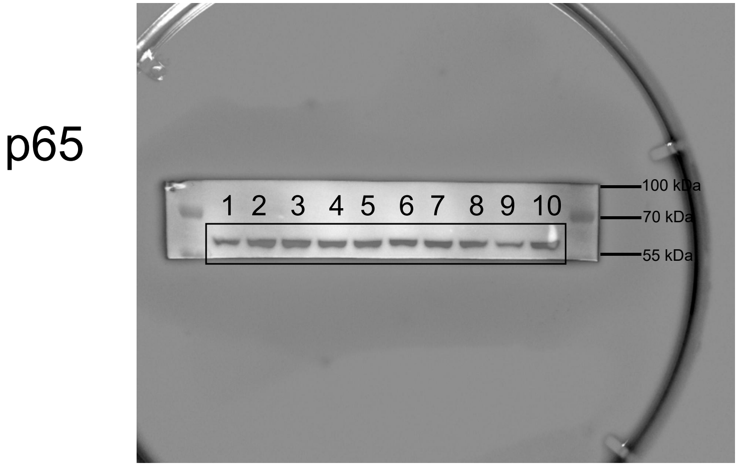

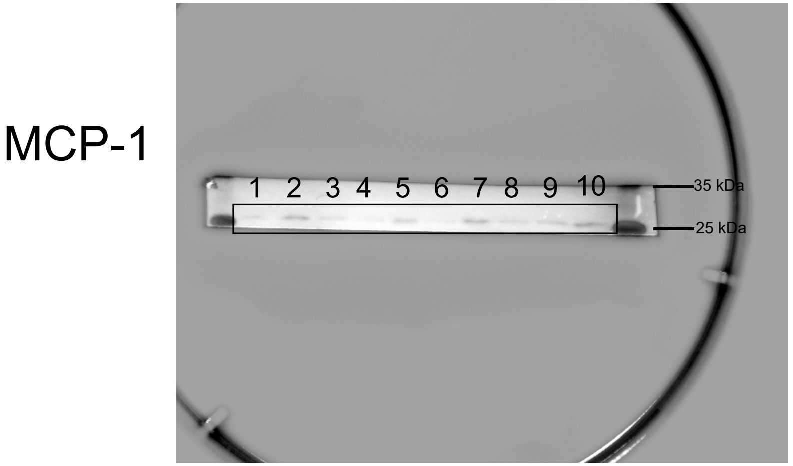

Fig.7 f

1. RM 12h
2. RM 48h
3. RM 48h+IL-1Ra
4. RM 48h+Colivelin
5. RM 48h+IL-1Ra+Stattic
6. RM 12h
7. RM 48h
8. RM 48h+IL-1Ra
9. RM 48h+Colivelin
10. RM 48h+IL-1Ra+Stattic

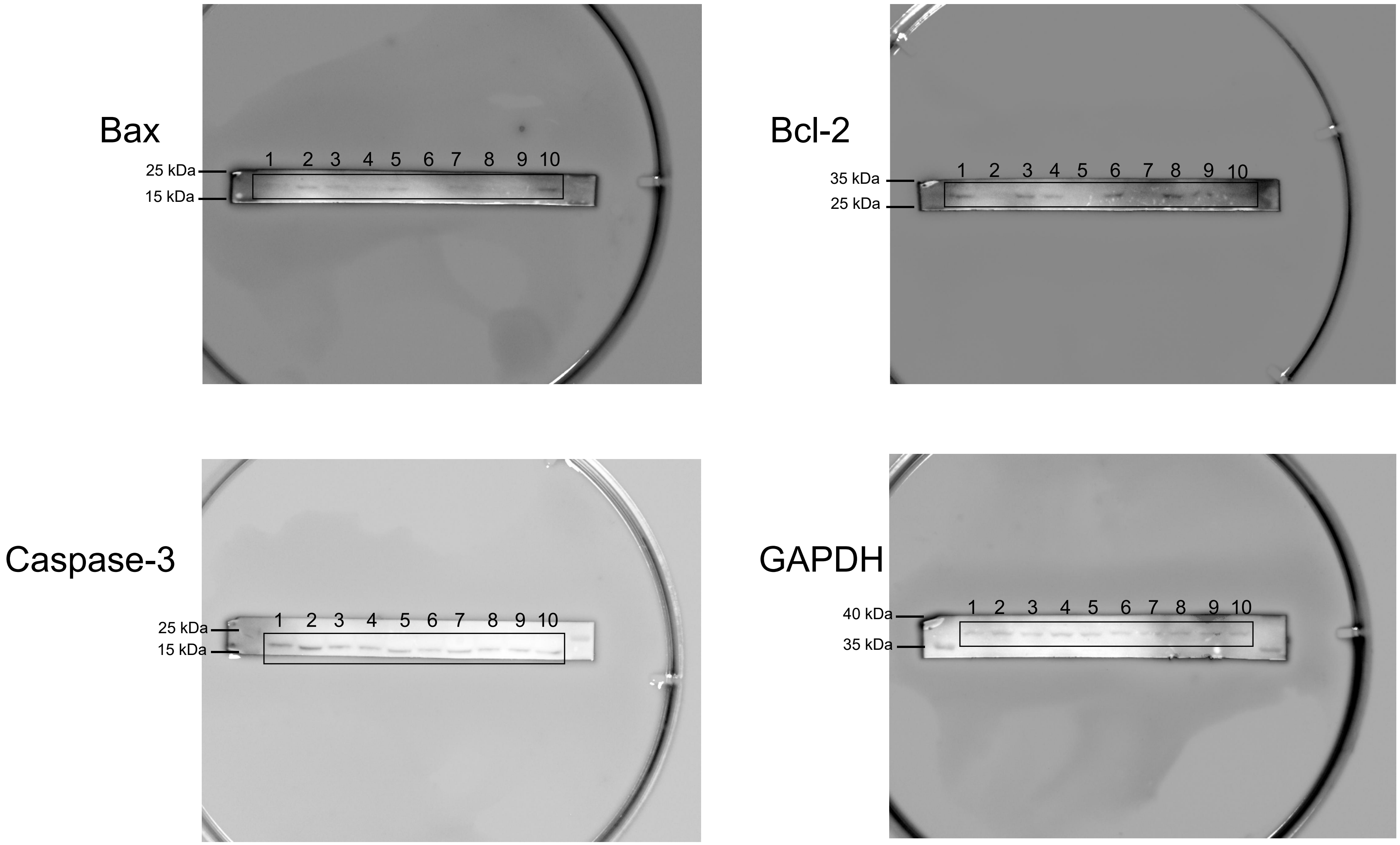

Fig.7 f

11. RM 12h
12. RM 48h
13. RM 48h+IL-1Ra
14. RM 48h+Colivelin
15. RM 48h+IL-1Ra+Stattic
16. RM 12h
17. RM 48h
18. RM 48h+IL-1Ra
19. RM 48h+Colivelin
20. RM 48h+IL-1Ra+Stattic

ICAM-1

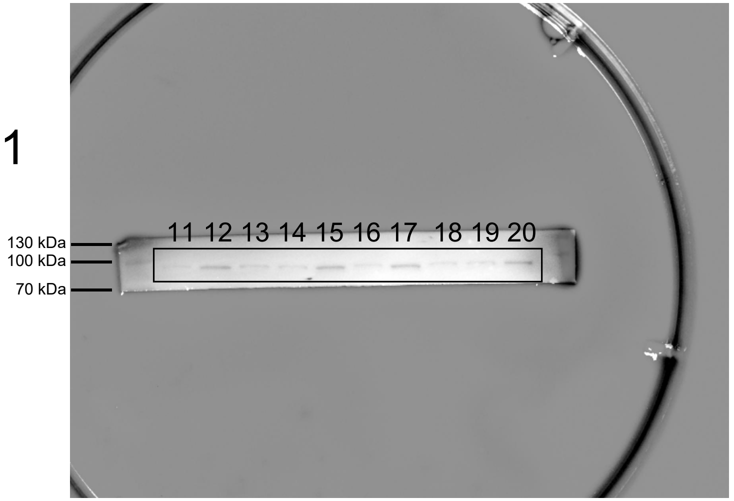

VCAM-1

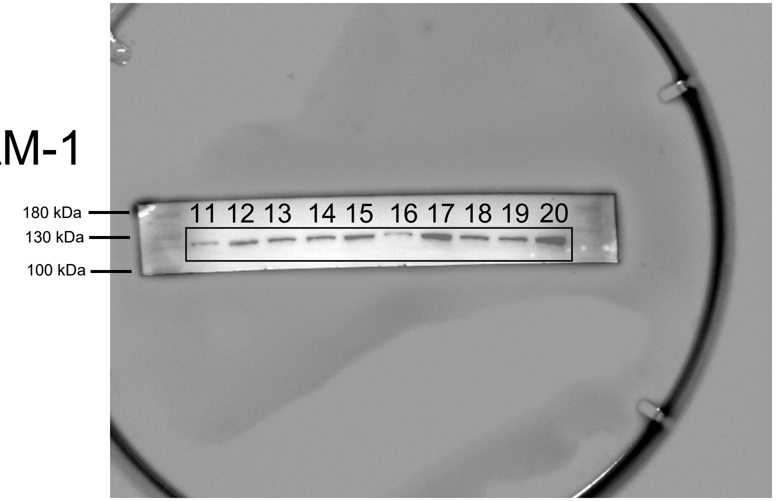

p-p65

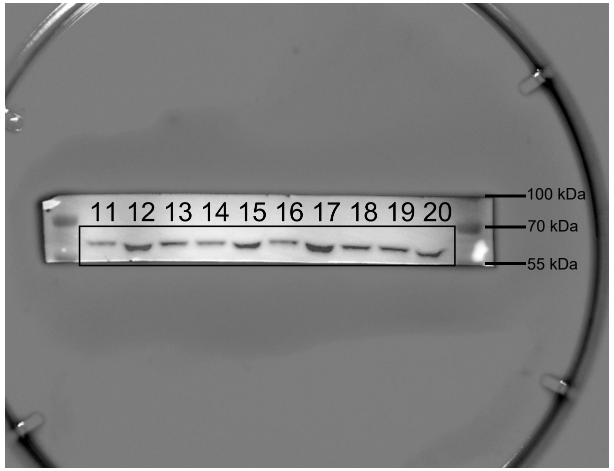

p65

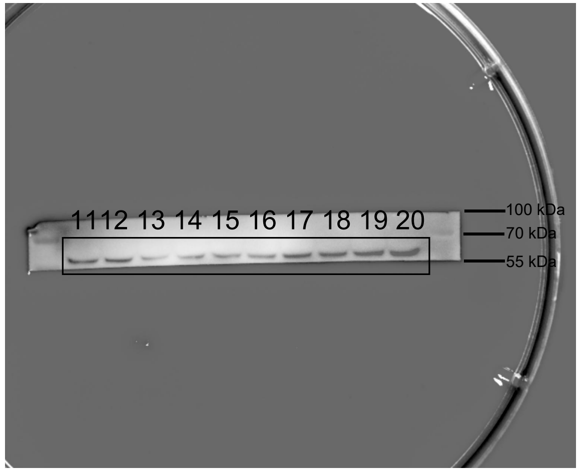

MCP-1

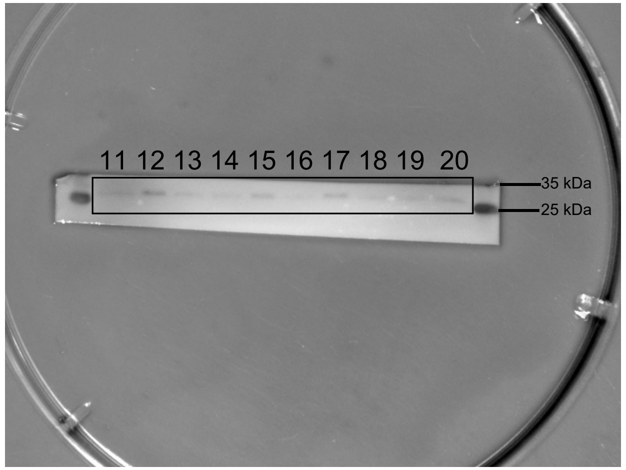

Fig.7 f

11. RM 12h
12. RM 48h
13. RM 48h+IL-1Ra
14. RM 48h+Colivelin
15. RM 48h+IL-1Ra+Stattic
16. RM 12h
17. RM 48h
18. RM 48h+IL-1Ra
19. RM 48h+Colivelin
20. RM 48h+IL-1Ra+Stattic

Bax

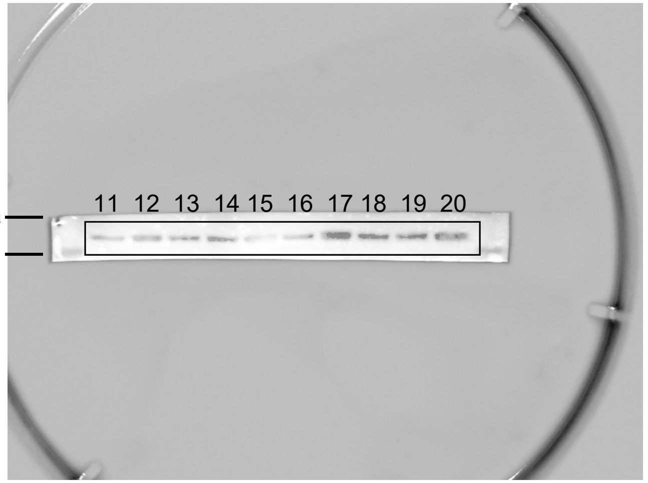

Bcl-2

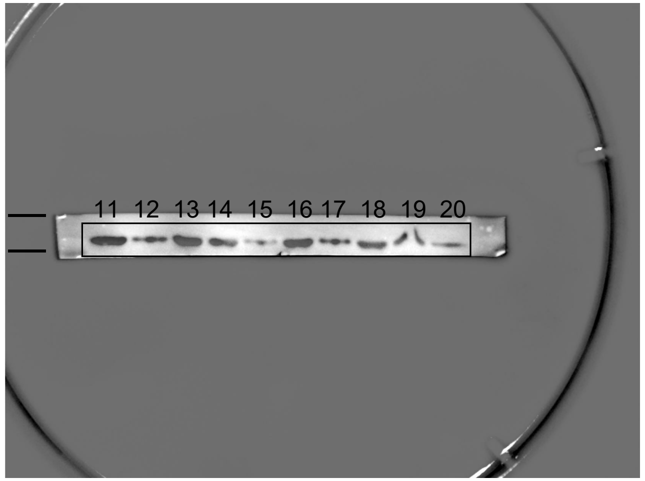

Caspase-3

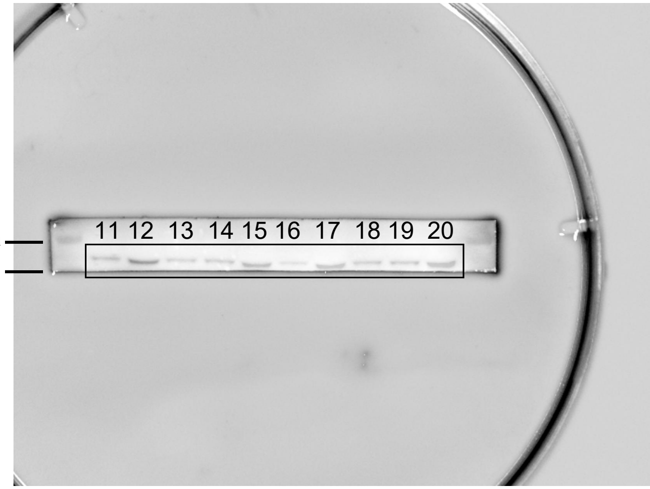

GAPDH

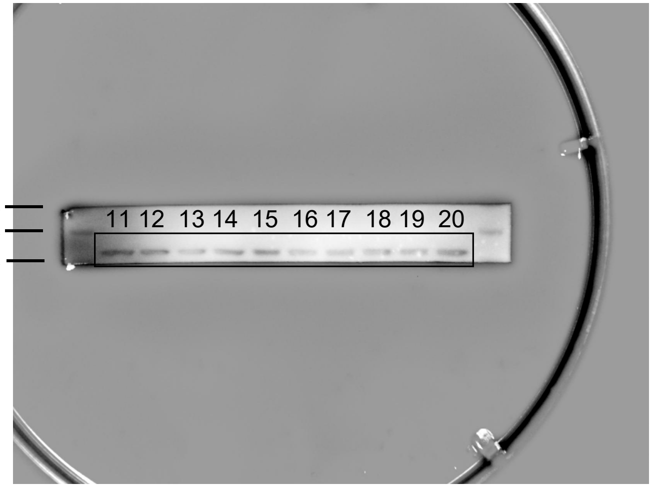

Fig.8 a

1. AS 8w
2. AS 16w AAV-NC
3. AS 16w AAV-sgIL-1β
4. AS 8w
5. AS 16w AAV-NC
6. AS 16w AAV-sgIL-1β
7. AS 8w
8. AS 16w AAV-NC
9. AS 16w AAV-sgIL-1β
10. AS 8w
11. AS 16w AAV-NC
12. AS 16w AAV-sgIL-1β

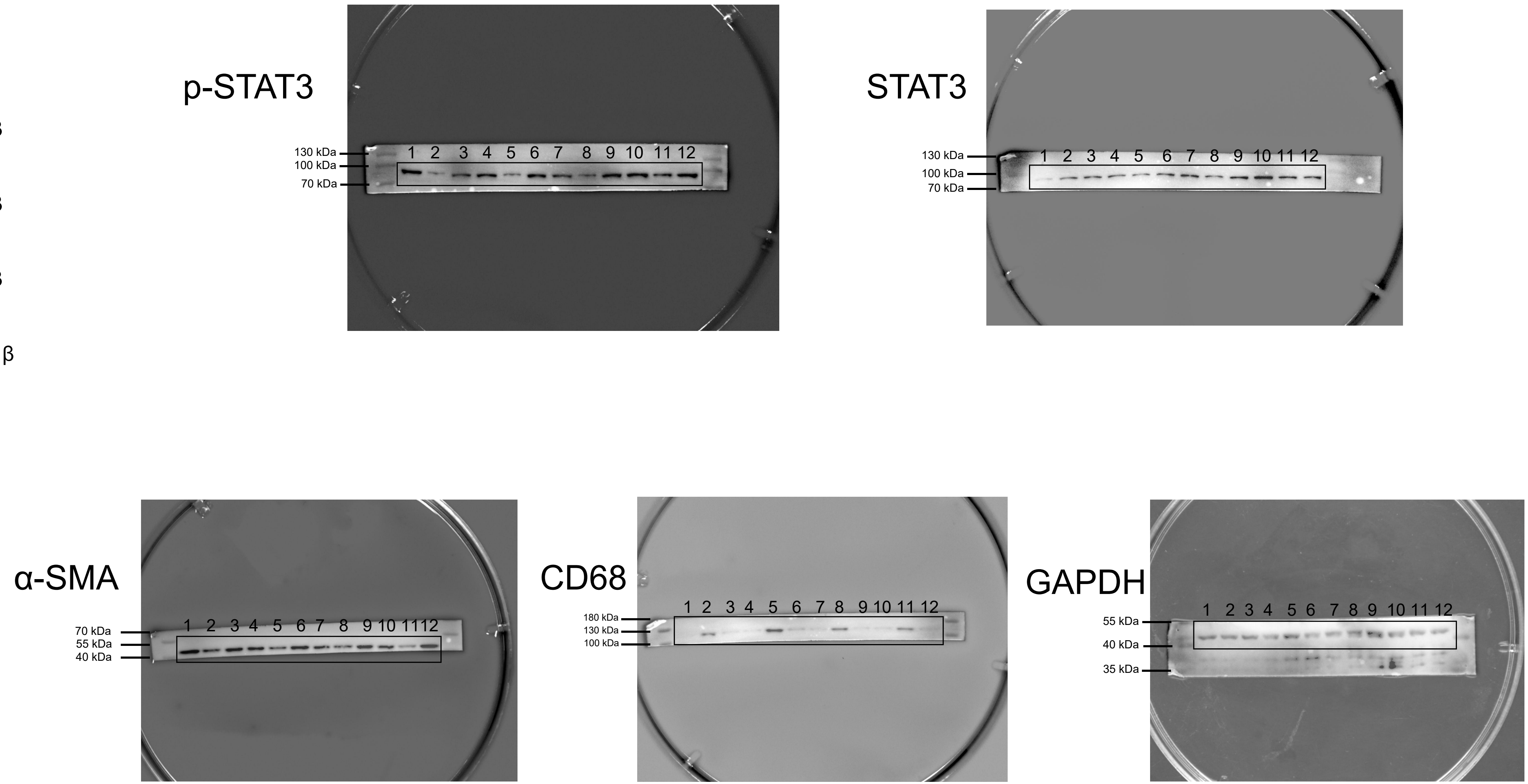

Sup Fig.9 b

1. MM 12h
2. MM 48h
3. MM 12h+IL-1Ra
4. MM 48h+IL-1Ra
5. MM 12h
6. MM 48h
7. MM 12h+IL-1Ra
8. MM 48h+IL-1Ra
9. MM 12h
10. MM 48h
11. MM 12h+IL-1Ra
12. MM 48h+IL-1Ra

p-STAT3

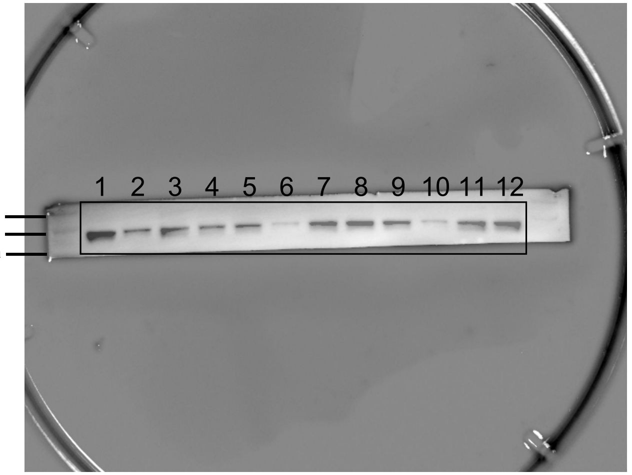

STAT3

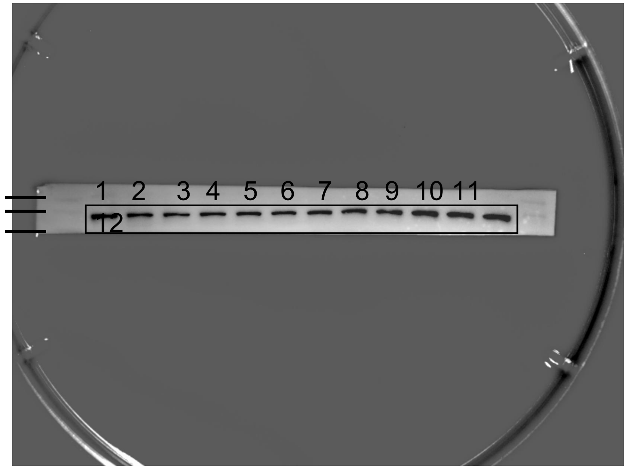

α-SMA

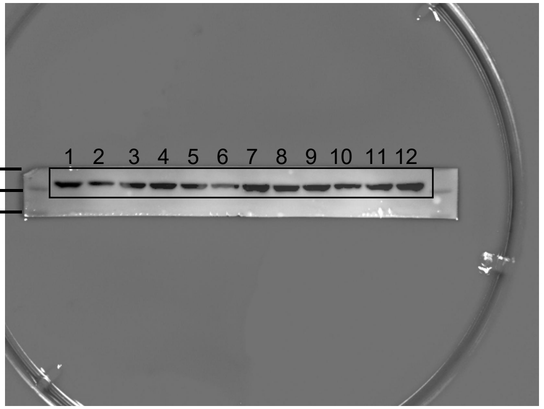

CD68

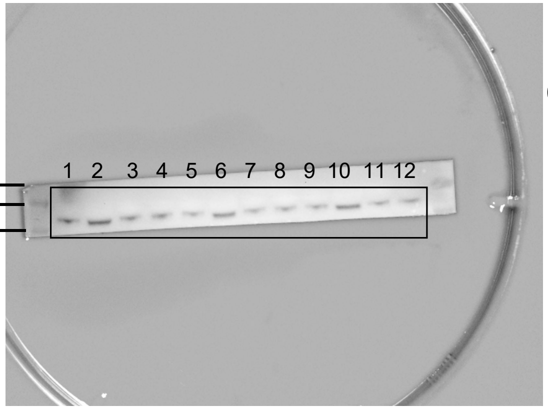

GAPDH

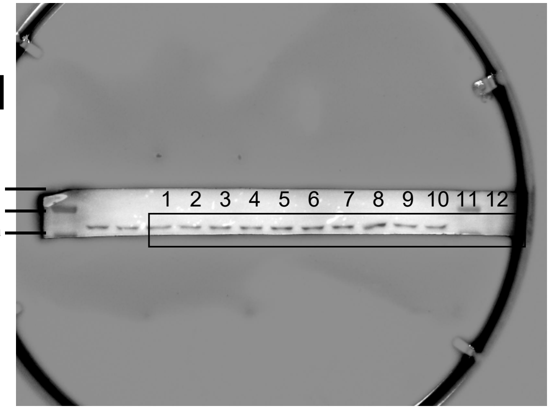

Sup Fig.9 e

1. MM 12h
2. MM 48h
3. MM 12h+IL-1Ra
4. MM 48h+IL-1Ra
5. MM 12h
6. MM 48h
7. MM 12h+IL-1Ra
8. MM 48h+IL-1Ra
9. MM 12h
10. MM 48h
11. MM 12h+IL-1Ra
12. MM 48h+IL-1Ra

EPAS1

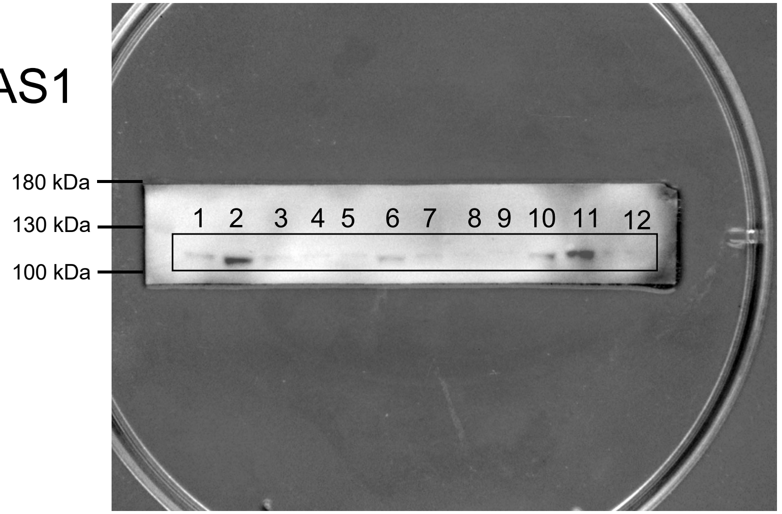

GAPDH

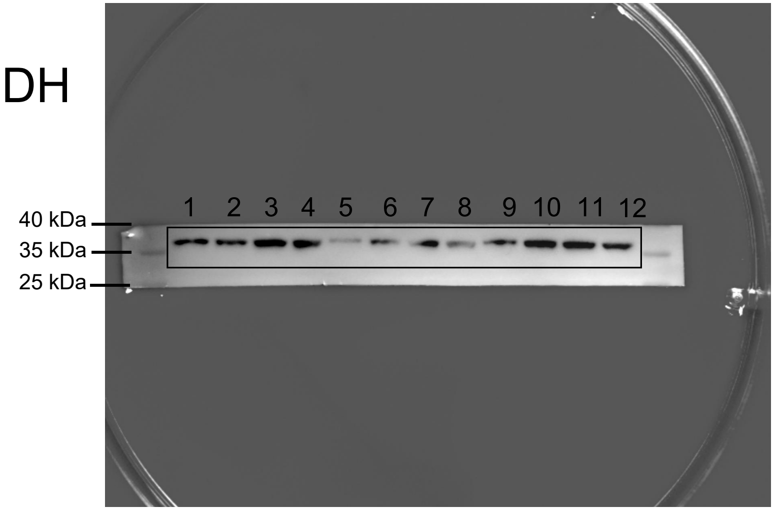

Sup Fig.11 a

1. AS 8w
2. AS 16w AAV-NC
3. AS 16w AAV-sgIL-1β
4. AS 8w
5. AS 16w AAV-NC
6. AS 16w AAV-sgIL-1β
7. AS 8w
8. AS 16w AAV-NC
9. AS 16w AAV-sgIL-1β
10. AS 8w
11. AS 16w AAV-NC
12. AS 16w AAV-sgIL-1β

IL-1β

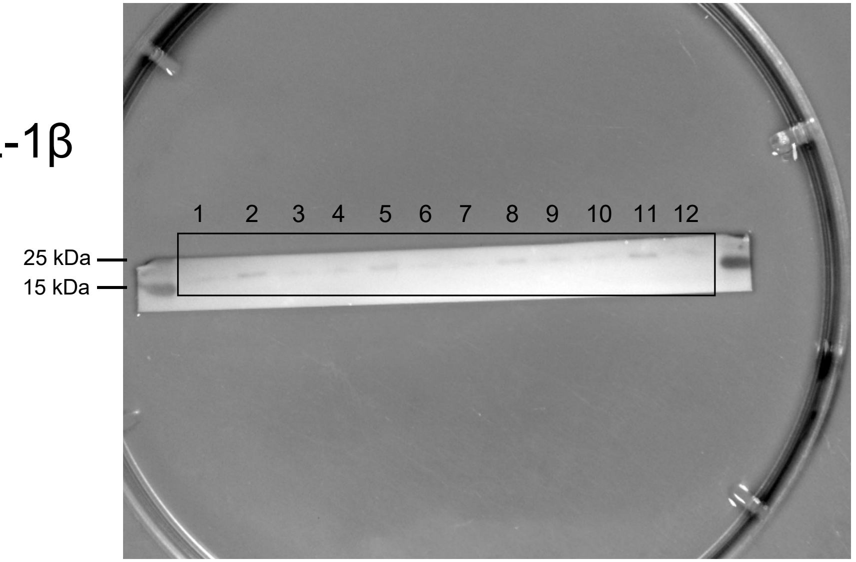

GAPDH

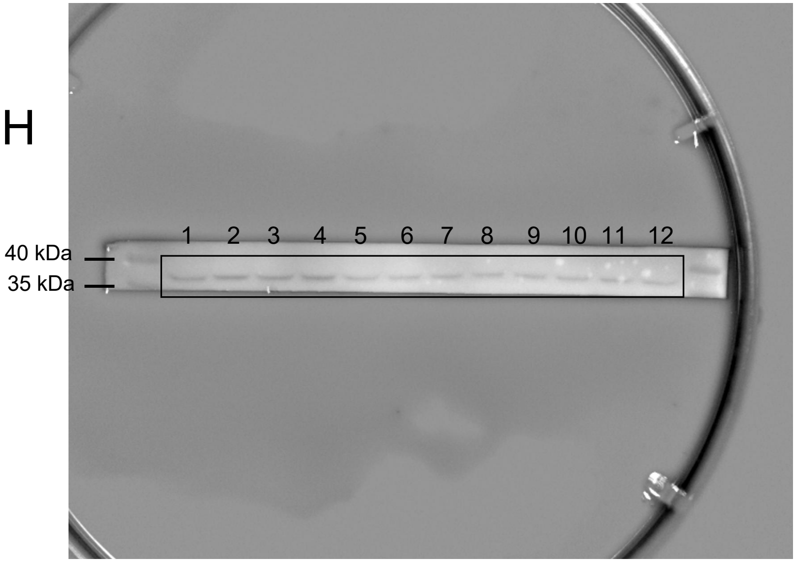

Sup Fig.11 c

1. AS 8w
2. AS 16w AAV-NC
3. AS 16w AAV-sgIL-1β
4. AS 8w
5. AS 16w AAV-NC
6. AS 16w AAV-sgIL-1β
7. AS 8w
8. AS 16w AAV-NC
9. AS 16w AAV-sgIL-1β
10. AS 8w
11. AS 16w AAV-NC
12. AS 16w AAV-sgIL-1β

CD34

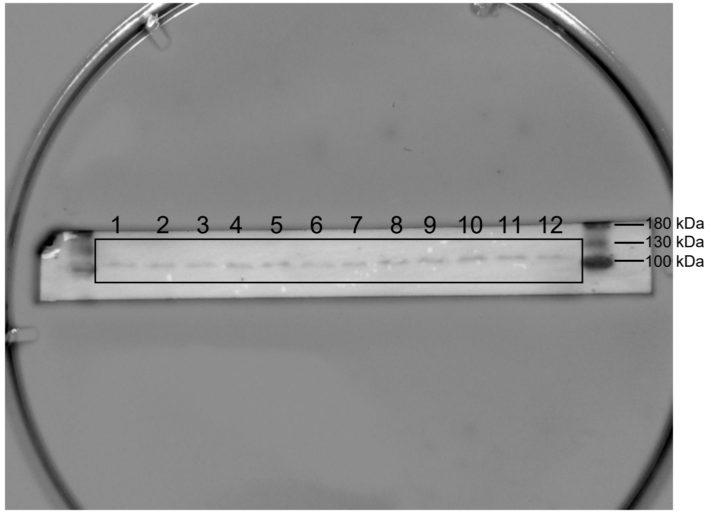

FN1

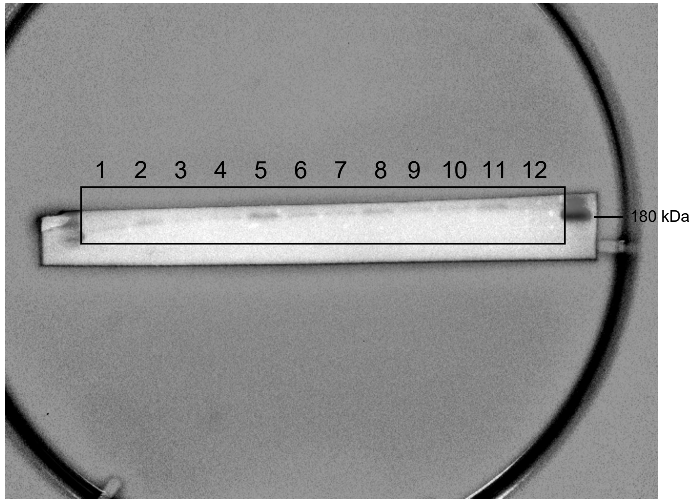

SOX9

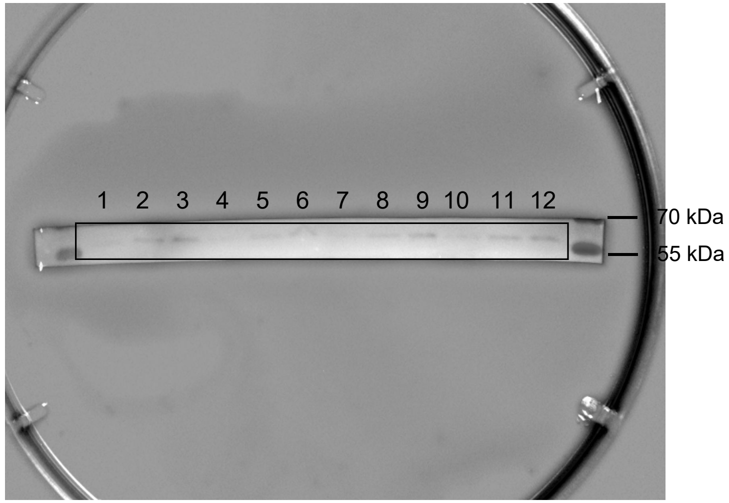

GAPDH

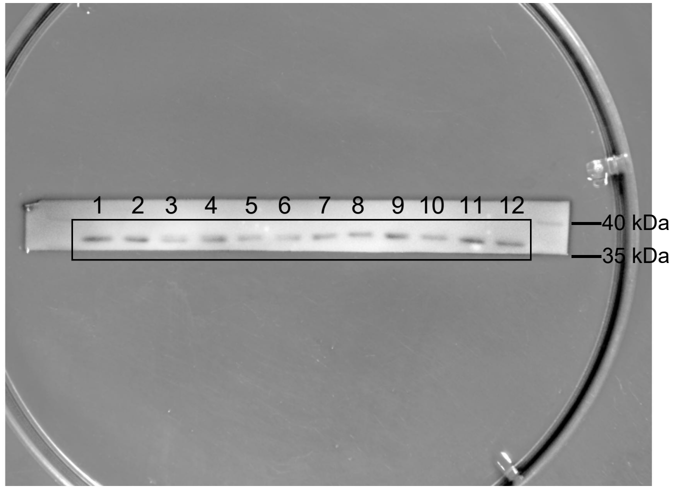

Supplement: Supplementary file 2 — Supplementary Information [file 42003_2022_4255_MOESM2_ESM.pdf]
