# Supplementary material for: Macrophages regulate vascular smooth muscle cell function during atherosclerosis progression through IL-1β/STAT3 signaling
Source: Commun Biol. 2022 Dec 1;5:1316. doi: 10.1038/s42003-022-04255-2 (PMC9715630; doi:10.1038/s42003-022-04255-2)
Supplement: Supplementary file 3 — Description of Additional Supplementary Files [file 42003_2022_4255_MOESM3_ESM.pdf]

Description of Additional Supplementary Files

File name: Supplementary Data 1

Description: The supplementary tables in our manuscript.

File name: Supplementary Data 2

Description: The original values for the blots in both figures and supplementary figures.
