# Supplementary material for: Macrophages regulate vascular smooth muscle cell function during atherosclerosis progression through IL-1β/STAT3 signaling
Source: Commun Biol. 2022 Dec 1;5:1316. doi: 10.1038/s42003-022-04255-2 (PMC9715630; doi:10.1038/s42003-022-04255-2)
Supplement: Supplementary file 6 — Reporting Summary [file 42003_2022_4255_MOESM6_ESM.pdf]

nature portfolio

Corresponding author(s): *Double-blind peer review submissions: write DBPR and your manuscript number here instead of author names.*

Last updated by author(s): *YYYY-MM-DD*

Reporting Summary

Nature Portfolio wishes to improve the reproducibility of the work that we publish. This form provides structure for consistency and transparency in reporting. For further information on Nature Portfolio policies, see our Editorial Policies and the Editorial Policy Checklist.

Statistics

For all statistical analyses, confirm that the following items are present in the figure legend, table legend, main text, or Methods section.

n/a	Confirmed	
☐	☒	The exact sample size (n) for each experimental group/condition, given as a discrete number and unit of measurement
☒	☐	A statement on whether measurements were taken from distinct samples or whether the same sample was measured repeatedly
☐	☒	The statistical test(s) used AND whether they are one- or two-sided<br>*Only common tests should be described solely by name; describe more complex techniques in the Methods section.*
☒	☐	A description of all covariates tested
☐	☒	A description of any assumptions or corrections, such as tests of normality and adjustment for multiple comparisons
☐	☒	A full description of the statistical parameters including central tendency (e.g. means) or other basic estimates (e.g. regression coefficient) AND variation (e.g. standard deviation) or associated estimates of uncertainty (e.g. confidence intervals)
☐	☒	For null hypothesis testing, the test statistic (e.g. F, t, r) with confidence intervals, effect sizes, degrees of freedom and P value noted<br>*Give P values as exact values whenever suitable.*
☒	☐	For Bayesian analysis, information on the choice of priors and Markov chain Monte Carlo settings
☒	☐	For hierarchical and complex designs, identification of the appropriate level for tests and full reporting of outcomes
☒	☐	Estimates of effect sizes (e.g. Cohen's d, Pearson's r), indicating how they were calculated

Our web collection on statistics for biologists contains articles on many of the points above.

Software and code

Policy information about availability of computer code

Data collection	Data were downloaded from GEO database (https://www.ncbi.nlm.nih.gov/geo/), including GSE159677 and GSE43292.
Data analysis	R software (3.6.1 or 4.0.3) and GraphPad Prism software (version 5.0 or 8.4.0).

For manuscripts utilizing custom algorithms or software that are central to the research but not yet described in published literature, software must be made available to editors and reviewers. We strongly encourage code deposition in a community repository (e.g. GitHub). See the Nature Portfolio guidelines for submitting code & software for further information.

Data

Policy information about availability of data

All manuscripts must include a data availability statement. This statement should provide the following information, where applicable:

- Accession codes, unique identifiers, or web links for publicly available datasets
- A description of any restrictions on data availability
- For clinical datasets or third party data, please ensure that the statement adheres to our policy

The publicly available datasets analyzed in this study can be found at: https://www.ncbi.nlm.nih.gov/geo/. In silico analysis was performed using custom R scripts designed for this study. R scripts are available once from the corresponding author on reasonable request.

Human research participants

Policy information about studies involving human research participants and Sex and Gender in Research.

Reporting on sex and gender	N/A
Population characteristics	N/A
Recruitment	N/A
Ethics oversight	N/A

Note that full information on the approval of the study protocol must also be provided in the manuscript.

Field-specific reporting

Please select the one below that is the best fit for your research. If you are not sure, read the appropriate sections before making your selection.

☒ Life sciences ☐ Behavioural & social sciences ☐ Ecological, evolutionary & environmental sciences

For a reference copy of the document with all sections, see nature.com/documents/nr-reporting-summary-flat.pdf

Life sciences study design

All studies must disclose on these points even when the disclosure is negative.

Sample size	The sample sizes in scRNA-seq and bulk microarray analysis were predetermined according to the public datasets. Sample sizes in experiments were predetermined based on statistical power calculations or convention in the field. The exact sample size is given in the figure legends
Data exclusions	No data were excluded
Replication	In this study, the repetition was at least 3 to ensure the stability of individual sample.
Randomization	In in vivo studies,eighteen ApoE -/- mice were randomly grouped into AS16w, AS 16w+AAV-NC, and AS 16w+AAV-sgIL-1β.
Blinding	In in vivo studies, the group information is not known for the operators during western blot analysis.

Reporting for specific materials, systems and methods

We require information from authors about some types of materials, experimental systems and methods used in many studies. Here, indicate whether each material, system or method listed is relevant to your study. If you are not sure if a list item applies to your research, read the appropriate section before selecting a response.

Materials & experimental systems

n/a	Involved in the study	
☐	☒	Antibodies
☐	☒	Eukaryotic cell lines
☒	☐	Palaeontology and archaeology
☐	☒	Animals and other organisms
☒	☐	Clinical data
☒	☐	Dual use research of concern

Methods

n/a	Involved in the study	
☒	☐	ChIP-seq
☒	☐	Flow cytometry
☒	☐	MRI-based neuroimaging

Antibodies

Antibodies used	Primary antibodies against p-STAT3(Tyr705) (#9145), STAT3 (#30835), p-p65(Ser536) (#3033), p65 (#8242), and α-SMA (#19245) were purchased from Cell Signaling Technology (Beverly, MA, USA). Primary antibodies against IL-1β (sc-12742), CD34 (sc-7324) , and EPAS-1 (sc-13596) were purchased from Santa Cruz Biotechnology (Santa Cruz, CA, USA). Primary antibodies against CD68 (28058-1-AP), ICAM-1 (60299-1-Ig), VCAM-1 (66294-1-Ig), MCP-1 (66272-1-Ig), Bax (50599-2-Ig), Bcl-2 (12789-1-AP), cleaved-caspase-3 (19677-1-AP), GAPDH (10494-1-AP), FN1 (15613-1-AP), and SOX9 (67439-1-Ig) as well as secondary antibodies (Goat anti-mouse, SA00001-1; Goat anti-rabbit, SA00001-2) were purchased from Proteintech Group (Chicago, IL, USA).

Validation	The antibodies were validated by the official website.<br>Cell Signaling Technology: https://www.cellsignal.com/<br>Santa Cruz Biotechnology: https://www.scbt.com/zh/home/<br>Proteintech Group: https://www.ptgcn.com/

Eukaryotic cell lines

Policy information about cell lines and Sex and Gender in Research

Cell line source(s)	Primary bone marrow derived macrophages (BMDM) from femurs of eight weeks old male C57BL/6J mice<br>Primary vascular smooth muscle cells (VSMCs) were isolated from the aorta of wild-type male C57BL/6J mice<br>RAW264.7 (mouse macrophage cell line) cells
Authentication	RAW264.7 were authenticated by STR authentication
Mycoplasma contamination	All cell lines were tested negative for mycoplasma contamination
Commonly misidentified lines<br>(See ICLAC register)	N/A

Animals and other research organisms

Policy information about studies involving animals; ARRIVE guidelines recommended for reporting animal research, and Sex and Gender in Research

Laboratory animals	Apolipoprotein E–deficient (ApoE −/−) male C57BL/6J mice
Wild animals	Male apolipoprotein E–deficient (ApoE−/−) mice on C57BL/6J background aged 8 weeks were fed a high-fat diet (HFD) only for 8 or 16 weeks respectively to simulate early and late atherosclerotic plaques. At the end point, mice were anesthetized with ketamine/xylazine, blood, aorta, and carotid artery were harvested after PBS perfusion.
Reporting on sex	Only male mice were included in our experiments because of effects of estrogen on artery disease are protective and positive.
Field-collected samples	The study did not involve samples collected from field.
Ethics oversight	All animal experiments were approved by the local Ethics committee (The First Affiliated Hospital of Chongqing Medical University; License Number: 2021-604)

Note that full information on the approval of the study protocol must also be provided in the manuscript.
